# Targeted‐Penetrating Biomimetic Hybrid Transfersomes for Enhanced Transdermal Gene Transfection in Hypertrophic Scar Therapy

**DOI:** 10.1002/advs.77975

**Published:** 2026-09-27

**Authors:** Hui Xing, Ziyi Zhao, Yuhui Yang, Yichen Lin, Yinglin Xuan, Ziyang Wang, Kai Lv, Xiaoxu Zhu, Zhi Zhang, Dong Ma

**Affiliations:** ^1^ Department of Burn and Plastic Surgery Guangzhou Red Cross Hospital Jinan University Guangzhou China; ^2^ State Key Laboratory of Bioactive Molecules and Druggability Assessment, Institute of Biomedical Engineering Jinan University Guangzhou China; ^3^ MOE Key Laboratory of Tumor Molecular Biology Jinan University Guangzhou China

**Keywords:** active targeting, hybrid transfersomes, hypertrophic scar, siRNA therapy, transdermal transfection

## Abstract

Hypertrophic scar (HS) is a recurrent and inflammatory dermal fibrotic disorder, characterized by abnormal fibroblast proliferation and excessive extracellular matrix (ECM) deposition. Affecting over 310 million patients worldwide, while effective strategies to halt progression remain lacking. The TGF‐β–mediated fibrotic cascade is the central driver of HS, and siRNA‐based therapeutics offer a promising gene‐level intervention owing to their high sequence specificity and low off‐target risk. However, siRNA translation is limited by rapid degradation, poor transfection, insufficient targeting, and the dense scar barrier that restricts noninvasive delivery. Proteomic analysis revealed that hypertrophic scar fibroblasts (HSFs) exhibit enhanced bioadhesion and protein‐binding functions, with Thy‐1 membrane glycoprotein highly and specifically expressed, providing a target for HSFs‐active delivery. We hybridized HSFs membranes with cationic transfersomes using ultrasonic co‐extrusion and achieved efficient loading of TGF‐β1 siRNA through electrostatic self‐assembly. This membrane‐hybridized platform enabled multipathway transdermal delivery of siRNA via intercellular transport, transcytosis, and skin appendageal routes, while improving transfection, immune evasion, and homotypic targeting. Studies demonstrated efficient silencing of TGF‐β1, suppression of α‐SMA, ECM deposition, and pro‐inflammatory cytokine release, thereby mitigating fibrotic progression. The system provides an innovative strategy for transdermal targeted siRNA therapy of HS and other skin diseases requiring genetic intervention.

## Graphical abstract

Hypertrophic scar fibroblasts membrane‐hybridized cationic transfersomes enable efficient loading and transdermal delivery of TGF‐β1 siRNA through multiple skin penetration pathways. The biomimetic platform enhances homotypic targeting, transfection, and immune evasion, suppresses TGF‐β1‐driven fibrotic signaling, and reduces extracellular matrix deposition, providing a targeted noninvasive strategy for hypertrophic scar gene therapy.

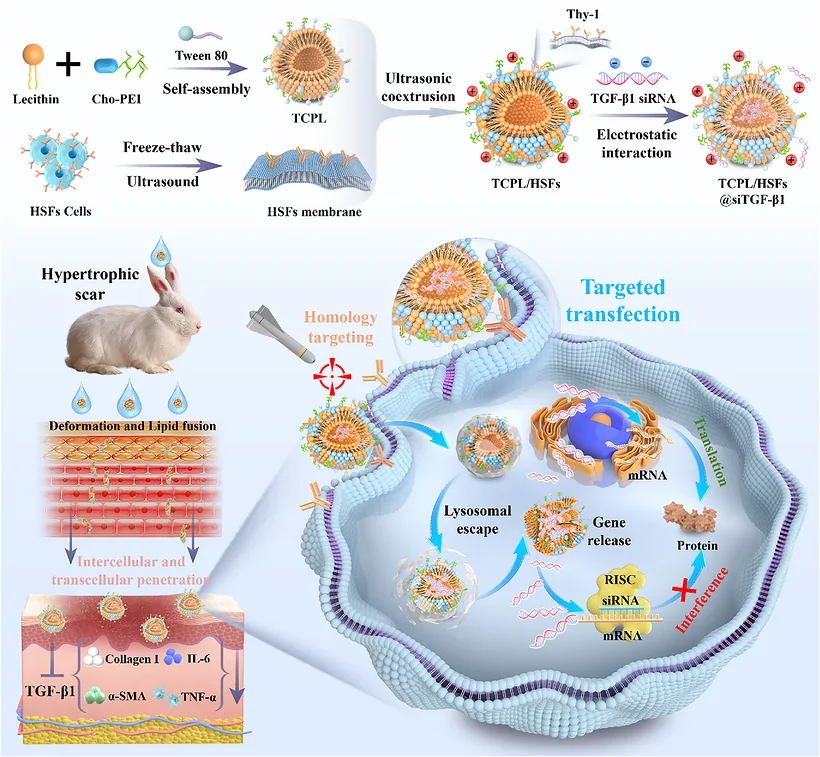

## 1 Introduction

Hypertrophic scars (HS) are recurrent and inflammatory dermal fibrotic disorders that arise following skin injury, clinically characterized by abnormal fibroblast proliferation and excessive extracellular matrix (ECM) deposition, leading to a high recurrence rate as well as pain, pruritus, and functional impairment, which markedly reduce patients’ quality of life [1, 2]. Over decades of clinical practice and research, a variety of therapeutic modalities have been developed, including local compression therapy, corticosteroid injections, laser therapy, cryotherapy, surgical excision, and radiotherapy [3]. However, these conventional treatments are often compromised by inconsistent efficacy and poor patient compliance, which continue to hinder clinical management and underscore the urgent need for safer, more effective, and durable therapeutic strategies [4]. Studies have identified TGF‐β1 as a key driver of hypertrophic scar fibrosis. As a highly conserved cytokine, it is widely regarded as the most representative and functionally important member of the TGF‐β family. By activating both canonical (Smad‐dependent) and non‐canonical (Smad‐independent) signaling pathways, TGF‐β1 induces fibrosis, thereby promoting myofibroblast activation, ECM production, and suppression of matrix degradation [5]. In recent years, considerable efforts have been devoted to modulating the TGF‐β pathway to attenuate scar fibrosis. For instance, Li et al. developed an injectable hydrogel composed of anti‐scar peptides targeting TGF‐β. The released peptides inhibited the TGF‐β/Smad signaling cascade in macrophages and fibroblasts, thereby suppressing fibroblast activation through TGF‐β adsorption and potentially enabling scarless wound healing [6].

Currently, small interfering RNA (siRNA)‐based therapeutics leveraging RNA interference (RNAi) have shown unique advantages in HS treatment. Through complete complementary pairing with target mRNA, siRNAs can achieve gene silencing efficiencies exceeding 90%, rendering pathogenic genes promising therapeutic targets [7]. Systematic analysis of existing studies reveals that, despite substantial evidence of siRNA efficacy in both in vitro and in vivo scar models, clinical translation remains limited by several technical challenges [8]. First, siRNA therapy has certain pharmacological limitations, such as poor stability, rapid degradation, off‐target effects, and immune responses. Second, there are still critical knowledge gaps in the molecular mechanisms of siRNA transdermal penetration, particularly regarding the effects of the dense extracellular matrix, the barrier function of the stratum corneum, and the abnormal distribution of microvessels in HS tissue on siRNA delivery. Most importantly, existing studies generally lack cell‐specific delivery strategies, and the inability to achieve precise fibroblast targeting compromises therapeutic efficiency and raises the risk of off‐target effects.

In recent years, with the continuous advancement of research, a variety of technologies have been explored to improve the transdermal or localized delivery of siRNA, including liposomes, cell‐penetrating peptides, spherical nucleic acids, microneedles, and iontophoresis platforms. These strategies share the common goal of enhancing siRNA stability, skin penetration, and cellular uptake while minimizing systemic exposure [9]. Among delivery strategies, noninvasive lipid‐based systems have emerged as particularly attractive for siRNA administration. Unlike injectable or oral routes, they bypass first‐pass metabolism, provide stable delivery, exhibit strong skin‐targeting potential, and, most importantly, enable convenient and patient‐friendly administration [10, 11]. For example, Lei et al. reported a dual tissue‐ and cell‐penetrating liposomal platform composed of arginine‐based dendritic lipopeptides that mimic critical amino acid motifs within viral protein transduction domains and adopt virus‐like nanostructures. This system overcame the stratum corneum barrier, promoted fibroblast apoptosis, and facilitated collagen remodeling in thickened dermis, ultimately flattening hypertrophic scars [12]. Nonetheless, liposomal siRNA delivery remains broad‐spectrum in nature and fails to achieve effective targeting of pathogenic scar fibroblasts [9]. Nanocarriers based on cell membrane–biomimetic platforms have attracted increasing attention as next‐generation delivery systems, as they can integrate the structural advantages of nanocarriers with the biological functions of natural membranes, thereby enhancing biocompatibility, immune evasion, and potentially enabling homotypic targeting [13]. For example, Chen et al. engineered a biomimetic hybrid nanocarrier (CCMpHD) by fusing cancer cell membranes (CCMs) with pH‐sensitive liposomes. The presence of CCMs conferred homotypic targeting, while the pH‐responsive liposomes enabled efficient internalization through membrane fusion. Both in vitro and in vivo studies confirmed significantly enhanced cellular uptake, thereby improving delivery efficiency and providing a versatile strategy for the transport of diverse therapeutics [14]. In addition, increasing attention has been directed toward barrier‐adaptive delivery strategies for the treatment of pathological scars, including microneedle‐based systems, iontophoresis‐assisted patches, and biomaterial‐enabled therapeutic platforms. This trend highlights the urgent need for delivery systems capable of penetrating scar tissue while selectively modulating pathogenic fibroblasts [15].

**SCHEME 1:**
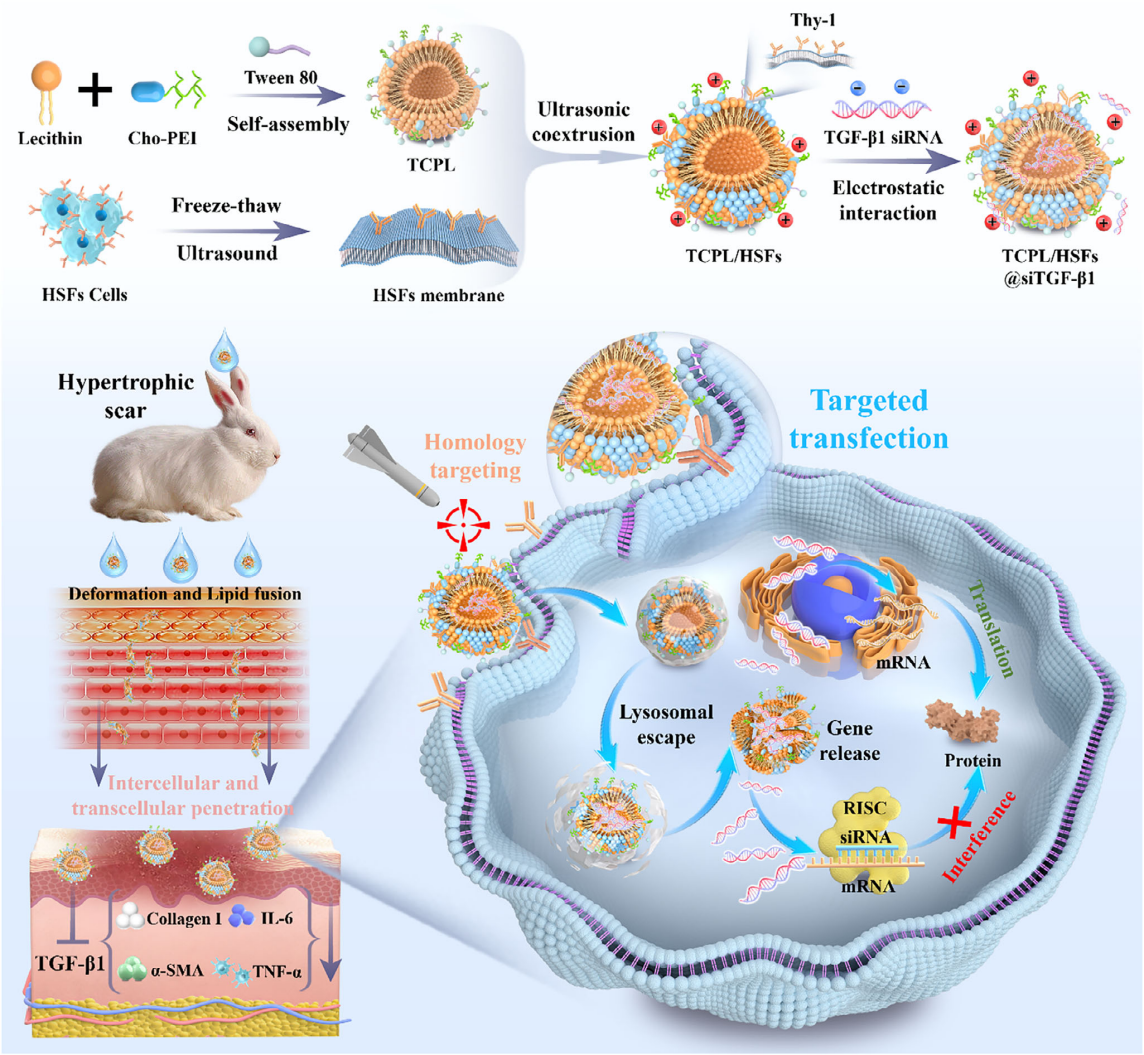
Schematic illustration of the construction of biomimetic hybrid transfersomes and targeted‐penetrating and transfection mechanism for the treatment of hypertrophic scar.

Given the therapeutic potential of TGF‐β1 siRNA (siTGF‐β1) in inhibiting fibrotic cascades by modulating the canonical TGF‐β/Smad pathway, and in light of the limitations of current siRNA carriers in terms of penetration, transfection, and targeting efficiency, we conducted a detailed proteomic analysis of primary human hypertrophic scar fibroblasts (HSFs). Based on these findings, we designed a novel delivery system by conjugating low‐molecular‐weight polyethyleneimine (PEI) to cholesteryl chloroformate (Cho) via amidation to form a hydrophobic backbone. This structure was subsequently co‐assembled with lecithin and incorporated with Tween 80 as a membrane fluidity regulator. Through reverse‐phase evaporation, high‐shear dispersion, and microjet technology, we optimized the composition to obtain a stable, ultradeformable cationic carrier (TCPL). The TCPL was then hybridized with primary HSFs membranes via ultrasonic co‐extrusion to endow the transfersomes system with active homotypic targeting, followed by electrostatic self‐assembly to efficiently load siTGF‐β1. We systematically elucidated the multi‐level mechanisms of the system, including transdermal penetration, intracellular release, and signaling regulation, with particular emphasis on the inhibition of TGF‐β signaling and its remodeling of the fibrotic microenvironment. Compared with conventional delivery systems, this platform not only enhances siRNA loading, transdermal penetration, and intracellular delivery, but also endows the siRNA with biomimetic immune evasion capability and active homotypic targeting toward HSFs, thereby providing an innovative delivery strategy for root‐cause treatment of pathological scarring (Scheme 1). Beyond HS therapy, the biomimetic transfersomes system can be extended to other immune‐related skin disorders requiring noninvasive targeted gene interventions, highlighting its significant potential for clinical translation.

## 2 Materials and Methods

### 2.1 Materials and Reagents Preparation

Proteomic analysis was performed by Kidio Biotechnology Co., Ltd. (Guangzhou, China) using Tims TOF Pro2/Orbitrap Exploris 480. Soybean lecithin and Tween 80 were purchased from Aladdin Biochemical Technology Co., Ltd. (Shanghai, China). Polyethyleneimine (PEI, Mn = 1800) was obtained from Macklin Biochemical Co., Ltd. (Shanghai, China). Cholesteryl chloroformate (Cho) was purchased from Qiyun Biotechnology Co., Ltd. (Guangzhou, China). pcDNA3.1‐GFP and pcDNA3.1‐RFP plasmids were purchased from GenePharma (China). The BCA protein assay kit was purchased from ABKBio (Xiamen, China). Dio staining kits were obtained from KeyGEN Biotech Co., Ltd. (Nanjing, China). β‐actin antibody (RRID: AB_3083699), TGF‐β1 antibody (RRID: AB_3753412), α‐SMA antibody (RRID: AB_2910228), collagen I antibody (RRID: AB_3676315) and ZO‐1 antibody (RRID: AB_3751487) were obtained from Servicebio. Human keratinocytes (HaCaT, RRID: CVCL_0038), human monocytic leukemia (THP‐1, RRID: CVCL_0006), normal human dermal fibroblasts (HDF) and human hypertrophic scar fibroblasts (HSFs) were purchased from Meisen Cell Technology Co., Ltd. (Zhejiang, China).

### 2.2 Differential proteomic analysis of HDF and HSFs

#### 2.2.1 Cellular protein extraction and Enzymatic Digestion

For proteomic analysis, normal human dermal fibroblasts (HDF) and human hypertrophic scar fibroblasts (HSFs) were cultured independently until approximately 80% confluence. The cells were washed three times with ice‐cold PBS and harvested using trypsinization. Cell suspensions were centrifuged at 4 °C, and the resulting pellets were washed twice with ice‐cold PBS. Three independently cultured cell samples were prepared for each group. The cell pellets were immediately frozen in liquid nitrogen and stored at −80 °C until proteomic analysis. A lysis buffer containing 8 M urea and 1% SDS was prepared. Immediately before use, a protease inhibitor was added to the lysis buffer at a ratio of 1:100, followed by vortex mixing and centrifugation. The prepared lysis buffer was then kept on ice until use. Cell pellet samples of HDF and HSFs were removed from storage at −80 °C. An appropriate volume of lysis buffer was added to each sample, followed by vortexing until the cell pellets were completely resuspended. The samples were then lysed on ice for 30 min. When the lysates became viscous, they were subjected to sonication on ice for 2 min using an ultrasonic cell disruptor. Subsequently, the samples were centrifuged at low temperature for 15 min, and the supernatants were collected. Sample preparation included protein denaturation, reduction, alkylation, enzymatic digestion, and peptide desalting. The eluted peptides were vacuum‐dried and stored at −80 °C until further analysis.

#### 2.2.2 DIA Data Acquisition and Analysis

The desalted and lyophilized peptides were reconstituted and analyzed by liquid chromatography–tandem mass spectrometry. The analytical platform consisted of a timsTOF Pro 2 mass spectrometer coupled to an UltiMate 3000 liquid chromatography system. A total of 200 ng of peptides from each sample was loaded and separated using a 60‐min gradient at a column temperature of 50 °C. Data‐independent acquisition was performed in diaPASEF mode over an *m/z* range of 349–1229, with an isolation window width of 40 Da. During PASEF MS/MS acquisition, the collision energy was varied linearly with ion mobility from 59 to 20 eV. The DIA data were analyzed using Spectronaut 18 with the default settings. Protein identification criteria were set at a precursor‐level false discovery rate (FDR) of 1.0% and a protein‐level FDR of 1.0%. The decoy database was generated using the mutated strategy. Automatic calibration was performed by Spectronaut, and the data were normalized using a local normalization strategy. Peptides meeting an FDR threshold of less than 1.0% were used for protein‐group quantification using the MaxLFQ algorithm.

### 2.3 Preparation and Characterization of TCPL/HSFs

#### 2.3.1 Preparation of TCPL

Cho‐PEI was synthesized as previously described [16]. Polyethyleneimine (PEI) was dissolved in anhydrous dichloromethane under ice‐bath conditions with vigorous stirring. A dichloromethane solution of cholesteryl chloroformate was then added dropwise in the presence of triethylamine as a catalyst. The reaction mixture was maintained at low temperature for 4 h, followed by further reaction at room temperature for 3 h. Afterward, the organic solvent was removed by rotary evaporation, and the resulting product was redissolved in 0.1 M hydrochloric acid solution. The redissolved product was extracted twice with 100 mL dichloromethane and subsequently washed with acetone to induce precipitation. The final product was obtained as a white Cho‐PEI powder after vacuum drying for 24 h, with a yield of 70%. The structure of Cho‐PEI was characterized by proton nuclear magnetic resonance (^1^H NMR) spectroscopy and Fourier transform infrared (FTIR) spectroscopy. 1H NMR was used to confirm the successful conjugation of cholesterol moieties to PEI, while FTIR was employed to further verify changes in the characteristic functional groups. For TCPL preparation, soybean lecithin (0.1%, w/v) and Cho‐PEI (0.1%, w/v) were dissolved in 10 mL of an ethanol/chloroform mixture (1:1, v/v) until completely solubilized, followed by the addition of Tween 80 (0.1%, w/v). The mixture was subsequently processed by high‐speed homogenization, rotary evaporation, and high‐pressure microjet treatment to obtain a TCPL suspension. TCL was prepared in the same manner, with cholesteryl chloroformate (Cho) used in place of Cho‐PEI. Similarly, CPL was obtained by dissolving soybean lecithin (0.1%, w/v) and Cho‐PEI (0.1%, w/v) in 10 mL of ethanol/chloroform (1:1, v/v), followed by sequential high‐speed homogenization, rotary evaporation, and high‐pressure microjet processing to yield a CPL suspension.

#### 2.3.2 Extraction of Hypertrophic Scar Fibroblast Cell Membranes

Hypertrophic scar fibroblast cell membranes were extracted from human and rabbit scar fibroblasts following previously reported protocols [17]. Briefly, cells were expanded in 15 cm culture dishes until about 80% confluence, after which they were harvested using a cell scraper. The collected cell pellets were resuspended in membrane protein extraction buffer and subjected to three cycles of freezing and thawing. Subsequently, the cells were sonicated for 10 min using a probe sonicator, and the supernatant was collected by high‐speed centrifugation. The precipitate, representing the isolated scar fibroblast cell membranes, was stored at −80 °C for further use. To preserve the integrity and activity of membrane proteins, all procedures were carried out on ice. For in vitro studies, cell membranes were extracted from primary human hypertrophic scar fibroblasts (HSFs) for mechanistic, uptake, and transfection‐related experiments. For in vivo therapeutic studies in the rabbit ear hypertrophic scar model, cell membranes were extracted from rabbit scar fibroblasts to maintain species consistency.

#### 2.3.3 Quantification of Membrane Proteins

The protein concentration of extracted cell membranes was determined using a BCA protein assay kit [18]. Briefly, protein standards with different concentrations (0.1, 0.15, 0.2, 0.3, 0.4, and 0.5 mg/mL) were prepared and used to generate a standard calibration curve based on absorbance measured at 562 nm. The isolated cell membrane samples were diluted fivefold with nuclease‐free water, and their absorbance was measured in 96‐well plates to calculate protein concentrations according to the standard curve.

#### 2.3.4 Preparation of TCPL/HSFs

The preparation of TCPL/HSFs was performed with modifications based on previously reported methods [14, 19]. To ensure membrane protein activity and fusion efficiency, ultrasonic co‐extrusion was employed to hybridize HSFs membranes with TCPL. Specifically, 1 mL of TCPL suspension was mixed with HSFs membrane preparations corresponding to 2.7, 5.4, or 10.8 µg membrane protein (5, 10, or 20 µL of membrane suspension at 0.54 mg/mL), followed by ultrasonic incubation and extrusion, followed by ultrasonic incubation (360 W, 40 kHz) for 2 min and subsequent incubation at room temperature for 10 min.

#### 2.3.5 Confocal Co‐Localization Analysis

The fusion and co‐localization of TCPL and HSFs membranes were verified using a confocal laser scanning microscope (LSM880, Zeiss, Germany). HSFs membranes were labeled with Dio (green), and TCPL was labeled with the red fluorescent dye Dil. After hybridization by ultrasonic co‐extrusion as described above, the resulting fluorescently labeled TCPL/HSFs were observed and imaged under the LSM880 with excitation wavelengths set at 484 and 549 nm [19].

#### 2.3.6 Fluorescence Resonance Energy Transfer (FRET) Assay

Membrane fusion efficiency was further assessed using a FRET assay as previously reported [20]. Briefly, TCPL (1 mL) was co‐labeled with Dil and Dio, followed by the addition of different volumes of HSFs membranes (20, 10, and 5 µL) for ultrasonic co‐extrusion fusion. Fluorescence spectra were recorded using a fluorescence spectrophotometer at an excitation wavelength of 470 nm, and emission was collected between 490 and 600 nm. With increasing HSFs membrane content, the distance between fluorescent dyes increased due to hybridization, resulting in enhanced Dio fluorescence and decreased Dil fluorescence intensity.

#### 2.3.7 Serum Stability Assay

For serum stability evaluation, TCPL (1 mL) was labeled with Dil and Dio and subsequently hybridized with HSFs membranes (10 µL) by ultrasonic co‐extrusion. The fluorescently labeled TCPL/HSFs were incubated in 10% FBS at 37°C for 4, 12, and 24 h. Acetonitrile was then added to disrupt the structures.

### 2.4 Preparation and Characterization of TCPL/HSFs@siTGF‐β1

#### 2.4.1 Preparation of TCPL/HSFs@siTGF‐β1

At room temperature, siTGF‐β1 solution was added to TCPL/HSFs suspension at a mass ratio (w/w) of 1, 5, 10, 20, 50, 100. The mixture was vortexed at 1000 rpm for 1 min and incubated for 30 min to obtain the TCPL/HSFs@siTGF‐β1 complex. The hydrodynamic diameter, polydispersity index (PDI), and zeta potential of the complexes were measured by dynamic light scattering using a Malvern Zetasizer. For morphological characterization, a drop of TCPL/HSFs@siTGF‐β1 solution was placed on a carbon‐coated copper grid, negatively stained with phosphotungstic acid, air‐dried, and observed under transmission electron microscopy (TEM) coupled with energy‐dispersive spectroscopy (EDS). High‐resolution transmission electron microscopy (HRTEM) was further performed to examine the detailed vesicular morphology and membrane‐like structural features.

#### 2.4.2 Agarose Gel Electrophoresis

The loading efficiency of siTGF‐β1 was evaluated by agarose gel electrophoresis. Briefly, 0.7 g of agarose was dissolved in 70 mL TAE buffer, boiled, and supplemented with 2 µL GoldView nucleic acid dye before casting into a gel tray. After solidification for 30 min, 2 µL of loading buffer was added to each sample (TCPL/HSFs@siTGF‐β1, TCPL/HSFs, or siTGF‐β1), followed by electrophoresis at 120 V for 25 min. The gels were visualized using a UV imaging system at 365 nm to confirm siRNA loading [21].

#### 2.4.3 SDS‐PAGE

The membrane proteins in TCPL/HSFs@siTGF‐β1 complexes were characterized by SDS‐PAGE. Gel solutions of appropriate concentrations were prepared according to the kit instructions. Protein samples were mixed with loading buffer and denatured by boiling at 90°C for 5 min. Electrophoresis was conducted in MOPS–SDS buffer at a constant voltage of 80 V until the samples entered the separating gel, then switched to 120 V until the bromophenol blue dye front reached the bottom. Following electrophoresis, gels were removed, stained with Coomassie Brilliant Blue, and destained multiple times before analysis [22].

#### 2.4.4 Assessment of siRNA Protection from Degradation

To assess serum stability, TCPL/HSFs@siTGF‐β1 complexes (w/w = 50) and siTGF‐β1 solution were incubated with 10% FBS (v/v) at 37 °C for 0, 6, and 24 h, followed by agarose gel electrophoresis. To assess nuclease resistance, TCPL/HSFs@siTGF‐β1 and siTGF‐β1 were incubated with RNase A (50 U/mL) at 37 °C for 4, 8, and 24 h, and degradation was analyzed by agarose gel electrophoresis [23].

#### 2.4.5 Deformability of TCPL

1 mL of CPL, TCPL, or TCPL/HSFs were separately added to a liposome extruder. The liposomes were extruded through a 50 nm polycarbonate membrane at a pressure of 1 MPa. The extruded volume was recorded, and the particle size before and after extrusion was measured using a Malvern laser particle size analyzer [24].

$$\begin{array}{cc} & \mathrm{Deformation}\;\mathrm{Index}\\ & =\left(\mathrm{Volume}\;\mathrm{through}\;50\;\mathrm{nm}\;\mathrm{polycarbonate}\;\mathrm{film}\right)\\ & \times {\left(\frac{\mathrm{Particle}\;\mathrm{size}\;\mathrm{after}\;\mathrm{extrusion}}{\mathrm{Polycarbonate}\;\mathrm{membrane}\;\mathrm{pore}\;\mathrm{size}}\right)}^{2} \end{array}$$

### 2.5 Homologous Active Targeting Ability Assay

#### 2.5.1 Cellular Co‐Localization Analysis

Cells were incubated with TCPL/HSFs@Cy5‐siRNA (siRNA = 1 µg/well) for 24 h, followed by PBS washing. Membranes were stained with Dio for 15 min, fixed with electron microscopy fixative, washed thoroughly, and nuclei were counterstained with DAPI. After final PBS washing, samples were placed on ice and immediately imaged using a confocal laser scanning microscope at excitation wavelengths of 405, 488, and 649 nm [25, 26].

#### 2.5.2 Homotypic Uptake Assay

To assess homotypic uptake, HaCaT, HDF, HSFs and THP‐1 cells were seeded into 12‐well plates (1×10^5^ cells/well) and cultured for 12 h until adhesion. Cells were treated with TCPL/HSFs@Cy5‐siRNA (siRNA = 1 µg/well) for 24 h, then washed with PBS, harvested by trypsinization (100 µL/well), and analyzed by flow cytometry [27].

#### 2.5.3 Homotypic Competition Assay

In the pretreatment group, 10 µL of extracted HSFs membrane solution was added and incubated for an additional 12 h. After removal of the medium, cells were treated with TCPL/HSFs@Cy5‐siRNA (siRNA = 1 µg/well) for 24 h. Cells were then washed with PBS, harvested by trypsinization and analyzed by flow cytometry.

#### 2.5.4 Lysosomal Escape Study

Cells were incubated with TCPL/HSFs@siRNA complexes for 8 h, washed with PBS, and stained with LysoTracker Green for 1 h. Cells were then washed, fixed with paraformaldehyde, and nuclei were stained with DAPI. After final PBS washing, samples were placed on ice and immediately imaged using a confocal laser scanning microscope.

### 2.6 In Vitro and In Vivo Transdermal Permeability Assays

#### 2.6.1 In Vitro Transwell Penetration Assay

A Transwell system containing HaCaT was established to simulate the in vitro skin penetration model [28]. Briefly, HaCaT were seeded into the upper chamber of a Transwell insert and cultured until complete adhesion. In parallel, 1×10^5^ HSFs were seeded into the lower chamber for attachment. TCPL/HSFs@Cy5‐siRNA (siRNA = 1 µg/well) was added to the upper chamber and incubated for 24 h. Cells in the lower chamber were harvested by trypsinization, kept on ice, and analyzed by flow cytometry.

#### 2.6.2 Extracellular Matrix (ECM)‐Mimicking Penetration Assay

To simulate the dense ECM of hypertrophic scars, Matrigel was thawed at room temperature and mixed homogeneously with HSFs harvested from T25 flasks (1 mL Matrigel). The mixture was plated into confocal culture dishes and incubated at 37 °C for 15 min to allow gel formation. Complete medium containing Cy5‐siRNA, TCPL@Cy5‐siRNA, or TCPL/HSFs@Cy5‐siRNA (siRNA = 1 µg/well) was then added. After 24 h of incubation, the distribution of siRNA within the Matrigel was analyzed using 3D z‐stack confocal scanning (LSM880, Zeiss, Germany) [12].

#### 2.6.3 In vivo skin penetration assay

To evaluate in vivo penetration, mice were anesthetized, and hair on the dorsal skin was removed using a clipper followed by depilatory cream to eliminate background fluorescence interference. TCPL/HSFs@Cy5‐siRNA solution or Cy5‐siRNA solution was topically applied to the dorsal skin and allowed to absorb completely. After 24 h, dorsal skin tissues were excised, embedded, cryosectioned, and stained with DAPI. The distribution of Cy5‐siRNA penetration was visualized using confocal laser scanning microscopy.

### 2.7 Transdermal Mechanism Research

#### 2.7.1 Transepithelial Electrical Resistance (TEER) Measurement

To investigate whether TCPL/HSFs@siTGF‐β1 modulates epithelial barrier integrity, a HaCaT monolayer Transwell model was established and transepithelial electrical resistance (TEER) was monitored. A Transwell system with a HaCaT monolayer (3 µm pore size, 24‐well format) was established [29]. Briefly, HaCaT were seeded in the upper chamber. TEER values were monitored daily using an epithelial voltohmmeter (EVOM). Once stable TEER values were reached, siTGF‐β1 or TCPL/HSFs@siTGF‐β1 (siTGF‐β1 = 1 µg/well) was added to the upper chamber and TEER values were re‐measured after 24 h. After treatment, the formulation‐containing medium was removed, and the monolayers were gently washed twice with PBS to eliminate residual formulation. Fresh complete medium was then added, and TEER values were re‐measured at 24 h after formulation removal.

#### 2.7.2 Transcellular Transport Pathway Analysis

HaCaT were treated with Cy5‐siRNA or TCPL/HSFs@Cy5‐siRNA (siRNA = 1 µg/well) and incubated for 12 h. After washing to remove residual materials, cell supernatants were collected at 24 h, and fluorescence intensity was measured using a fluorescence spectrophotometer to evaluate apical exocytosis. Culture medium supernatant from untreated cells served as the control. Each group was tested in triplicate [30].

#### 2.7.3 Skin Appendage Pathway Analysis

For evaluation of penetration through skin appendages, mice were anesthetized and dorsal hair was removed using a clipper and depilatory cream to minimize fluorescence interference. TCPL/HSFs@Cy5‐siRNA or free Cy5‐siRNA solution was topically applied to the dorsal skin and allowed to absorb completely. After 24 h, dorsal skin tissues were excised, embedded, cryosectioned, and immunostained with keratin 14 (Krt14) to label sweat glands and hair follicles in the epidermis and dermis. The penetration and distribution of Cy5‐siRNA were then visualized by confocal laser scanning microscopy.

### 2.8 Transfection Ability Assay

#### 2.8.1 Transfection of HSFs

HSFs were treated with TCPL/HSFs@GFP (pcDNA3.1‐GFP = 1 µg/well). PEI‐25000@GFP (w/w = 1.3:1) served as a positive control, while cells treated with naked GFP plasmid served as a negative control. Each group was tested in triplicate. After 24 h incubation, the culture medium was replaced with 1 mL of fresh complete medium, and cells were cultured for an additional 24 h. Cells were harvested by trypsinization and immediately analyzed by flow cytometry. GFP expression was further qualitatively confirmed by confocal laser scanning microscopy [31].

#### 2.8.2 In Vivo Transfection Assay

To exclude interference from hair, dorsal hair of mice was removed using clippers and depilatory cream. TCPL and CPL were complexed with plasmid pcDNA3.1‐RFP, and 100 µL of each lipoplex was topically applied to the dorsal skin once every 24 h until complete absorption. Naked RFP plasmid and untreated mice served as controls (n = 3). After 24 h, in vivo red fluorescence expression was detected using a small animal imaging system (excitation = 520 nm, emission = 570 nm).

#### 2.8.3 Cytotoxicity Assay

After adhesion, HSFs cells were treated with TCPL/HSFs@GFP or PEI‐25000@GFP (w/w = 1.3:1, pcDNA3.1‐GFP = 1 µg/well). Control cells were cultured in complete medium only. Each group was set up in triplicate. After 24 h of treatment, the medium was replaced with CCK‐8 reagent, incubated for 1 h, and absorbance was measured at 450 nm [32].

#### 2.8.4 Targeted Transfection Efficiency Assay

HaCaT, HSFs and HDF cells were seeded in 12‐well plates and cultured until reaching about 70% confluence. Cells were then treated with TCPL/HSFs@GFP (pcDNA3.1‐GFP = 1 µg/well). Each group was tested in triplicate. After 24 h incubation, the medium was replaced with fresh complete medium and cells were cultured for another 24 h.

### 2.9 siRNA Screening Assay

Four siRNA sequences targeting TGF‐β1 (TGFβ1‐963, TGFβ1‐1225, TGFβ1‐1713, and TGFβ1‐2007; sequences listed in Table S4) were synthesized. HSFs were seeded in 12‐well plates and cultured until about 70% confluence. Cells were then treated with TCPL/HSFs@siTGF‐β1 (siTGF‐β1 = 1 µg/well). Untreated cells served as controls. Each group was tested in triplicate. After 24 h incubation, the medium was replaced with 1 mL of fresh complete medium and cells were cultured for an additional 24 h. Cells were harvested by trypsinization, collected by centrifugation, and analyzed for TGF‐β1 expression at both the mRNA and protein levels. mRNA expression was quantified by qRT‐PCR, while protein expression was assessed by Western blot analysis.

### 2.10 In Vitro Anti‐Fibrosis Assay

#### 2.10.1 In Vitro Migration Assay

Briefly, HSFs were seeded in 6‐well plates and cultured for 12 h. Cells were then stimulated with TGF‐β1 protein (200 ng/mL) and incubated for 24 h. A straight scratch was made across the center of each well using a 200 µL pipette tip. Cells were subsequently treated with siTGF‐β1, TCPL/HSFs, or TCPL/HSFs@siTGF‐β1 (siTGF‐β1 = 1 µg/well). Cells cultured in medium alone served as controls. Images of wound closure were acquired at 12 h and 24 h using an inverted microscope, and the scratch width and migration area were quantified using ImageJ software [33].

#### 2.10.2 In Vitro Inhibition of Inflammation and Fibrosis

A Transwell co‐culture model of HaCaT and HSFs was established to simulate an in vitro scar treatment system. Briefly, HaCaT cells were seeded into the upper chamber of the Transwell system. Meanwhile, HSFs were seeded into the lower chamber for attachment. TCPL/HSFs@siTGF‐β1 (siTGF‐β1 = 1 µg/well) was added to the upper chamber and incubated for 24 h. HSFs in the lower chamber were harvested by trypsinization (100 µL). Protein expression levels of TGF‐β1, collagen I (Col I), and α‐SMA were analyzed by Western blot, while inflammatory cytokines (IL‐6 and TNF‐α) in the lower chamber supernatant were quantified using ELISA kits.

### 2.11 Animal Experiments

#### 2.11.1 Establishment of a Hypertrophic Scar Model

Healthy New Zealand white rabbits (2.5‐3.0 kg) were anesthetized with sodium pentobarbital (3%, 1 mL/kg) via marginal ear vein injection prior to surgery. Dorsal ear hair was removed using an electric clipper, followed by disinfection with povidone‐iodine. Two full‐thickness skin defects (10 mm in diameter, excluding cartilage) were created symmetrically on the dorsal surface of each ear using sterile skin biopsy punches, with at least 1 cm spacing between defects. The epidermis, dermis, and perichondrium were completely excised to ensure standardized and reproducible wounds [34]. To prevent contractile closure during the natural healing process, wounds were moistened daily with sterile saline, kept free from scab formation, and maintained in an open state. The New Zealand white rabbits, which will form hypertrophic scars after four weeks, are randomly grouped, with two animals per group and four scars per group. For topical treatment, 100 µL of the freshly prepared TCPL/HSFs@siTGF‐β1 aqueous transfersomal suspension was slowly and uniformly applied directly onto each rabbit ear scar and allowed to absorb completely. The 5‐FU concentration is 5%, and 100 µL of the solution is applied to each scar. Treatment is performed every two days for a duration of three weeks. For outcome assessment, the investigators responsible for ultrasound imaging, histological evaluation, and quantitative fluorescence analysis were blinded to group allocation [35].

#### 2.11.2 Scar Tissue Penetration

Hypertrophic scar‐bearing rabbits were anesthetized, and 100 µL of TCPL/HSFs@Cy5‐siRNA was gently applied to both scar sites and healthy skin areas until fully absorbed. After 24 h, scar tissues were excised, embedded, cryosectioned, and analyzed by confocal laser scanning microscopy to visualize penetration and distribution.

#### 2.11.3 Histological Evaluation of Skin Tissues

Scar tissues from each group were first examined by high‐frequency ultrasound imaging. Skin tissues (1 cm^2^) were then excised from the dorsal area, fixed in 4% paraformaldehyde, paraffin‐embedded, and sectioned into 5 µm slices. Sections were subjected to hematoxylin and eosin (H&E), Masson's trichrome, and Sirius Red staining.

#### 2.11.4 In Vivo Antifibrotic Analysis

Scar tissues collected after treatment were subjected to immunofluorescence analysis to detect Tunel‐positive apoptotic cells and the fibrotic biomarker α‐SMA. Stained sections were scanned using a fluorescence imaging system, and the proportion of immunopositive cells was quantified using ImageJ software.

### 2.12 Biocompatibility Test

#### 2.12.1 Biosafety Evaluation

C57BL/6 mice were administered TCPL/HSFs via tail vein injection with an injection volume of 100 µL per mouse, once per week for 4 consecutive weeks. Mice receiving normal saline served as controls. At the end of the study, major organs (Heart, Liver, Spleen, Lung, and Kidney) and serum were collected for further analysis. Organs were subjected to H&E staining to assess potential tissue toxicity. Alanine aminotransferase (ALT), aspartate aminotransferase (AST), blood urea nitrogen (BUN), and creatinine were measured using standard biochemical assay kits according to the instructions to evaluate hepatic and renal function.

#### 2.12.2 Immunogenicity Assessment

To evaluate the immunogenicity of TCPL/HSFs, serum was collected from treated and untreated mice. Serum samples were incubated with TCPL/HSFs as the primary antigen for 4 h, followed by incubation with fluorophore‐conjugated secondary antibodies against mouse IgM and IgG. Positive IgM or IgG signals indicated the production of specific antibodies against TCPL/HSFs in host circulation.

#### 2.12.3 Verification of Hepatic First‐Pass Effect

To verify the ability of the transdermal delivery system to avoid the hepatic first‐pass effect, 100 µL of TCPL/HSFs@Cy5‐siRNA lipoplexes were topically applied to the depilated dorsal skin of mice until fully absorbed. Control groups included untreated mice and mice receiving intravenous injection of Cy5‐siRNA formulations. After 24 h, in vivo fluorescence imaging was performed using a small animal imaging system to monitor siRNA biodistribution in major organs [36].

### 2.13 Statistical Analysis

Proteomic, cell‐based, and animal experiments were conducted using biological replicates. For physicochemical characterization, including particle size and potential measurements, repeated measurements performed on the same formulation batch were considered technical replicates. All data are presented as mean±standard deviation. Differences between different experimental groups were analyzed using the One‐way ANOVA method in GraphPad software (Inc., LA Jolla, CA, USA). The significance of differences was determined based on P values, where *P < 0.05; **P < 0.01; ***P < 0.001; ****P < 0.0001.

## 3 Results and Discussions

### 3.1 Proteomic Functional Analysis of Human Hypertrophic Scar Fibroblasts

**FIGURE 1:**
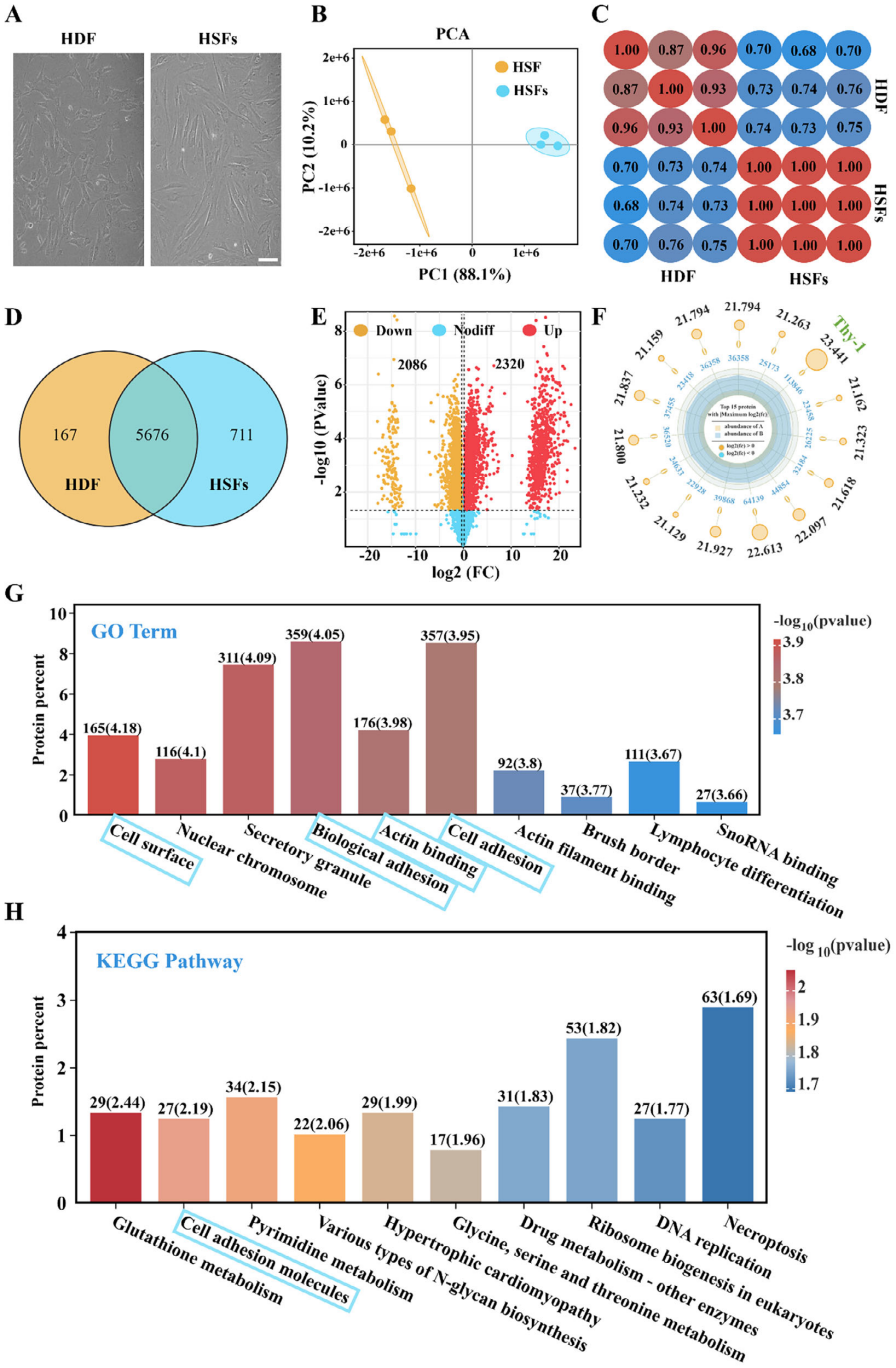
Proteomic analysis of HDF and HSFs. (A) Morphological images of HDF and HSFs (scale bar = 100 µm). (B) Principal component analysis (PCA) of protein expression profiles in HDF and HSFs. (C) Pearson's correlation analysis of proteomic data between HDF and HSFs. (D) Pie chart showing the number of differentially expressed proteins between HDF and HSFs. (E) Volcano plot of upregulated and downregulated proteins in HSFs. (F) Radar chart of the top 20 significantly differentially expressed proteins. (G) Gene Ontology (GO) enrichment analysis of HDF and HSFs. (H) KEGG pathway enrichment analysis of HDF and HSFs. n = 3, biological replicates.

To comprehensively investigate functional alterations in the proteome of human hypertrophic scar fibroblasts (HSFs), we performed systematic quantitative proteomic analysis of primary normal human dermal fibroblasts (HDF) and HSFs. Morphological evaluation revealed distinct differences between the two cell populations. Under bright‐field microscopy, normal skin fibroblasts exhibited a typical spindle‐shaped morphology with uniform size, orderly arrangement, and classical features of healthy dermal fibroblasts [37]. By contrast, HSFs displayed pronounced morphological heterogeneity, including enlarged cell bodies, irregular contours, and polygonal or flattened appearances. The cells were arranged in denser clusters, suggestive of enhanced adhesive properties (Figure 1A). Principal component analysis (PCA) clearly distinguished the two groups, indicating significant global differences in protein expression patterns (Figure 1B). Pearson's correlation analysis further confirmed strong intra‐group consistency and pronounced inter‐group differences, demonstrating both biological divergence and experimental reproducibility (Figure 1C). In total, 6,554 proteins were identified, among which 878 were differentially expressed between the groups, with 711 upregulated and 167 downregulated in HSFs (Figure 1D). A volcano plot provided an overview of these differential proteins and highlighted several functionally relevant candidates (Figure 1E). Of particular note, Thy‐1 expression was markedly elevated on the membrane surface of HSFs. As a glycosylphosphatidylinositol (GPI)‐anchored glycoprotein localized to the plasma membrane, Thy‐1 is widely implicated in cell adhesion, signal transduction, and immune modulation (Figure 1F) [38]. In addition to the enrichment of membrane‐associated adhesion‐related proteins, the proteomic analysis also identified several fibrosis‐relevant targets linked to the TGF‐β axis. Notably, TGFβ1 (ENSG00000105329) was significantly upregulated in hypertrophic scar fibroblasts compared with normal fibroblasts. Its protein abundance increasing from 42.8 to 1365.07 (log2FC = 4.9, FDR = 0.0003), indicating marked pathological activation of TGF‐β1‐related signaling in scar fibroblasts. We also observed marked upregulation of TGFβ1/1 (ENSG00000140682), a TGF‐β1‐induced transcript‐associated protein, and COL1A1 (ENSG00000108821), a canonical extracellular matrix component closely associated with fibrotic remodeling. These findings further support that the HSFs proteomic profile is characterized not only by enhanced membrane interaction features, but also by activation of a profibrotic molecular program related to TGF‐β signaling and extracellular matrix deposition. Gene Ontology (GO) enrichment analysis revealed that the differential proteins were significantly enriched in biological processes such as “cell surface,” “biological adhesion,” “actin binding,” and “cell adhesion” (Figure 1G)—processes consistent with the pathological features of scar tissue, including excessive ECM deposition and persistent tissue remodeling. Moreover, KEGG pathway enrichment analysis highlighted the upregulation of pathways associated with “cell adhesion molecules,” which are closely linked to fibroblast migration, adhesion, and survival, several of which are directly related to scar formation (Figure 1H). Thus, these proteomic results demonstrate that HSFs exhibit enhanced functional activity in cell–matrix and cell–cell interactions. The marked upregulation of Thy‐1 on the cell membrane not only underscores its critical role in scar fibrosis but also provides a promising molecular target for the rational design of actively targeted drug delivery systems. This discovery offers important mechanistic insights and theoretical support for the development of precision therapeutic strategies against hypertrophic scar.

### 3.2 Construction and Characterization of Biomimetic Hybrid Delivery Vehicles

**FIGURE 2:**
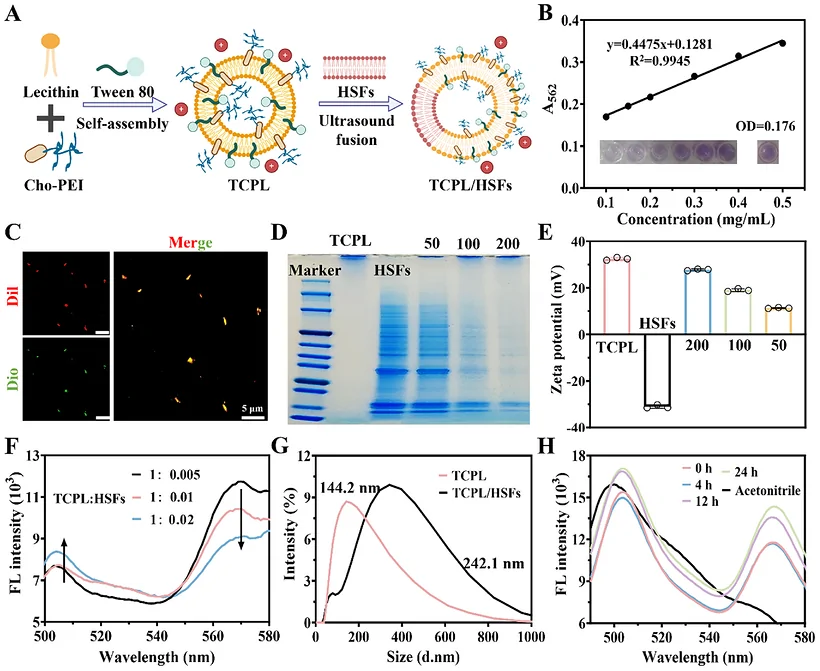
Synthesis and characterization of TCPL/HSFs. (A) Schematic illustration of the synthesis of TCPL/HSFs, schematic illustrations were created with BioRender.com. (B) Quantification of membrane protein content by BCA assay. (C) Confocal co‐localization analysis of TCPL/HSFs after hybridization. (D) SDS‐PAGE analysis of membrane proteins in TCPL/HSFs (v/v = 50, 100, 200). (E) Zeta potential changes of TCPL/HSFs after hybridization (v/v = 50, 100, 200, n = 3, technical replicates). (F) Fluorescence resonance energy transfer (FRET) analysis of TCPL/HSFs. (G) Particle size distribution of TCPL and TCPL/HSFs (n = 3, technical replicates). (H) Fluorescence intensity profiles of TCPL/HSFs incubated in 10% fetal bovine serum.

Based on proteomic results indicating significantly enhanced bioadhesion and binding functions of membrane proteins on hypertrophic scar fibroblasts (HSFs), we sought to construct a biomimetic hybrid delivery system to achieve noninvasive, targeted transdermal delivery to HSFs. First, low‐molecular‐weight polyethyleneimine (PEI) was grafted onto cholesteryl chloroformate (Cho) via esterification to obtain Cho‐PEI. A series of characterizations were performed on the prepared Cho‐PEI. As shown in Figure S1A, a characteristic allylic proton peak of Cho appeared at position *a*, an amide bond signal was observed at position *b*, and peaks corresponding to the amino and methylene groups of the PEI backbone and side chains were detected at position *c*. These findings preliminarily indicated the successful synthesis of Cho‐PEI. To confirm the chemical bond changes, Fourier transform infrared (FTIR) spectroscopy was employed to characterize its structure. As shown in Figure S1B, after the reaction between Cho and PEI, a stretching vibration peak of –CO–NH– appeared at approximately 1700 cm^−^^1^, demonstrating the conversion of the acyl chloride group into an amide group and further confirming the successful synthesis of Cho‐PEI. This conjugate, together with Tween 80, was subsequently incorporated into liposomal structures through high‐speed homogenization, reverse‐phase evaporation, and high‐pressure microjet techniques, generating a structurally stable carrier (TCPL). The extracted HSFs membranes were then hybridized with TCPL using ultrasonic co‐extrusion, forming a protein‐decorated hybrid system (TCPL/HSFs) (Figure 2A). Membranes were isolated from HSFs using a freeze–thaw ultrasonic extraction method to maximally preserve the conformation and activity of membrane proteins [39]. Protein quantification by BCA assay confirmed a consistent yield with a concentration of 0.54 mg/mL (Figure 2B), providing a solid basis for subsequent fusion. Confocal laser scanning microscopy further verified membrane fusion by fluorescence co‐localization. HSFs membranes were labeled with the lipophilic dye Dio, while TCPL was labeled with Dil. Strong yellow fluorescence in the merged images of TCPL/HSFs indicated pronounced colocalization of red and green signals, supporting successful membrane fusion between TCPL and HSFs membranes (Figure 2C). SDS‐PAGE analysis confirmed the retention of membrane proteins in the hybrid transfersomes, as the TCPL/HSFs group exhibited multiple protein bands corresponding to those of native HSFs membranes (Figure 2D). Since SDS‐PAGE mainly reflects the overall protein profile and cannot specifically confirm membrane identity, Western blot analysis was further performed. As shown in Figure S2, the membrane‐associated marker proteins Na^+^/K^+^‐ATPase and CD44 were detected in the extracted HSFs membrane fraction, further supporting the successful isolation of HSFs‐derived membrane components. TCPL and TCPL/HSFs were examined by high‐resolution transmission electron microscopy (HRTEM). As shown in Figure S3, both TCPL and TCPL/HSFs exhibited intact and approximately spherical vesicular morphologies. Compared with unhybridized TCPL, TCPL/HSFs showed an increased apparent particle size and a more distinct peripheral membrane‐like contour, while retaining the overall vesicular architecture of the artificial carrier. These morphological changes were consistent with the incorporation of HSFs‐derived membrane components into TCPL. Dynamic light scattering measurements revealed slight increases in particle size and decreases in zeta potential with higher membrane protein content, consistent with successful hybridization (Figure 2E,G). Three independently prepared batches of TCPL/HSFs were characterized to assess the consistency of cell membrane protein coating across different preparation batches. Western blotting showed comparable expression levels of the membrane‐associated proteins Na^+^/K^+^‐ATPase and CD44 among the three batches (Figure S4). In addition, particle size, PDI, and zeta potential showed no obvious interbatch differences (Table S1). These results indicate good batch‐to‐batch consistency in membrane protein retention and key physicochemical properties, supporting the reproducibility of the TCPL/HSFs preparation process. To validate membrane fusion from an energy transfer perspective, fluorescence resonance energy transfer (FRET) analysis was performed. The FRET spectra showed that with increasing HSFs membrane incorporation, donor fluorescence (Dio) intensity increased while acceptor fluorescence (Dil) decreased (Figure 2F), indicating an increased donor–acceptor distance and reduced FRET efficiency, thereby indirectly confirming successful membrane fusion [19]. Considering the effective incorporation of HSFs‐derived membrane components, the extent of membrane lipid mixing, and the need to retain sufficient positive surface charge to facilitate subsequent siRNA loading, we ultimately selected a hybridization ratio of 1 mL TCPL to 10 µL HSFs membrane suspension. This corresponds to a volume ratio of 100:1 and approximately 5.4 µg of membrane protein per milliliter of TCPL, and was used as the working formulation for subsequent experiments. Serum stability assays were conducted to evaluate the robustness of the hybrid transfersomes in physiological conditions. Fluorescently labeled TCPL/HSFs were incubated in 10% fetal bovine serum for 4, 12, and 24 h at 37 °C. The fluorescence intensity profiles showed no rapid structural disruption, suggesting excellent stability in serum (Figure 2H). Together, these results confirmed that TCPL/HSFs preserved membrane‐associated protein features and formed a stable biomimetic carrier suitable for subsequent siRNA loading.

### 3.3 Validation of siTGF‐β1 Loading

**FIGURE 3:**
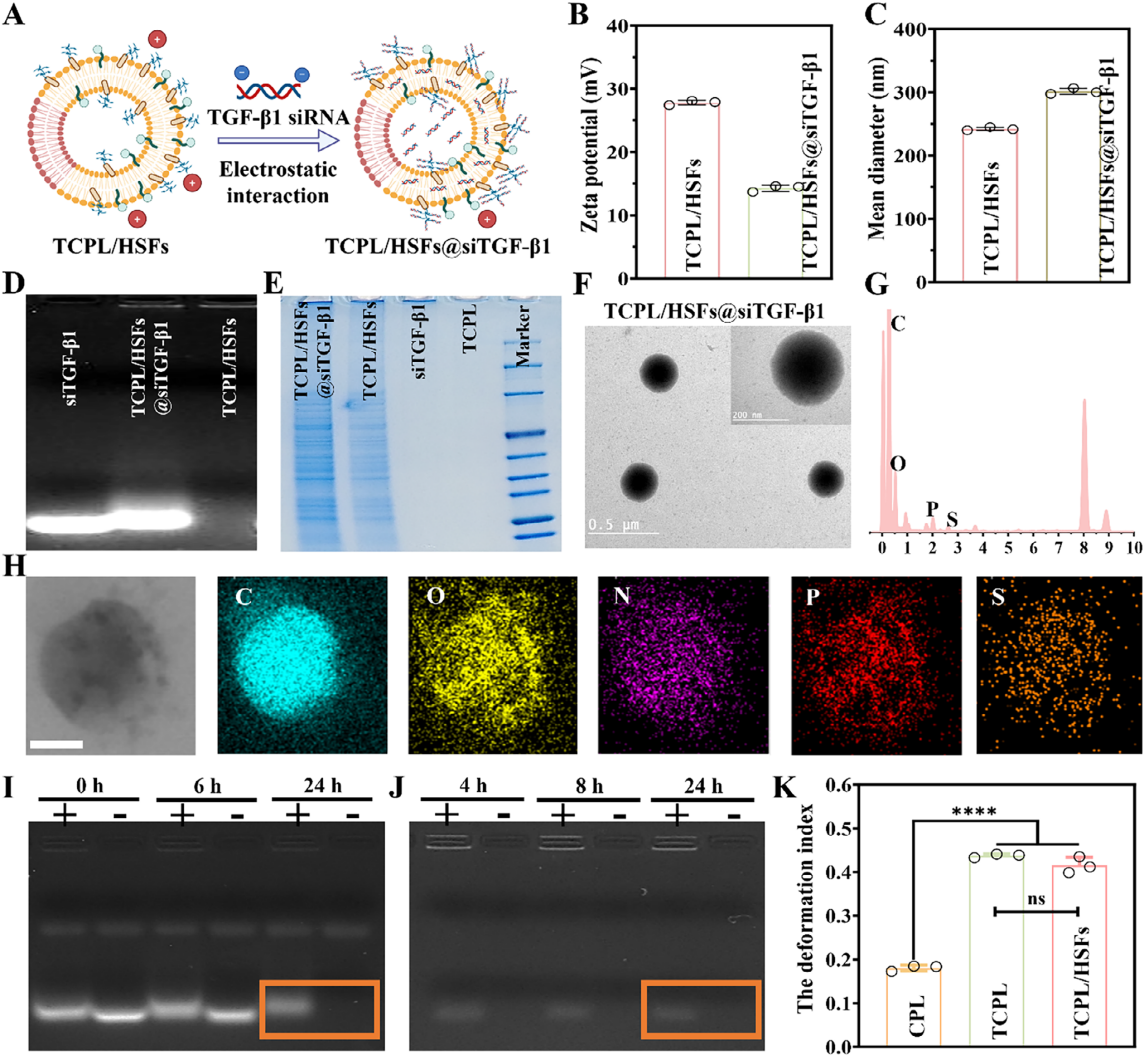
Preparation and characterization of TCPL/HSFs@siTGF‐β1. (A) Schematic illustration of TCPL/HSFs@siTGF‐β1 preparation, schematic illustrations were created with BioRender.com. (B) Zeta potential of carriers after siRNA loading (n = 3, technical replicates). (C) Particle size changes of carriers after siRNA loading (n = 3, technical replicates). (D) Agarose gel retardation assay. (E) SDS‐PAGE analysis of different groups. (F) TEM image of TCPL/HSFs@siTGF‐β1 (scale bar = 500 nm). (G–H) EDS qualitative and quantitative analysis of TCPL/HSFs@siTGF‐β1 (scale bar = 100 nm). (I) Serum stability assay. (J) RNase protection assay. “+” indicates the presence of the TCPL/HSFs carrier, while “−” indicates its absence. (K) Deformation index of different formulations. (n = 3, technical replicates). ****P < 0.0001.

To enable the effective delivery of siTGF‐β1, we constructed a gene delivery system by electrostatically assembling siRNA with the biomimetic hybrid transfersomes TCPL/HSFs, and systematically evaluated their loading capacity, structural features, and nucleic acid protection ability (Figure 3A). As shown in the Figure S5A, when the mass ratio of TCPL/HSFs@siTGF‐β1 exceeded 10, the complexes began to exhibit a positive zeta potential, indicating a stronger nucleic acid‐binding capacity that is favorable for preventing premature siRNA release. This was further well confirmed by the agarose gel electrophoresis assay (Figure S5B). Even when the mass ratio increased to 100, the zeta potential changed only slightly and remained stable at approximately +30 mV, suggesting good charge stability of the system over a relatively wide ratio range. Considering both carrier performance and experimental applicability, a mass ratio of 50 was selected for the subsequent studies. Dynamic light scattering analysis revealed that siTGF‐β1 loading induced a moderate decrease in potential and a slight increase in particle size, although the overall changes were minimal (Figure 3B,C). After membrane hybridization and siRNA loading, the PDI values remained below 0.2, indicating that the formulations maintained a relatively uniform size distribution (Table S2). During the 5‐day incubation period, TCPL/HSFs@siTGF‐β1 exhibited only minor changes in hydrodynamic diameter (294.3 ± 3.9 to 313.6±3.5 nm), while the PDI remained within 0.13–0.17 and the zeta potential remained stable at approximately 14.3–15.6 mV. No progressive increase in particle size or PDI was observed, indicating that siRNA‐loaded TCPL/HSFs maintained favorable stability during the observation period (Table S3). To quantify the siRNA loading capacity of TCPL/HSFs, nanoparticle tracking analysis (NTA) and fluorescence‐based quantification were performed. NTA showed that 100 µL of TCPL/HSFs contained approximately 2.11 × 10^10^ nanoparticles (Figure S6A). Cy5‐labeled siRNA was then used to determine the siRNA loading efficiency. After preparation of TCPL/HSFs@Cy5‐siRNA, the carrier‐associated siRNA was competitively displaced with heparin (2 mg/mL) at 65 °C for 10 min and subsequently separated from the nanoparticles by ultrafiltration. The amount of released Cy5‐siRNA was quantified according to the established fluorescence standard curve (Figure S6B). Of the initial 2 µg siRNA added to 100 µL of TCPL/HSFs, approximately 1.73 µg was associated with the nanoparticles, corresponding to a loading efficiency of 86.5%. Assuming a molecular weight of approximately 13.3 kDa for double‐stranded siRNA, 1.73 µg siRNA corresponds to approximately 7.83 × 10^1^ ^3^ siRNA molecules. Based on the particle concentration determined by NTA, each TCPL/HSFs particle was therefore estimated to carry an average of approximately 3.7×10^3^ siRNA molecules. To evaluate the batch‐to‐batch reproducibility of siRNA loading, three independently prepared batches of TCPL/HSFs were complexed with siRNA under identical conditions and analyzed by agarose gel electrophoresis. Quantitative analysis showed no significant difference in siRNA loading among the three batches (Figure S6C) and all three batches exhibited comparable electrophoretic migration and siRNA retention patterns, with no obvious interbatch differences (Figure S6D). Transmission electron microscopy (TEM) further confirmed that TCPL/HSFs maintained their well‐defined spherical morphology with uniform distribution after siRNA loading (Figure 3F). Energy‐dispersive spectroscopy (EDS) analysis detected sulfur signals in the TCPL/HSFs@siTGF‐β1 formulation, which provided supportive evidence for siRNA incorporation (Figure 3G,H). Agarose gel electrophoresis demonstrated efficient binding of siRNA within the complexes, as evidenced by distinct migration bands observed in TCPL/HSFs@siTGF‐β1 samples (Figure 3D). Consistently, SDS‐PAGE analysis showed multiple membrane protein bands in the complexes, confirming the structural integrity of HSFs‐derived membrane proteins, which may contribute to enhanced siRNA stability and targeting efficiency (Figure 3E) [40]. To assess siRNA stability under physiological conditions, the complexes were incubated in 10% FBS‐containing serum and RNase A solution. siTGF‐β1 solution was rapidly degraded in both conditions, whereas intact siRNA bands were detectable in TCPL/HSFs@siTGF‐β1 for up to 24 h, indicating robust protection of siRNA from nuclease‐mediated degradation (Figure 3I,J). Furthermore, an extrusion assay through 50 nm polycarbonate membranes was performed to evaluate carrier deformability. Compared with CPL, TCPL/HSFs exhibited a significantly higher deformation index, with no statistically significant difference from unhybridized TCPL, indicating superior deformability under pressure, which is favorable for transdermal transport through confined barrier structures (Figure 3K) [24]. These results demonstrate that the TCPL/HSFs@siTGF‐β1 complex not only achieves efficient siRNA loading but also preserves structural integrity, provides effective nuclease protection, and exhibits favorable deformability, underscoring its potential as a promising platform for transdermal nucleic acid delivery. For clarity, the key physicochemical characteristics of TCPL, TCPL/HSFs, and TCPL/HSFs@siTGF‐β1 are summarized in Table S2.

### 3.4 Validation of Target‐Silencing Efficiency of siTGF‐β1

**FIGURE 4:**
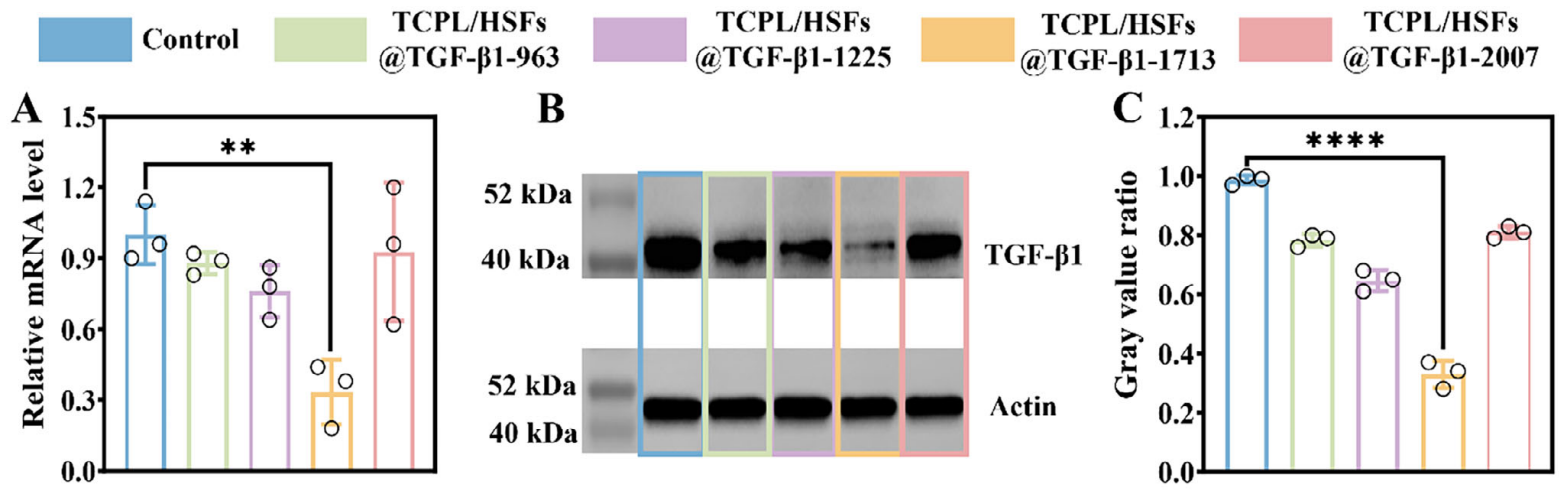
Validation of target‐silencing efficiency of siTGF‐β1. (A) TGF‐β1 mRNA expression levels in HSFs after transfection with TCPL/HSFs@siTGF‐β1. (B) Western blot analysis of TGF‐β1 protein expression in HSFs. (C) Densitometric quantification of TGF‐β1 protein expression in HSFs, (n = 3, biological replicates). **P < 0.01; ****P < 0.0001.

After constructing the delivery system, we screened for the siRNA sequence with the optimal silencing efficiency against TGF‐β1. Four siRNA sequences (TGFβ1‐963, TGFβ1‐1225, TGFβ1‐1713, and TGFβ1‐2007) were synthesized and individually loaded into TCPL/HSFs for transfection into HSFs. At 48 h post‐transfection, qRT‐PCR analysis revealed that all four siRNAs downregulated TGF‐β1 mRNA expression to varying degrees compared with the control group, among which TGFβ1‐1713 exhibited the strongest inhibitory effect, with the most significant reduction in mRNA levels (Figure 4A). Western blot analysis further confirmed the downregulation trend at the protein level, consistent with the qRT‐PCR results, with TGFβ1‐1713 showing the most pronounced suppression of TGF‐β1 protein expression (Figure 4B). Densitometric analysis quantitatively validated this observation, demonstrating that TGFβ1‐1713 exerted the greatest inhibitory effect at both transcriptional and translational levels (Figure 4C). A dose‐escalation experiment was further performed using siTGF‐β1 sequence 1713, which exhibited the highest silencing efficiency among the tested sequences, to determine whether increasing the dose could further enhance TGF‐β1 silencing. As shown in Figures S7A,B, increasing the treatment dose to twice the original dose (2×) further increased the TGF‐β1 knockdown efficiency to 81.4±0.2%. However, this enhanced silencing was accompanied by a reduction in cell viability to 81.9±1.5% (Figure S7C). These results indicate that increasing the dose can improve gene‐silencing efficiency to some extent but may compromise cellular compatibility at higher concentrations. Therefore, the original dose was selected for subsequent experiments as a balance between effective gene silencing and cell compatibility.

### 3.5 Evaluation of Homotypic Active Targeting Capability

**FIGURE 5:**
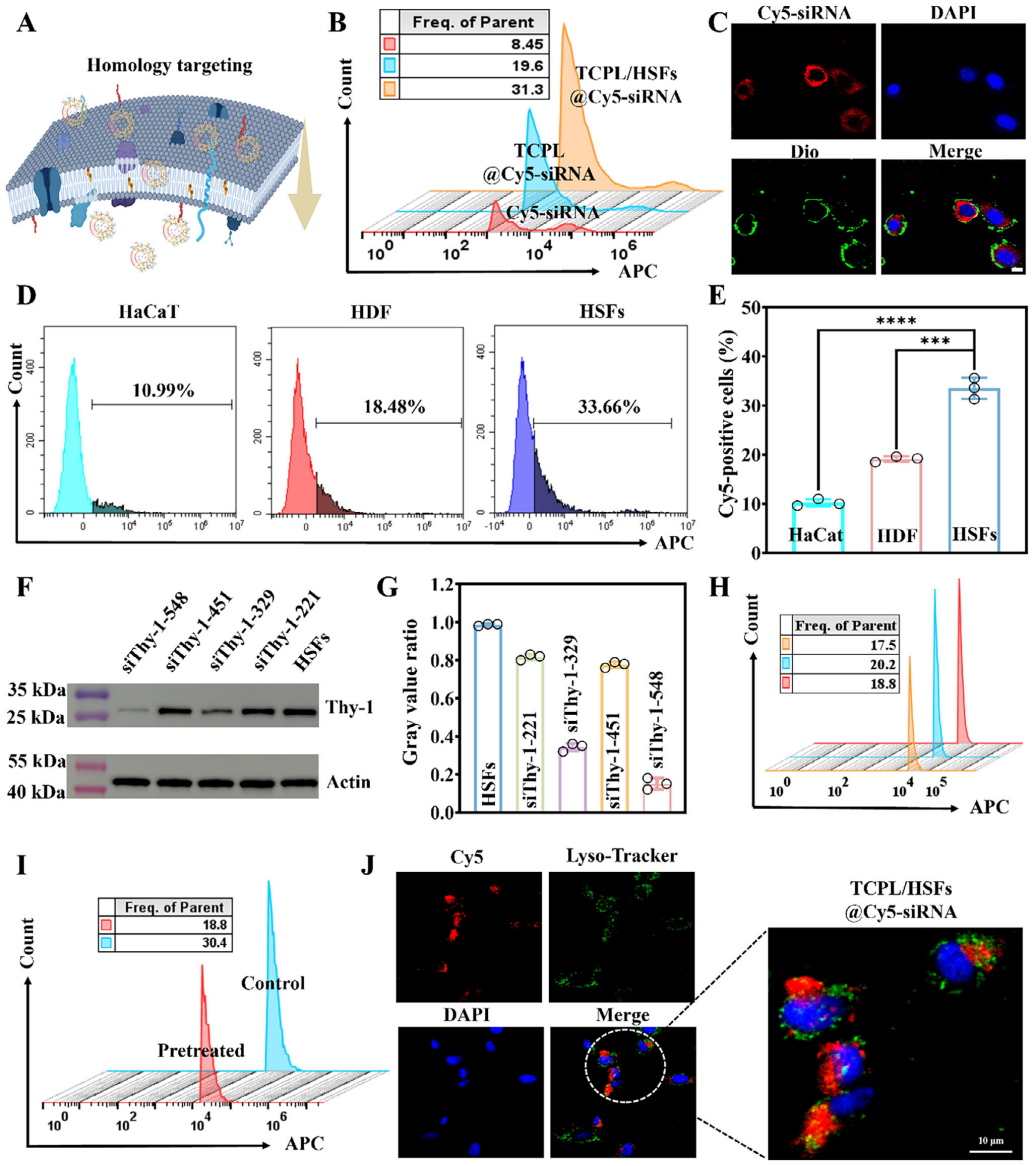
Validation of homotypic targeting effect. (A) Schematic illustration of membrane protein–mediated homotypic targeting and endocytosis, schematic illustrations were created with BioRender.com. (B) Quantitative analysis of TCPL/HSFs@Cy5‐siRNA uptake by HSFs, the percentages shown represent the proportion of Cy5‐positive cells among 10,000 analyzed cells in each group. Freq. of Parent represents the percentage of cells within the Cy5‐positive gate relative to the total number of cells in its immediately preceding parent gate. (C) Fluorescence co‐localization of TCPL/HSFs@Cy5‐siRNA uptake in HSFs. (D) Flow cytometric analysis and quantitative comparison of the uptake of TCPL/HSFs@Cy5‐siRNA by HaCaT, HDF and HSFs. (E)The percentages represent the proportions of Cy5‐positive cells among 10 000 analyzed cells in each group. (F) Western blot analysis of Thy‐1 expression in HSFs after transfection with different Thy‐1‐targeting siRNAs (siThy‐1‐221, siThy‐1‐329, siThy‐1‐451, and siThy‐1‐548). (G) Densitometric quantification of Thy‐1 protein expression. (H) Flow cytometric analysis of the cellular uptake of Cy5‐siRNA formulations coated with Thy‐1‐silenced HSFs membranes in recipient HSFs. (I) Quantitative uptake analysis of TCPL/HSFs@Cy5‐siRNA in HSFs pretreated with HSFs membranes. (J) Fluorescence images showing lysosomal escape of Cy5‐siRNA mediated by TCPL/HSFs (scale bar = 10 µm). n = 3, biological replicates, ***P < 0.001; ****P < 0.0001.

To validate the cellular uptake and homotypic targeting ability, a series of functional assays were performed (Figure 5A). As shown in Figure S8, to determine whether TCPL/HSFs@siTGF‐β1 could maintain their vesicular structure during transdermal penetration, the receptor solution was collected after 24 h of Franz diffusion cell. High‐resolution transmission electron microscopy (HRTEM) revealed intact vesicle‐like nanostructures with clearly defined membrane contours in the receptor compartment, indicating that at least a fraction of vesicles retained their structural integrity after crossing the skin barrier. Flow cytometry revealed that TCPL@Cy5‐siRNA produced a higher percentage of Cy5‐positive cells (19.6%) than free Cy5‐siRNA. Notably, TCPL/HSFs@Cy5‐siRNA resulted in a markedly higher percentage of Cy5‐positive HSFs (31.3%), suggesting that incorporation of HSFs‐derived membranes enhanced cellular recognition and promoted the association and internalization of the Cy5‐siRNA‐loaded vesicles (Figure 5B and Figure S9). Confocal laser scanning microscopy further confirmed this observation: the red fluorescence of Cy5‐siRNA clearly co‐localized with green Dio‐labeled cell membranes and blue DAPI‐stained nuclei, confirming that the complexes successfully were internalized (Figure 5C). To evaluate the effect of membrane homology on uptake, three cell types (HaCaT, HDF, and HSFs) were compared (Figure 5D). The percentage of Cy5‐positive cells was significantly higher in the TCPL/HSFs@Cy5‐siRNA treated HSFs group (33.66%) than in the HaCaT (13.48%) and normal HDF (10.99%) groups, supporting the homotypic targeting effect of the HSFs membrane‐hybridized vesicles [41]. Quantitative analysis further demonstrated the statistical significance of this difference (Figure 5E). Although the homotypic targeting efficiency of TCPL/HSFs toward HSFs was increased by less than twofold compared with HDF, proteomic analysis revealed that TGF‐β1 (ENSG00000105329) was markedly upregulated in HSFs, with its protein abundance increasing from 42.8 to 1365.07 (log2FC = 4.9, FDR = 0.0003). This pronounced differential expression indicates that TGF‐β1 is a pathologically enriched therapeutic target in HSFs. Therefore, siTGF‐β1 was selected to suppress the aberrantly elevated TGF‐β1 signaling in scar fibroblasts rather than to completely inhibit its physiological expression in normal tissues. In addition, the topical administration strategy used in this study spatially confines primarily to the scar region, thereby reducing exposure of the surrounding normal skin and systemic tissues. To verify the functional role of Thy‐1 in the homotypic targeting behavior of TCPL/HSFs, four siRNAs targeting Thy‐1 (siThy1‐221, siThy1‐329, siThy1‐451, and siThy1‐548) were designed and screened (Table S5). Western blot analysis showed that all four siRNAs reduced Thy‐1 protein expression in HSFs to different extents compared with the untreated control, among which siThy1‐548 achieved the most pronounced knockdown and was therefore selected for subsequent experiments (Figure 5F,G). Membranes derived from Thy‐1‐silenced HSFs were then used to construct TCPL/HSFs^siThy1^@Cy5‐siRNA, and their specific uptake by recipient HSFs was quantified by flow cytometry. Compared with the native HSFs membrane‐coated system, Thy‐1 knockdown led to a clear reduction in cellular uptake, indicating that elevated Thy‐1 expression contributes functionally to the homotypic recognition and uptake of TCPL/HSFs (Figure 5H). In homotypic competition experiments, pre‐incubation of cells with HSFs membranes markedly reduced the uptake of TCPL/HSFs@Cy5‐siRNA (Figure 5I and Figure S10), indicating that membrane protein–mediated recognition plays a key role in the active targeting capability of this system. As a GPI‐anchored membrane glycoprotein, Thy‐1 is closely associated with cell adhesion, membrane signaling, and intercellular interactions. Its elevated surface expression on HSFs may help mediate membrane protein‐driven homologous recognition and enhance material uptake, making it a potentially important mechanistic contributor. To confirm the intracellular delivery efficiency and lysosomal escape behavior, LysoTracker‐Green was used to label lysosomes, Cy5‐siRNA was labeled in red, and DAPI was used for nuclear staining (blue). Confocal imaging under the selected observation conditions showed reduced overlap between Cy5 fluorescence and lysosomal signals in the TCPL/HSFs@Cy5‐siRNA group compared with free siRNA, suggesting decreased lysosomal retention and intracellular behavior consistent with lysosomal escape. This interpretation is supported not only by the imaging result itself, but also by the enhanced uptake, transfection/gene‐silencing efficiency, and downstream antifibrotic effects of TCPL/HSFs@siRNA (Figure 5J) [42]. The immune‐evasive potential of TCPL/HSFs was further evaluated by assessing their uptake by THP‐1‐derived macrophages. Flow cytometry showed that the uptake of TCPL@Cy5‐siRNA was 23.2±0.5%, whereas that of TCPL/HSFs@Cy5‐siRNA decreased to 12.82±1.2%, indicating that HSFs membrane hybridization reduced macrophage uptake (Figure S11). Proteomic analysis further revealed high expression of the complement regulatory protein CD59 in HSFs (protein abundance = 36,358.24, log2FC = 4.9, FDR = 0.0003), suggesting that HSFs‐derived membranes may provide endogenous immunoregulatory components that contribute to reduced immune recognition and clearance. Taken together, these results demonstrate that TCPL/HSFs not only exhibit excellent cellular uptake and intracellular delivery performance but also rely on membrane homology to achieve specific active targeting. Combined with their robust lysosomal escape capacity, the system establishes a solid foundation for efficient scar‐targeted siRNA delivery.

### 3.6 Evaluation of Transdermal Delivery Capability

**FIGURE 6:**
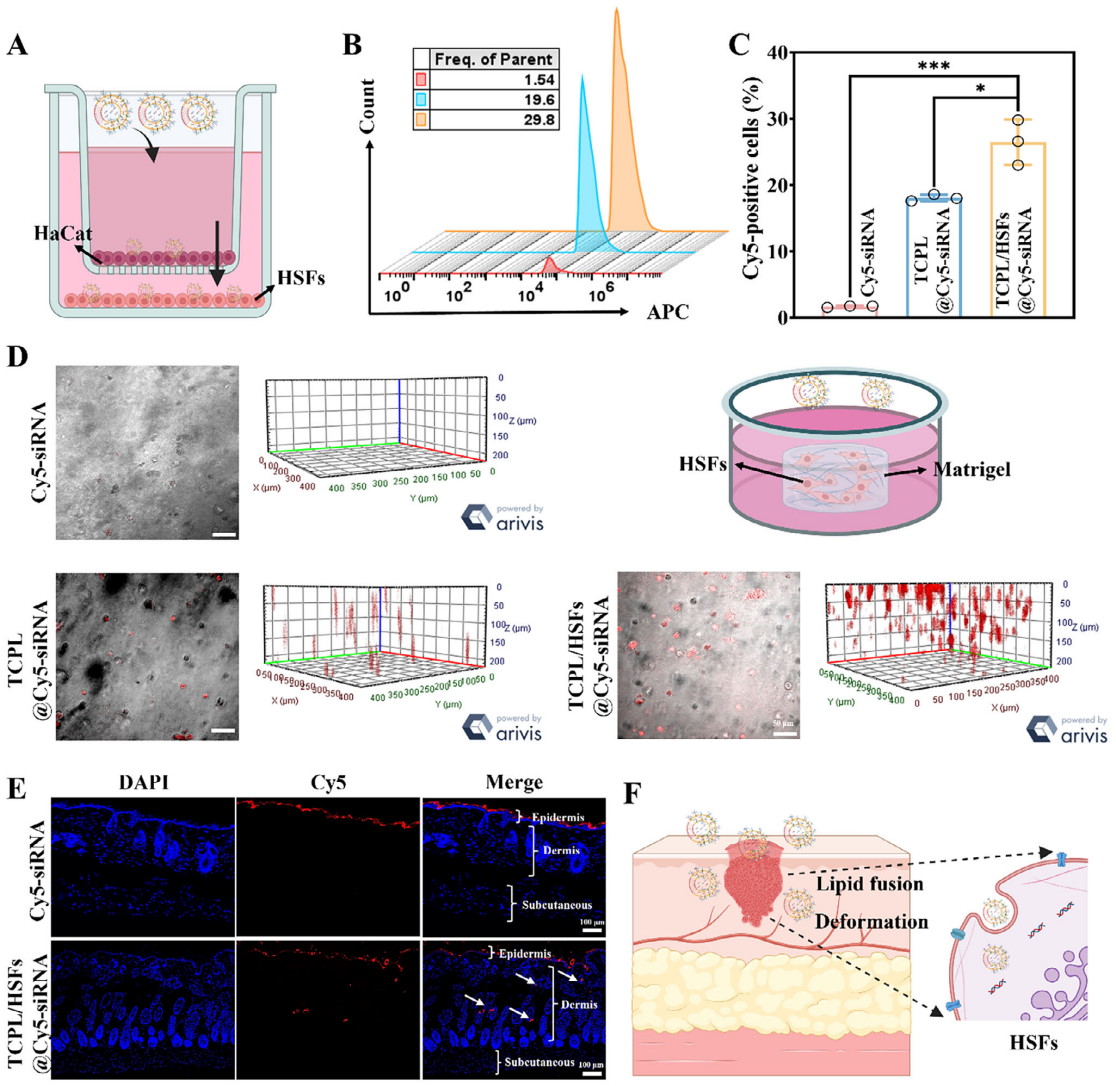
Evaluation of transdermal penetration capability in vitro and in vivo. (A) Schematic illustration of the epithelial barrier model, schematic illustrations were created with BioRender.com. (B) Flow cytometric analysis of TCPL/HSFs@Cy5‐siRNA uptake by HSFs in the lower chamber of the Transwell model. Freq. of Parent represents the percentage of cells within the Cy5‐positive gate relative to the total number of cells in its immediately preceding parent gate. (C) Quantitative analysis of the percentage of Cy5‐positive HSFs following treatment with TCPL/HSFs@Cy5‐siRNA. (D) Establishment of the Matrigel‐based 3D model and 3D confocal imaging of Cy5‐siRNA distribution after 24 h of incubation, schematic illustrations were created with BioRender.com. (E) Penetration of TCPL/HSFs@Cy5‐siRNA in the epidermis, dermis and subcutaneous tissue of mouse dorsal skin, (scale bar = 100 µm). (F) Schematic illustration of TCPL/HSFs‐mediated transdermal delivery of siRNA, schematic illustrations were created with BioRender.com. n = 3, biological replicates, *P < 0.05; ***P < 0.001;.

To assess the transdermal delivery potential of TCPL/HSFs in scar therapy, we employed a multi‐level evaluation strategy using a cell barrier model, a Matrigel‐based 3D model, and an in vivo mouse model. First, an in vitro Transwell system containing HaCaT was established to mimic the epidermal barrier and examine whether siRNA could penetrate the epithelial layer and reach dermal fibroblasts. Flow cytometry revealed that free Cy5‐siRNA showed negligible penetration into the lower chamber HSFs (1.54%), TCPL@Cy5‐siRNA achieved modest improvement (19.6%), whereas TCPL/HSFs@Cy5‐siRNA significantly enhanced penetration to 29.8% (Figure 6A–C), indicating more efficient delivery of siRNA across the epithelial barrier followed by cellular uptake by recipient fibroblasts. Next, a dense Matrigel model embedded with HSFs was constructed to simulate the compact extracellular matrix (ECM) microenvironment of scar tissue. Confocal 3D imaging demonstrated that free Cy5‐siRNA failed to effectively infiltrate the gel, while TCPL/HSFs@Cy5‐siRNA penetrated deeply and distributed widely within the matrix (Figure 6D and Figure S12), suggesting strong ECM infiltration capability and the potential to overcome structural barriers of scar tissue for homotypic targeting. Finally, in vivo transdermal delivery experiments were conducted on the dorsal skin of mice. After 24 h, skin sections were harvested and stained with DAPI for nuclei, free Cy5‐siRNA showed negligible entry into skin tissue, whereas TCPL/HSFs@Cy5‐siRNA exhibited distinct red fluorescence penetrating into both the epidermal and dermal layers (Figure 6E and Figure S13). Combined with the schematic illustration of lipid fusion and ultradeformability mechanisms (Figure 6F), these findings further confirmed that the transfersomes achieve efficient in vivo transdermal delivery through structural deformability and membrane homology.

### 3.7 Investigation of transdermal delivery mechanisms

**FIGURE 7:**
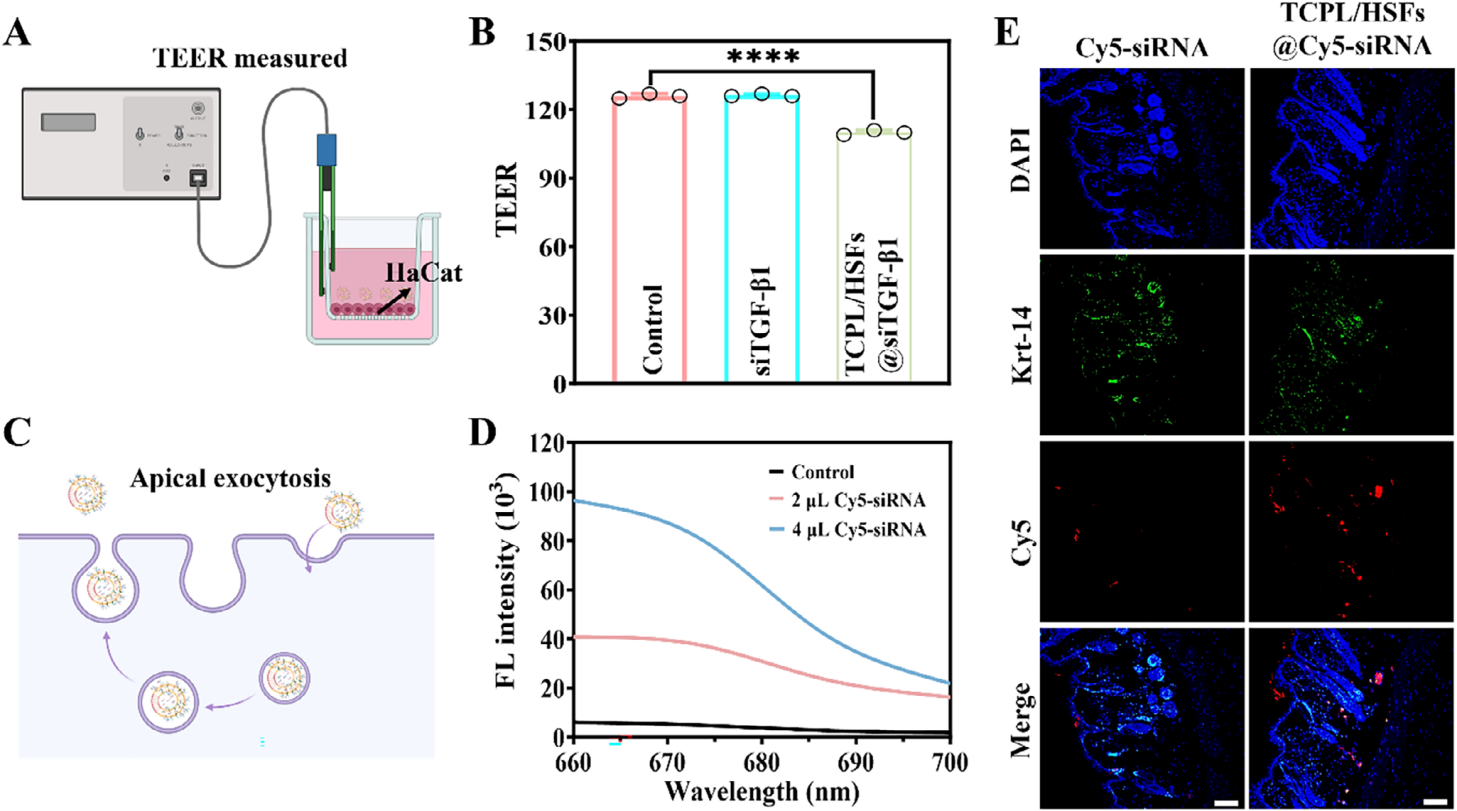
Investigation of transdermal delivery mechanisms. (A) Schematic illustration of transepithelial electrical resistance (TEER) measurement, schematic illustrations were created with BioRender.com. (B) Effect of TCPL/HSFs@siTGF‐β1 on TEER values in the HaCaT monolayer cell model (n = 3, biological replicates). (C) Schematic illustration of nanoparticle transcellular transport via endocytosis and apical exocytosis, schematic illustrations were created with BioRender.com. (D) Changes in Cy5 fluorescence intensity in the supernatant of HaCaT. (E) Representative confocal images showing co‐localization of keratin‐14 with TCPL/HSFs@Cy5‐siRNA in mouse skin. ****P < 0.0001.

Transdermal delivery of materials generally proceeds through three main pathways: intercellular routes, transcellular transport, and skin appendages [43]. First, a HaCaT monolayer Transwell model was established, and transepithelial electrical resistance (TEER) was monitored in real time (Figure 7A). Monolayers were considered intact only when the initial TEER values reached a stable level of 128 ± 1.5 Ω·cm^2^. The cells were then treated with siTGF‐β1 or TCPL/HSFs@siTGF‐β1. After 24 h, the TEER values in the untreated control group and the siRNA group were 126 ± 1.0 and 126.3 ± 0.6 Ω·cm^2^, respectively, indicating that free siRNA had little effect on TEER. In contrast, treatment with TCPL/HSFs@siTGF‐β1 significantly reduced the TEER value to 110 ± 1 Ω·cm^2^ (Figure 7B), suggesting that it may partially loosen tight junction organization and increase paracellular permeability, thereby enhancing transdermal transport efficiency. After removal of the formulation and replacement with fresh medium, TEER values gradually recovered toward baseline, suggesting that the barrier modulation induced by TCPL/HSFs@siTGF‐β1 is transient and reversible (Figure S14). The expression of the tight‐junction‐associated protein ZO‐1 in HaCaT was further examined to investigate the potential mechanism by which TCPL/HSFs regulate tight junctions. ZO‐1 is an important protein that contributes to the maintenance of tight‐junction structure and epithelial barrier integrity. As shown in Figure S15, both TCPL/HSFs and TCPL/HSFs@siTGF‐β1 significantly reduced ZO‐1 expression, with a more pronounced decrease observed in the TCPL/HSFs group. This result may be related to the stronger barrier regulatory role of TCPL/HSFs and their positively charged surface properties. Together with the decreased TEER, these results support that TCPL/HSFs transiently modulate tight‐junction integrity and facilitate paracellular transport across the keratinocyte barrier. Second, to examine the transcellular behavior of the complexes (Figure 7C), changes in Cy5 fluorescence intensity in the culture supernatant were measured. Compared with the Cy5‐siRNA group, the TCPL/HSFs@Cy5‐siRNA group showed dose‐dependent increases in supernatant fluorescence, indicating that the system enters cells via endocytosis and is exocytosed apically, demonstrating high transcellular transport efficiency (Figure 7D). Finally, a mouse skin model was employed to investigate the role of skin appendages. Hair follicles and sweat glands were labeled with Krt14, while DAPI and Cy5 signals were used to track nuclei and siRNA distribution, respectively. Confocal imaging revealed that free Cy5‐siRNA remained confined to the skin surface, whereas TCPL/HSFs@Cy5‐siRNA penetrated deeply into the dermis via appendageal structures. Strong co‐localization of red Cy5 signals with green Krt14 signals confirmed that the complexes exploit appendageal pathways such as hair follicles and sweat glands to form “penetration channels” that facilitate deeper delivery (Figure 7E). Notably, the distribution and integrity of skin appendages in hypertrophic scar tissue differ substantially from those in normal skin. Following deep dermal injury, hair follicles and sweat glands within hypertrophic scars may be reduced, damaged, or even absent, and the remaining appendageal structures may exhibit abnormal morphology and distribution. Therefore, the contribution of appendageal pathways to transdermal transport may be lower in hypertrophic scars than in normal skin and may vary depending on scar depth and maturation stage. In addition, mouse dorsal skin differs from human skin in terms of appendage distribution and barrier properties. It lacks widely distributed sweat glands and generally exhibits a thinner skin barrier and higher permeability. In summary, the transdermal mechanism of TCPL/HSFs@siTGF‐β1 involves multiple complementary routes: (i) enhancing paracellular permeability by reducing TEER values, (ii) achieving efficient transcellular transport through endocytosis and apical exocytosis, and (iii) utilizing hair follicle‐associated appendageal structures as an additional pathway for deeper penetration.

### 3.8 Evaluation of transdermal transfection capability

**FIGURE 8:**
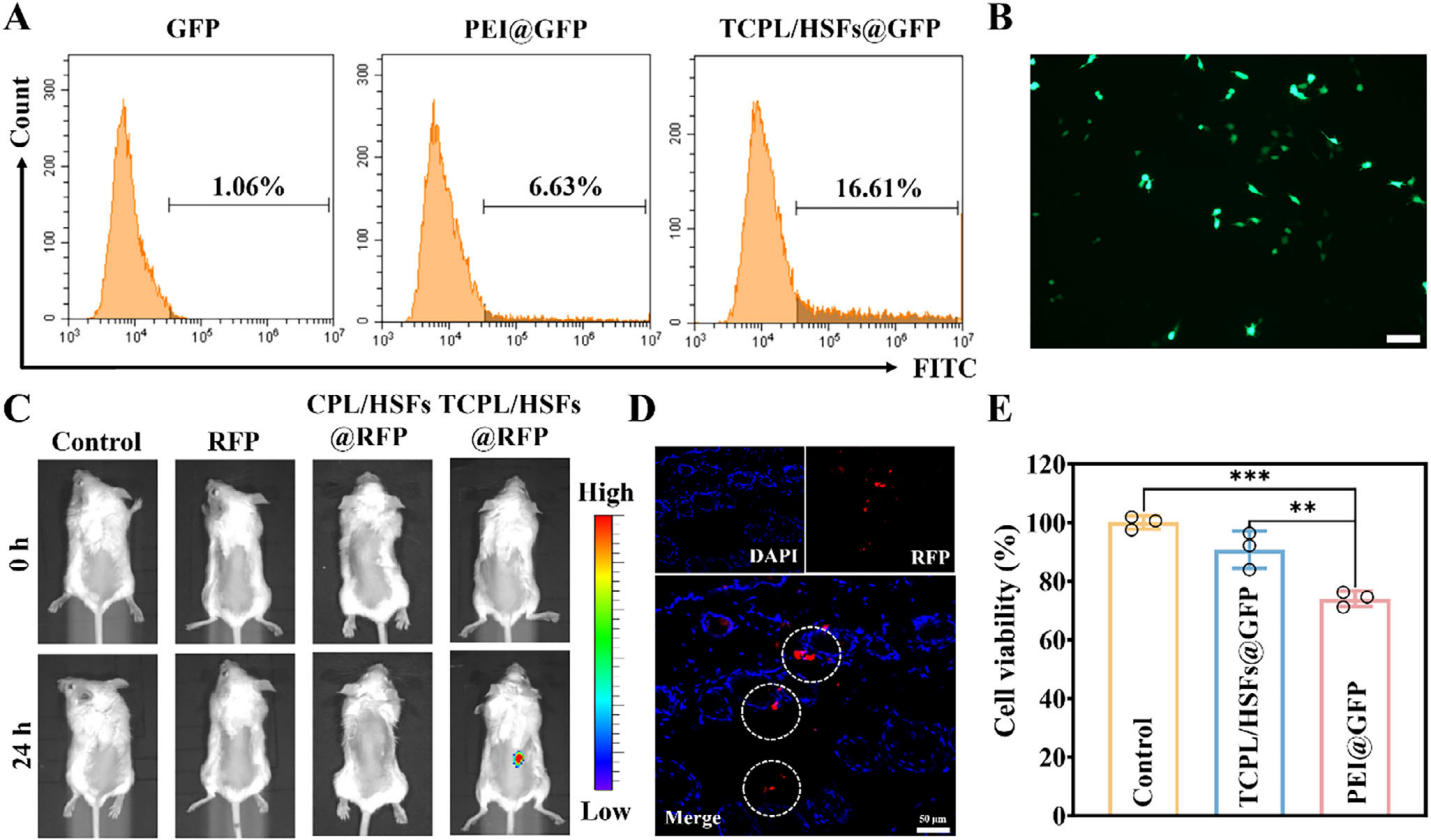
Validation of transdermal transfection capability. (A) Quantitative analysis of transfection efficiency of TCPL/HSFs@GFP and PEI‐25000@GFP (w/w = 1.3:1) in HSFs. (B) Fluorescence images showing GFP expression in HSFs mediated by TCPL/HSFs (scale bar = 100 µm). (C) In vivo expression of RFP in the dorsal skin of mice after topical application of TCPL/HSFs@RFP (n = 3, biological replicates). (D) Confocal microscopy images of RFP expression in mouse skin (scale bar = 50 µm). (E) Cytotoxicity analysis of TCPL/HSFs@GFP and PEI‐25000@GFP (w/w = 1.3:1) in HSFs (n = 3, biological replicates). **P < 0.01; ***P < 0.001.

To complement the siRNA‐based findings, GFP/RFP plasmid delivery was used as a supplementary functional assay to evaluate whether TCPL/HSFs could also support the delivery and expression of larger nucleic acid cargo. In vitro, TCPL/HSFs@GFP achieved a GFP‐positive rate of 16.61% in HSFs, significantly higher than GFP solution (1.06%) and the positive control (6.63%) (Figure 8A). Confocal microscopy further confirmed efficient intracellular plasmid delivery and GFP expression (Figure 8B). In vivo, TCPL/HSFs@RFP generated distinct fluorescence signals in mouse skin, and tissue sections confirmed penetration into deeper skin layers and intracellular plasmid expression (Figure 8C,D). Moreover, TCPL/HSFs@GFP exhibited markedly lower cytotoxicity than the high‐molecular‐weight PEI control, with cell viability comparable to that of untreated cells (Figure 8E). HDF were included as an additional control for the evaluation of cell‐selective transfection. TCPL/HSFs@GFP resulted in GFP‐positive rates of 17.50 ± 0.56%, 8.83±1.24%, and 7.97 ± 0.21% in HSFs, HDF, and HaCaT, respectively (Figure S16A–C). Notably, the transfection efficiency in HSFs was approximately 2‐fold higher than that in HDF, further supporting the preferential transfection of TCPL/HSFs toward homologous scar fibroblasts. Untreated cells and blank TCPL/HSFs were included as negative controls, both showing less than 1% GFP‐positive cells, indicating minimal cellular autofluorescence and carrier‐related background signals (Figure S16D). Although HaCaT exhibited a more pronounced high‐fluorescence tail, their overall percentage of Cy5‐positive cells remained substantially lower than that of HSFs. This tail likely reflects a small subpopulation of HaCaT with strong Cy5‐associated signals, potentially due to cellular heterogeneity and nonspecific electrostatic interactions between the positively charged TCPL/HSFs and the cell membrane.

### 3.9 In Vitro Antifibrotic Evaluation

**FIGURE 9:**
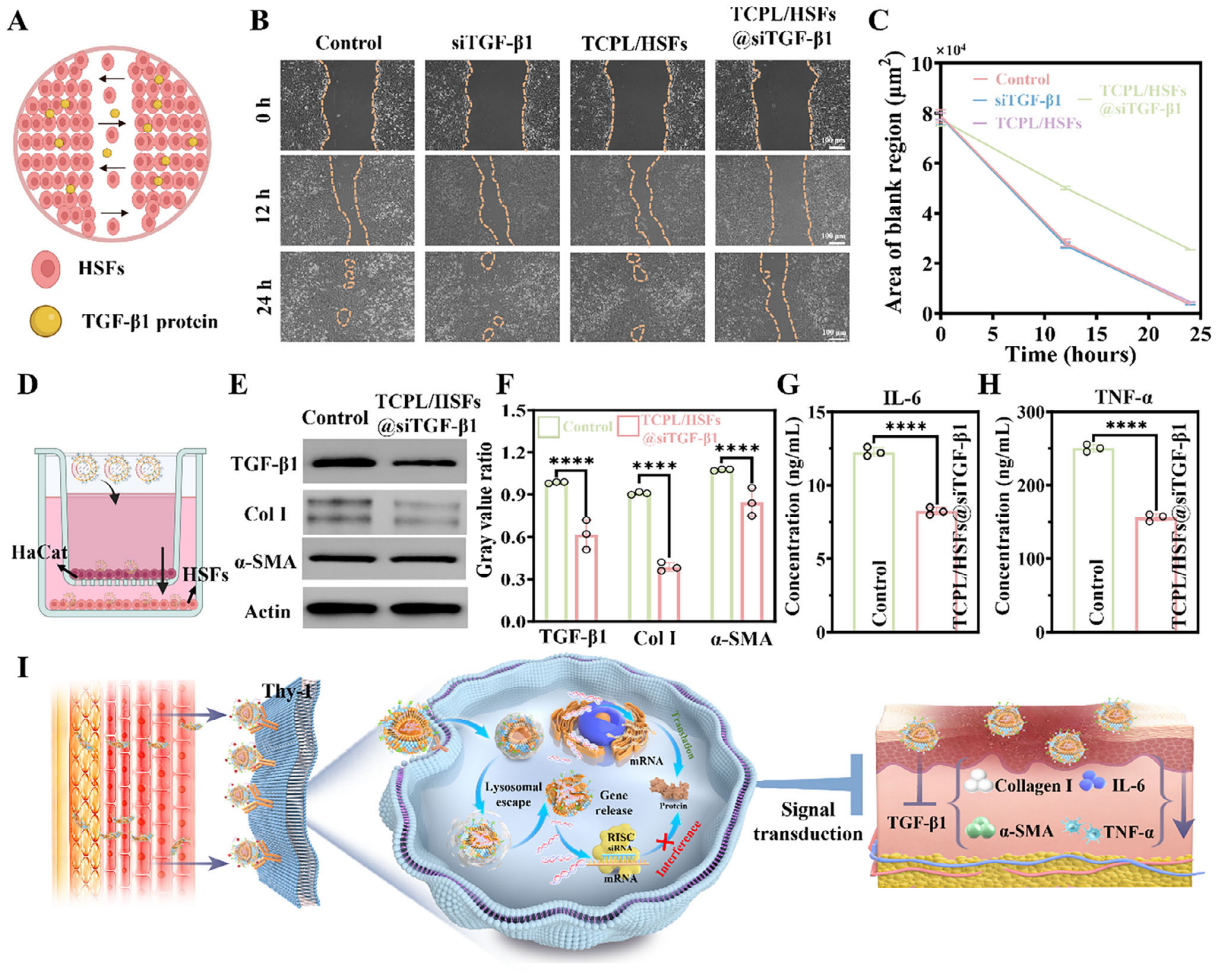
In vitro antifibrotic evaluation. (A) Schematic illustration of exogenous TGF‐β1 protein stimulation in HSFs. (B) Representative images showing the inhibitory effect of TCPL/HSFs@siTGF‐β1 on HSFS migration at 12 and 24 h (scale bar = 100 µm). (C) Quantitative analysis of the blank area in the migration assay (n = 3, biological replicates). (D) Schematic illustration of the HaCaT/HSFs co‐culture Transwell model simulating the skin microenvironment, schematic illustrations were created with BioRender.com. (E–F) Qualitative and quantitative analysis of key fibrotic protein expression (TGF‐β1, Col I, and α‐SMA) in HSFs. (G) Expression levels of the IL‐6 inflammatory cytokines in the supernatant of the lower Transwell chamber (n = 3, biological replicates). (H) Expression levels of the TNF‐α inflammatory cytokines in the supernatant of the lower Transwell chamber (n = 3, biological replicates). (I) Schematic diagram of the targeting, penetration, gene silencing, and anti‐fibrotic processes of TCPL/HSFs@siTGF‐β1. ****P < 0.0001.

To assess the antifibrotic potential of TCPL/HSFs@siTGF‐β1 on fibroblasts, we first examined its effects on HSFs migration using a scratch assay. As shown in Figure 9A,B, treatment with exogenous TGF‐β1 significantly enhanced the migratory ability of HSFs. Neither siTGF‐β1 nor TCPL/HSFs group alone altered cell migration, whereas the TCPL/HSFs@siTGF‐β1 group maintained the widest scratch gap and smallest migratory area at both 12 and 24 h, indicating a pronounced inhibitory effect on TGF‐β1–induced fibroblast migration (Figure 9C). To distinguish reduced migration from possible antiproliferative effects, an additional proliferation assay was performed under the same treatment conditions. Figure S17A showed no significant inhibition of HSFs proliferation, suggesting that the scratch assay primarily inhibited HSFs migration through anti‐fibrosis. Next, a HaCaT/HSFs co‐culture Transwell model was established to mimic the skin microenvironment. Western blot analysis of HSFs demonstrated that key fibrotic markers, including TGF‐β1, collagen I (Col I), and α‐smooth muscle actin (α‐SMA), were all markedly downregulated in the TCPL/HSFs@siTGF‐β1 group (Figure 9D–F), suggesting that the system effectively suppressed fibroblast phenotypic transformation and extracellular matrix deposition, thereby exerting antifibrotic effects at the molecular level [44]. To validate the antifibrotic effect at the transcriptional level, qRT‐PCR analysis of fibrosis‐related genes was performed. TCPL/HSFs@siTGF‐β1 significantly reduced the mRNA expression of COL1A1 and α‐SMA. which was consistent with the corresponding protein‐level changes (Figure S18). In addition, ELISA analysis of the culture supernatants from the lower chamber revealed that pro‐inflammatory cytokines IL‐6 and TNF‐α were significantly reduced in the TCPL/HSFs@siTGF‐β1 group compared with the control (Figure 9G,H). The potential antifibrotic effect of the carrier itself was further examined by evaluating the expression of TGF‐β1 and α‐SMA following treatment with TCPL/HSFs. As shown in Figures S19A,B, TCPL/HSFs alone caused no obvious changes in TGF‐β1 or α‐SMA expression compared with the untreated group, whereas TCPL/HSFs@siTGF‐β1 markedly reduced the expression of both proteins. These findings indicate that the observed antifibrotic effects were primarily attributable to TCPL/HSFs‐mediated siTGF‐β1 delivery and subsequent TGF‐β1 silencing rather than to the carrier itself. To further assess epidermal safety, HaCaT viability was evaluated under the corresponding treatment conditions. No significant reduction in HaCaT viability was observed, indicating that the treatment did not cause overt toxicity to epidermal cells (Figure S17B). A graphical summary illustrating the overall therapeutic mechanism of TCPL/HSFs@siTGF‐β1 has been added to facilitate understanding of the targeting, penetration, gene silencing, and antifibrotic processes (Figure 9I). TCPL/HSFs@siTGF‐β1 not only effectively inhibited TGF‐β1–induced fibroblast migration but also downregulated fibrotic protein expression and inflammatory cytokine levels, demonstrating robust antifibrotic potential in vitro.

### 3.10 In Vivo Therapeutic Effects in a Rabbit Ear Hypertrophic Scar Model

**FIGURE 10:**
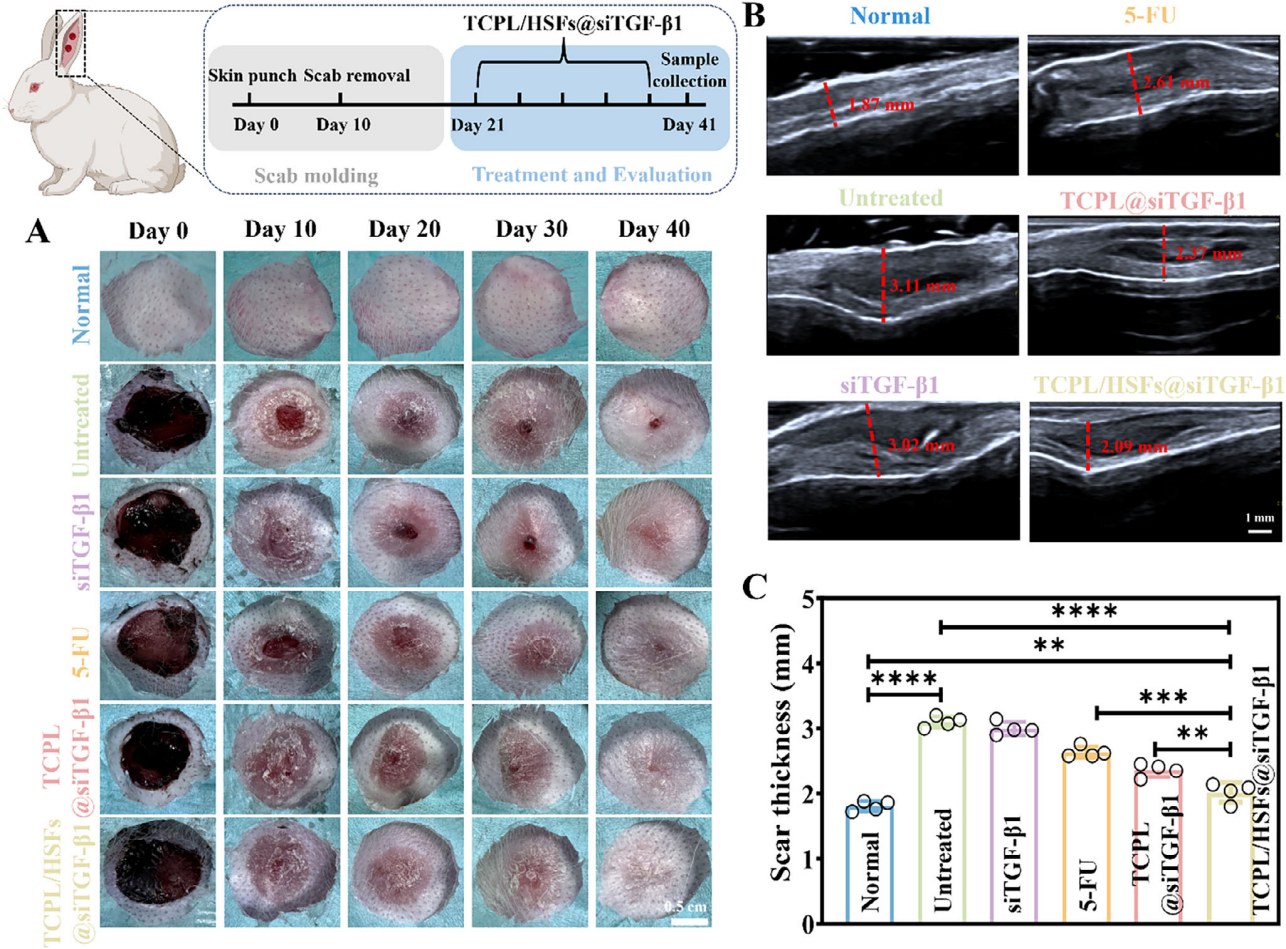
Establishment of the rabbit ear hypertrophic scar model and subsequent treatments. (A) Representative photographs of hypertrophic scar sites on rabbit ears at predetermined time points after various treatments. (B) Ultrasound imaging of hypertrophic scar sites on rabbit ears from each group at day 40 (scale bar = 1 mm). (C) Quantitative analysis of skin and scar thickness in the normal skin and experimental groups (n = 4, biological replicates). **P < 0.01; ***P < 0.001; ****P < 0.0001.

To evaluate the therapeutic potential of the system against hypertrophic scar in vivo, a full‐thickness skin defect model was established on the ears of New Zealand white rabbits to induce typical hypertrophic scar formation. Animal experiments were conducted in accordance with the regulations of the Animal Ethics Committee of Jinan University (Ethics No. IACUC‐ 20250508‐11). Scar fibroblast membranes were extracted from rabbit scar tissue for the construction of delivery materials, and interventions were initiated on day 21 post‐modeling, when the wounds had largely completed re‐epithelialization and entered the active scar‐proliferative stage, thereby minimizing interference with early wound healing and allowing evaluation of the therapeutic effect on established hypertrophic scars [45]. First, the transdermal delivery capability of TCPL/HSFs in scar tissue was assessed. As shown in Figure S20, skin sections harvested at 12 h post‐application revealed distinct red fluorescence signals of TCPL/HSFs@Cy5‐siRNA within the tissue, confirming efficient penetration. After 21 days of treatment, macroscopic observation revealed significant differences in scar morphology among the groups. In the untreated and siTGF‐β1 groups, scar edges remained raised with a deep red coloration, presenting typical hypertrophic characteristics. In contrast, the TCPL/HSFs@siTGF‐β1 group exhibited the flattest scar surface with coloration closely resembling normal skin, showing remarkable scar attenuation, superior even to the 5‐FU positive group and TCPL@siTGF‐β1 group without cell membrane hybridization (Figure 10A) [46]. High‐frequency ultrasound imaging was further employed for noninvasive measurement of scar thickness across groups [34]. As shown in Figure 10B, normal skin exhibited a thickness of approximately 1.87 mm, whereas untreated scars thickened significantly to 3.11 mm. Treatment with TCPL/HSFs@siTGF‐β1 reduced scar thickness to 2.09 mm, which was significantly thinner than that observed in the TCPL@siTGF‐β1 group (2.37 mm) and the 5‐FU group (2.61 mm), with statistical significance (Figure 10C). These results highlight the strong inhibitory effect of the system on scar tissue hyperplasia. The current results demonstrate the therapeutic efficacy of TCPL/HSFs@siTGF‐β1 in actively proliferating hypertrophic scars; however, their efficacy in long‐established mature hypertrophic scars requires further validation in future studies using long‐term treatment models.

### 3.11 Histological Evaluation of Scar Tissue

**FIGURE 11:**
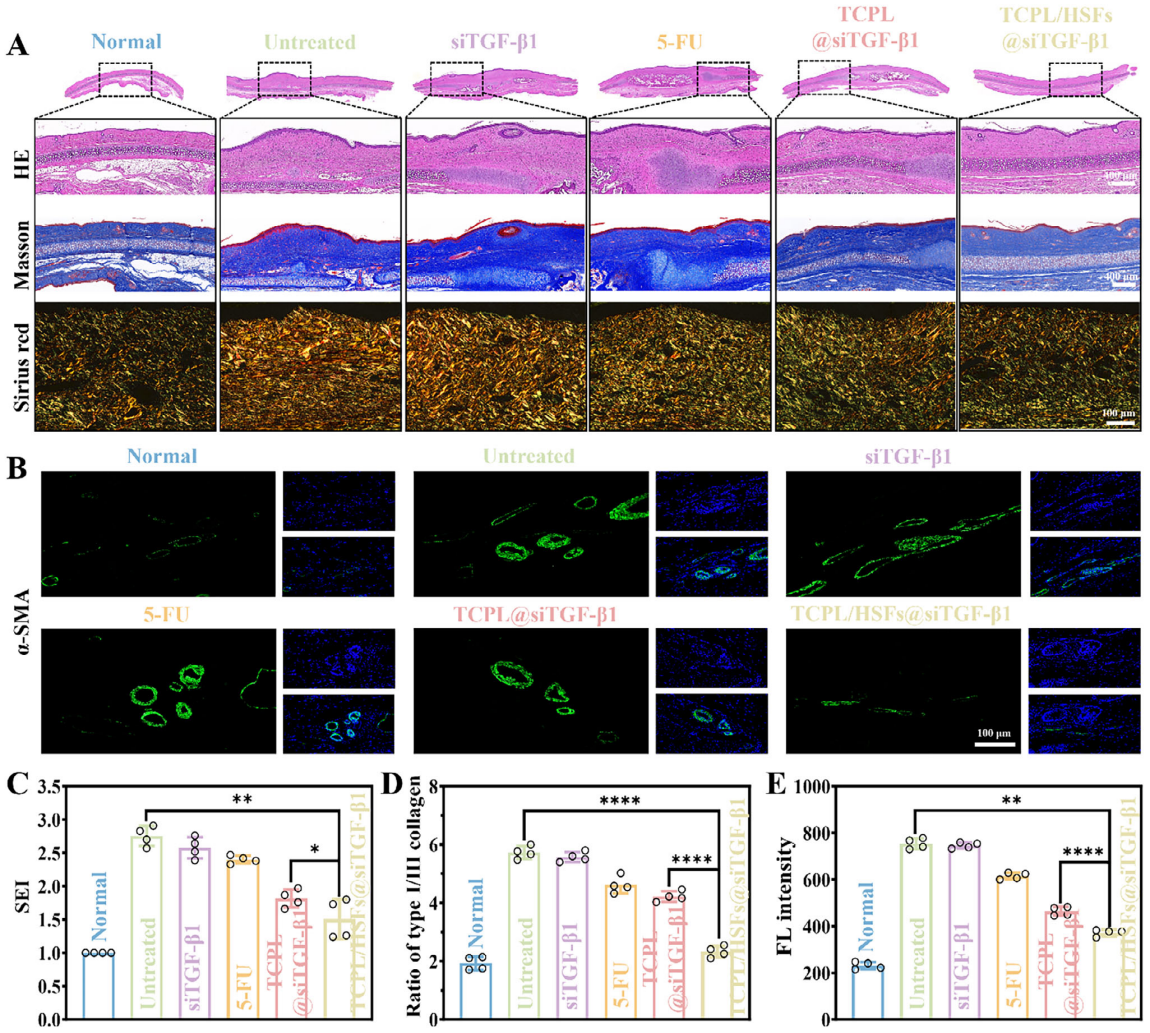
Histological evaluation of rabbit ear hypertrophic scar tissue. (A) Representative H&E, Masson's trichrome, and Sirius Red staining of scar tissues (scale bars = 400, 400, and 100 µm). (B) Immunofluorescence staining showing α‐SMA (green) expression in scar tissues (scale bar = 100 µm). (C) Quantitative results of the scar elevation index (SEI). (D) Quantitative analysis of the collagen type I/III ratio. (E) Fluorescence intensity of α‐SMA–positive cells in skin tissues (n = 4, biological replicates). *P < 0.05; **P < 0.01; ****P < 0.0001.

Histological analyses revealed significant differences in scar tissue repair and fibrotic deposition among groups (Figure 11A). H&E staining showed that untreated scars exhibited markedly thickened epidermis, pronounced hyperkeratosis, and disorganized dermal collagen fibers [47]. In contrast, both 5‐FU, TCPL@siTGF‐β1 and TCPL/HSFs@siTGF‐β1 treatment alleviated epidermal hyperplasia to varying degrees, with the TCPL/HSFs@siTGF‐β1 group showing the most normalized scar morphology, as reflected by a scar elevation index (SEI) value closest to that of normal skin (Figure 11C) [48]. Masson's trichrome staining further revealed excessive and disordered collagen deposition in the untreated group, whereas collagen accumulation was substantially reduced and displayed more uniform organization in the TCPL/HSFs@siTGF‐β1 group (Figure S21). Sirius Red staining under polarized light confirmed these findings, demonstrating that collagen ratios and fiber organization in this group most closely resembled those of normal skin (Figure 11D). Immunofluorescence staining (Figure 11B,E) revealed strongly elevated α‐smooth muscle actin (α‐SMA) signals in the untreated group, indicative of extensive myofibroblast activation, a key driver of persistent scar contraction and fibrosis. Notably, the TCPL/HSFs@siTGF‐β1 group displayed the fewest α‐SMA–positive cells, highlighting its superior antifibrotic activity [49]. Complementary Tunel staining further demonstrated that TCPL/HSFs@siTGF‐β1 treatment effectively induced apoptosis of aberrant fibroblasts in scar tissue, reinforcing its ability to suppress fibrotic progression (Figure S22). These results suggest that while conventional agents such as 5‐FU provide partial improvements in scar architecture, their therapeutic efficacy remains limited. By contrast, the targeted synergistic effect of TCPL/HSFs@siTGF‐β1 not only significantly suppressed abnormal epidermal hyperplasia and collagen over‐deposition but also attenuated myofibroblast activation and promoted their apoptosis. Thus, TCPL/HSFs@siTGF‐β1 intervenes at multiple levels of scar pathogenesis, demonstrating superior therapeutic potential for anti‐scar therapy. The experimental results directly support a reduction in scar tissue formation, as evidenced by the decrease in SEI, the reduction in the collagen I/III ratio, and the decreased expression of α‐SMA. However, the remodeling of the fibrotic microenvironment through specific signaling pathways requires further investigation and validation.

### 3.12 Evaluation of Biocompatibility and Immunological Safety

**FIGURE 12:**
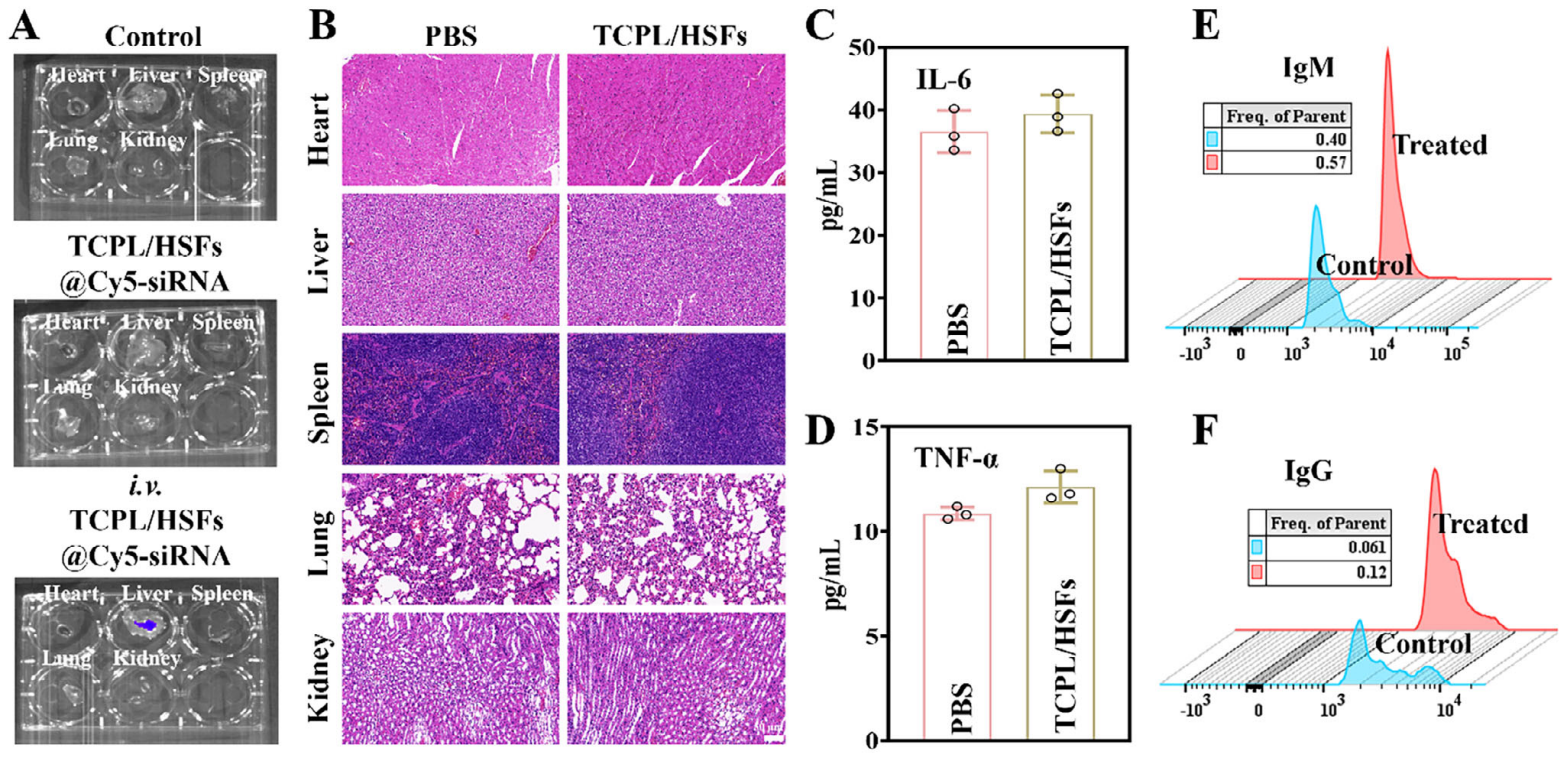
Evaluation of biocompatibility and immunological safety. (A) In vivo fluorescence imaging of major organs in mice after Cy5‐siRNA administration via tail vein injection or topical application. (B) H&E staining of major organs in mice (scale bar = 50 µm). (C–D) Quantification of IL‐6 and TNF‐α levels in mouse serum. (E–F) Expression of IgM and IgG specific antibodies in serum from treated and untreated mice (n = 3, biological replicates).

To assess the biosafety and immunogenicity of the TCPL/HSFs, a series of experiments were conducted focusing on organ toxicity, immune responses, and the ability of transdermal delivery to circumvent hepatic first‐pass metabolism. To provide a conservative assessment of the intrinsic systemic safety of TCPL/HSFs under maximal exposure conditions, an intravenous administration model was used. This experiment was not intended to directly mimic the topical delivery route, but rather to evaluate potential systemic toxicity and humoral immunogenicity under a worst‐case exposure scenario. First, after 4 weeks of continuous tail vein administration, the major organs of mice were collected for H&E staining. As shown in Figure 12B, compared with the PBS group, the TCPL/HSFs‐treated group displayed intact histological structures in the heart, liver, spleen, lungs, and kidneys, without evidence of inflammatory cell infiltration or pathological degeneration. Moreover, no significant differences in serum inflammatory cytokines were observed (Figure 12C,D), confirming good tissue compatibility in vivo. Second, serum flow cytometry was employed to detect IgM and IgG antibody levels, in order to evaluate potential specific immune responses elicited by the material. As shown in Figure 12E,F, IgM and IgG expression levels were relatively low between the PBS and TCPL/HSFs groups, suggesting that the system does not induce overt immunogenic reactions and possesses favorable immunological safety. As shown in Figure S23A–D, in addition to histological and immunological evaluations, serum biochemical analyses were performed to further assess systemic safety. No significant differences were observed in ALT, AST, BUN, or creatinine levels between the TCPL/HSFs‐treated group and the control group, indicating the absence of obvious hepatic or renal toxicity. Finally, to investigate whether transdermal delivery of TCPL/HSFs could bypass hepatic first‐pass metabolism, Cy5‐siRNA was administered either via tail vein injection or topical application. In vivo fluorescence imaging revealed pronounced hepatic accumulation in the injection group, indicating siRNA clearance through hepatic metabolic pathways. By contrast, in the topical group, fluorescence was predominantly distributed at the local skin site with no detectable hepatic signal (Figure 12A). These findings demonstrate that transdermal administration effectively circumvents first‐pass hepatic metabolism, thereby improving delivery efficiency while reducing systemic toxicity [36]. The TCPL/HSFs exhibited excellent biocompatibility, low humoral immunogenicity, and effective avoidance of hepatic first‐pass effects, providing a reliable biosafety foundation for its application as a noninvasive transdermal delivery platform.

Conventional lipid‐based or other broad‐spectrum nanocarriers mainly improve siRNA stability and skin penetration, whereas the transfersome system constructed in this study exhibits several distinctive advantages by coupling efficient transdermal transport with active homotypic recognition toward HSFs. This targeting advantage is supported not only by the enriched membrane‐associated molecules identified through proteomic analysis, including Thy‐1, but also by the enhanced uptake and homotypic competition results observed in HSFs. In addition, the penetration capability of this system arises not only from its deformable transfersome structure, but also from multiple complementary pathways, including paracellular transport, transcellular trafficking, and skin appendageal routes. More importantly, beyond tissue penetration, the carrier also achieved enhanced intracellular delivery efficiency, as evidenced by stronger cellular uptake, more effective lysosomal escape, and robust gene transfection performance. Therefore, this platform provides a more integrated solution that simultaneously improves targeting specificity, barrier penetration, and transfection efficiency for transdermal siRNA therapy of hypertrophic scars.

## 4 Conclusions

In this study, we constructed biomimetic hybrid delivery transfersomes (TCPL/HSFs) with active targeting capability, enabling efficient transdermal delivery of TGF‐β1 siRNA (siTGF‐β1) to scar fibroblasts and achieving significant therapeutic efficacy against hypertrophic scar both in vitro and in vivo. Proteomic analysis revealed high and specifi surface expression of the Thy‐1 membrane glycoprotein on primary human scar fibroblasts, which, as a GPI‐anchored protein, provided the molecular basis for targeted design. By hybridizing HSFs membranes with cationic carriers through ultrasonic co‐extrusion and employing electrostatic self‐assembly for siTGF‐β1 loading, a biomimetic vector with active targeting capacity, immune evasion properties, and high transfection efficiency was successfully obtained. The system was able to penetrate skin barriers via multiple pathways—including intercellular routes, transcellular transport, and skin appendages—thereby enabling noninvasive siRNA delivery into the dermis. Experimental results demonstrated that TCPL/HSFs@siTGF‐β1 markedly downregulated TGF‐β1 expression, subsequently suppressing α‐SMA expression, excessive extracellular matrix deposition, and the release of pro‐inflammatory cytokines (TNF‐α, IL‐6), thus exhibiting strong antifibrotic activity. Moreover, the system showed excellent biocompatibility, low humoral immunogenicity, and effective avoidance of hepatic first‐pass metabolism, further enhancing therapeutic safety and delivery efficiency. Overall, this work establishes a biomimetic and potentially translatable transdermal gene delivery platform that integrates targeting specificity, barrier adaptability, and biosafety, thereby offering a scalable framework for precision treatment of hypertrophic scar and other skin‐associated disorders.

## Author Contributions

D.M. and H.X. designed the work. H.X. and D.M. wrote the manuscript. H.X., Z.Z., Y.L., Y.X and Z.W. performed the experiments and collected the data. Z.Z., Y.Y., K.L., and X.Z. helped with some measurements. H.X. analyzed the data. D.M. and Z.Z. provided research funding. All authors have given approval to the final version of the manuscript.

## Funding

This work was financially supported by the National Natural Science Foundation of China (32171369), Guangzhou Science and Technology Project (2023A03J0523), the Excellent Graduate Student Cultivation Program of Jinan University (2026CXB033) and Guangzhou Municipal Science and Technology Bureau (2025A03J3599).

## Ethics Statement

Animal experiments were conducted in accordance with the regulations of the Animal Ethics Committee of Jinan University (Ethics No.: IACUC‐ 20250508‐11).

## Conflicts of Interest

The authors declare no conflicts of interest.

## Supporting information




**Supporting File**: advs77975‐sup‐0001‐SuppMat.pdf.

## Data Availability

The data that support the findings of this study are available on request from the corresponding author.
