# Supplementary material for: Targeted‐Penetrating Biomimetic Hybrid Transfersomes for Enhanced Transdermal Gene Transfection in Hypertrophic Scar Therapy
Source: Adv Sci (Weinh). 2026 Sep 27:e77975. Online ahead of print. doi: 10.1002/advs.77975 (PMC13616318; doi:10.1002/advs.77975)
Supplement: Supplementary file 1 [file ADVS-9999-e77975-s001.pdf]

Targeted-penetrating biomimetic hybrid transfersomes for enhanced transdermal gene transfection in hypertrophic scar therapy

Hui Xing[a, b], Ziyi Zhao[b], Yuhui Yang[b], Yichen Lin[b], Yinglin Xuan[b], Ziyang Wang[b], Kai Lv[b], Xiaoxu Zhu[a], Zhi Zhang[a, *], Dong Ma[a, b, c, *]

[a] Department of Burn and Plastic Surgery, Guangzhou Red Cross Hospital, Jinan University, Guangzhou 510220, China

[b] State Key Laboratory of Bioactive Molecules and Druggability Assessment, Institute of Biomedical Engineering, Jinan University, Guangzhou 510632, China

[c] MOE Key Laboratory of Tumor Molecular Biology, Jinan University, Guangzhou 510632, China

* Corresponding authors.

E-mail addresses: zhangzhicc48@163.com (Zhi Zhang); tmadong@jnu.edu.cn (Dong Ma)

This PDF file includes:

Supplementary Fig. 1 to Fig. 23

Supplementary Table 1 to 5

Supplementary Fig. 1. (A) Proton nuclear magnetic resonance ^{1}H NMR spectrum of Cho-PEI. (B) Infrared spectra of Cho and Cho-PEI.

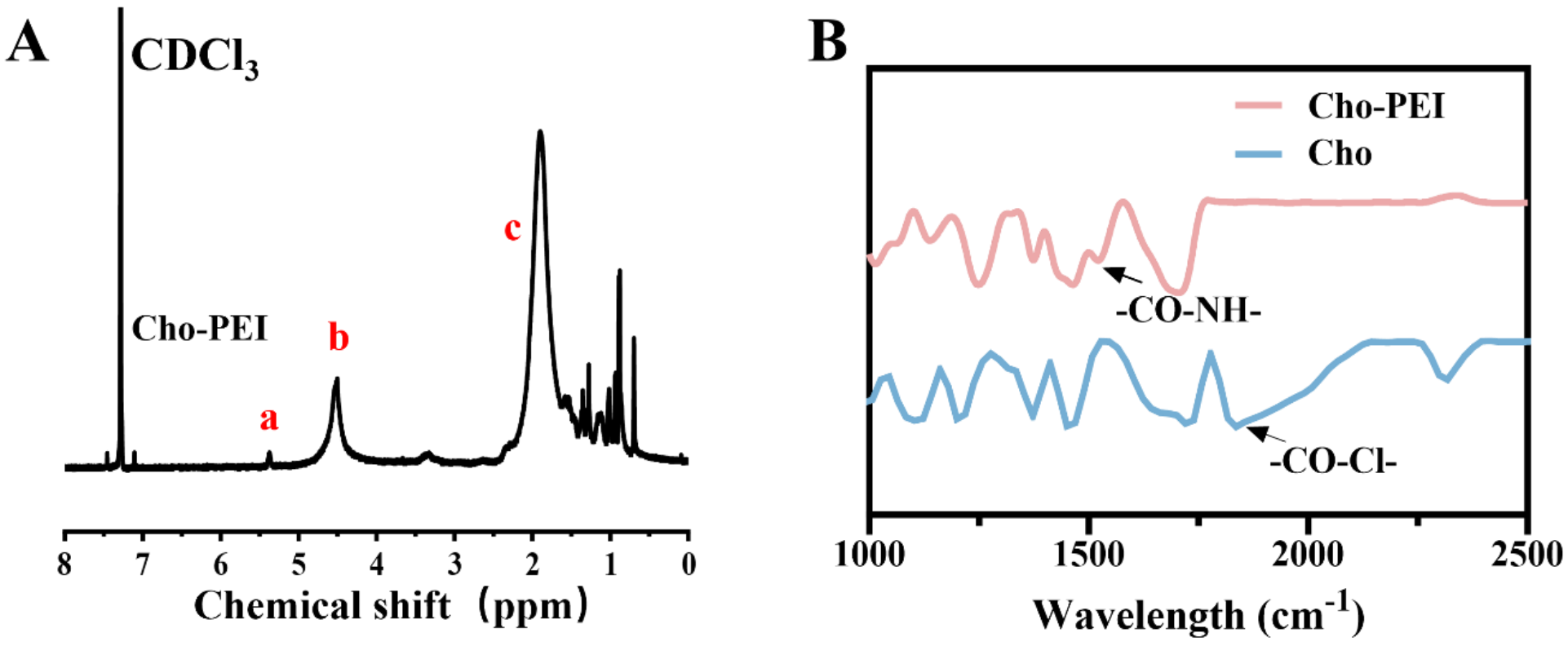

Supplementary Fig. 2. Western blot analysis of membrane-associated marker proteins Na^+/K^+-ATPase and CD44 in isolated HSFs membranes.

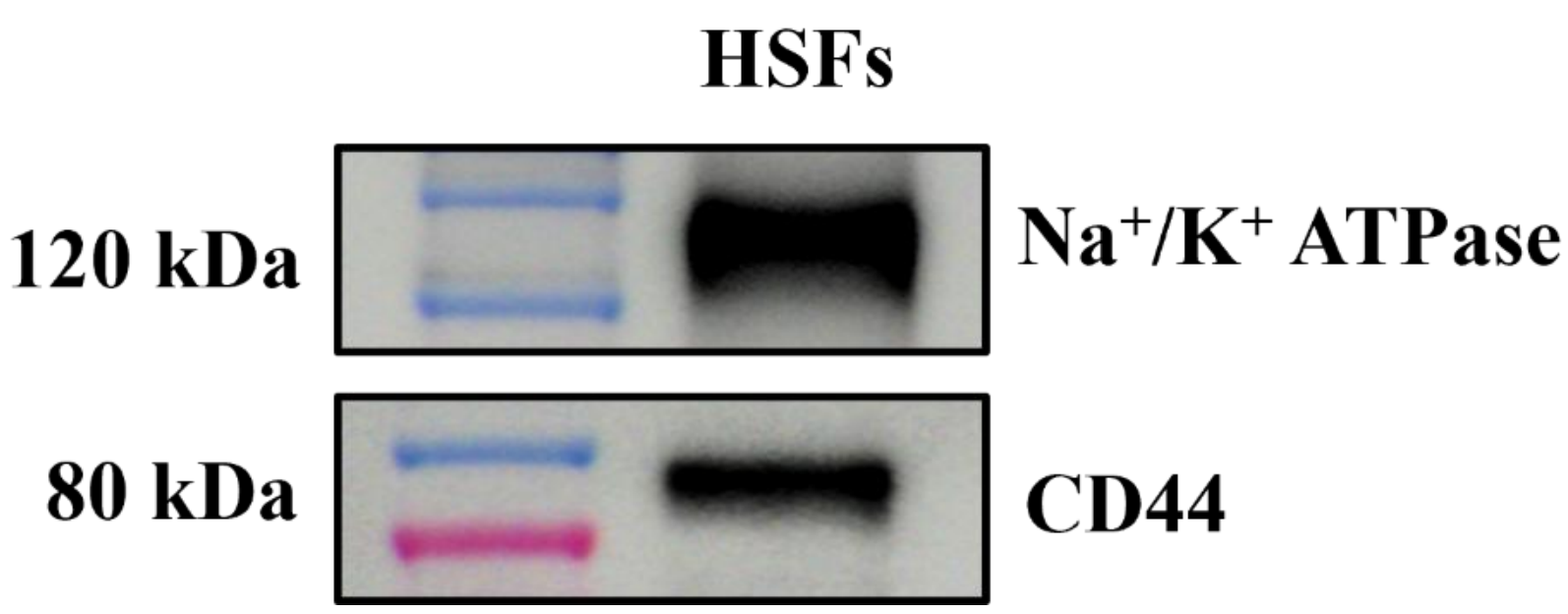

Supplementary Fig. 3. Representative high-resolution transmission electron microscopy (HRTEM) images of TCPL and TCPL/HSFs (scale bar=100 nm).

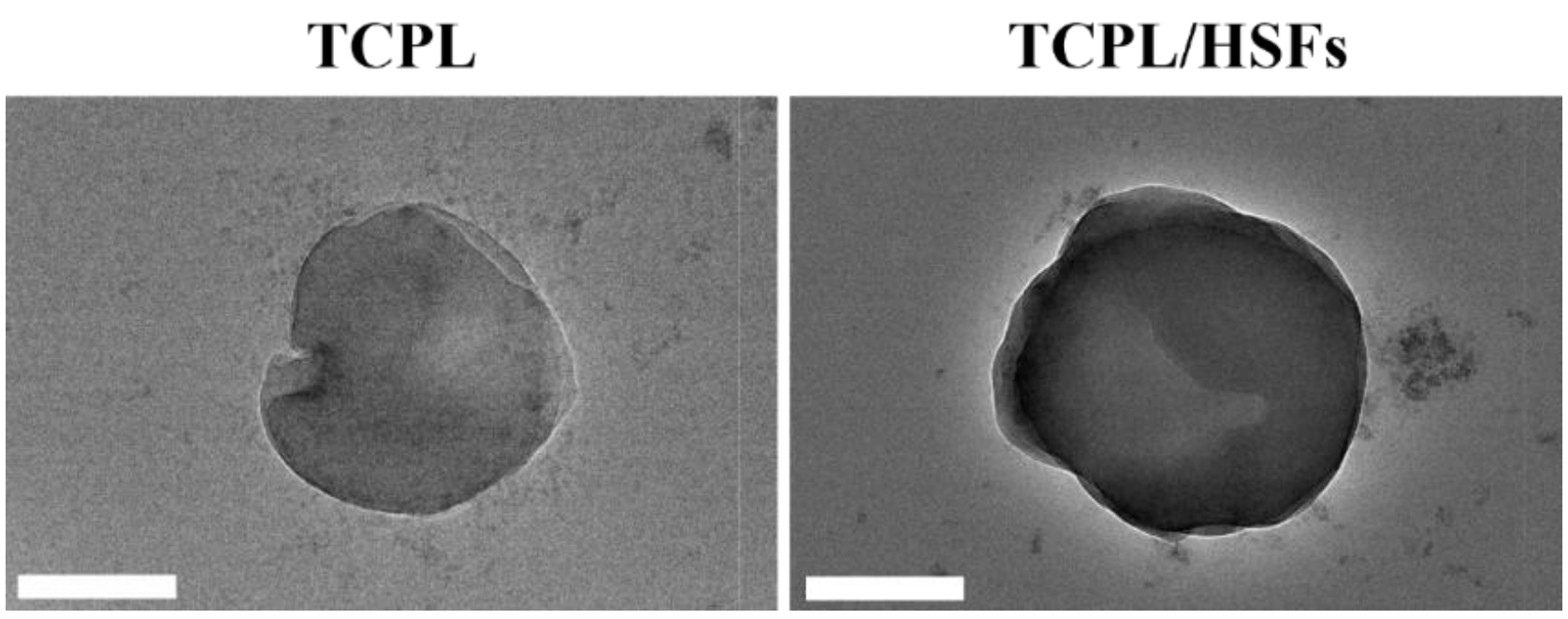

Supplementary Fig. 4. (A) Representative Western blot images of Na^+/K^+-ATPase and CD44 in isolated HSFs membranes and three independently prepared batches of TCPL/HSFs. (B) Densitometric quantification of Na^+/K^+-ATPase and CD44, respectively, (n=3, biological replicates).

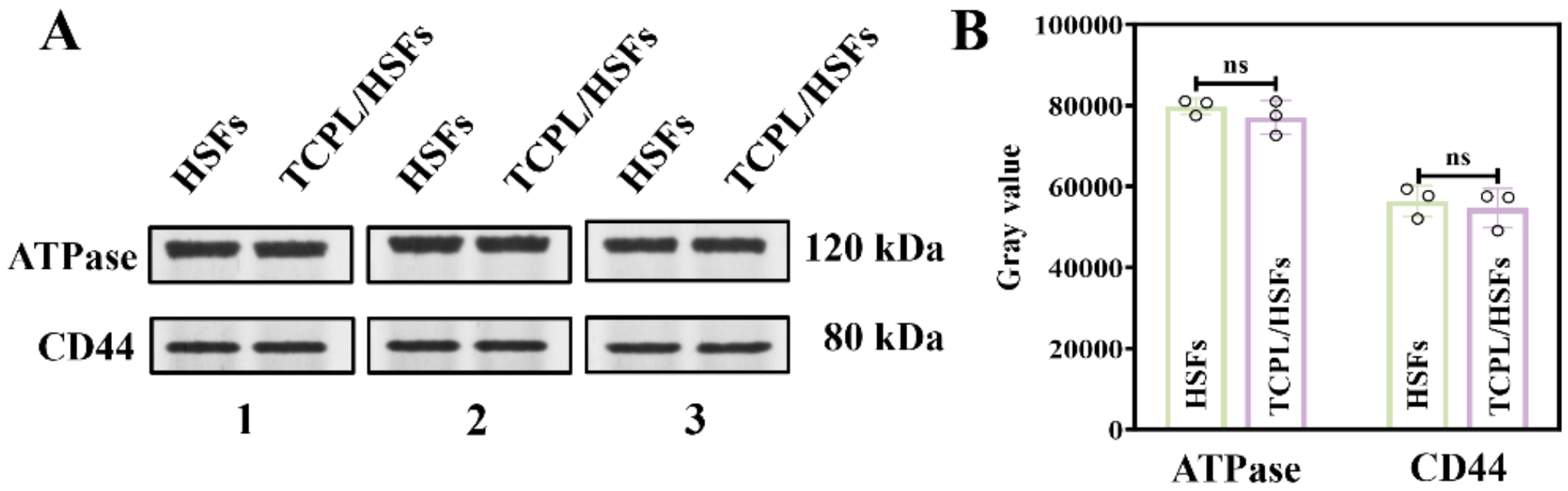

Supplementary Fig. 5. (A) Zeta potential changes of TCPL/HSFs@siTGF-β1 at different mass ratios (w/w=1, 5, 10, 20, 50, 100, n=3, technical replicates). (B) Agarose gel electrophoresis of TCPL/HSFs@siTGF-β1 at different mass ratios (w/w=1, 5, 10, 20, 50, 100, n=3, technical replicates).

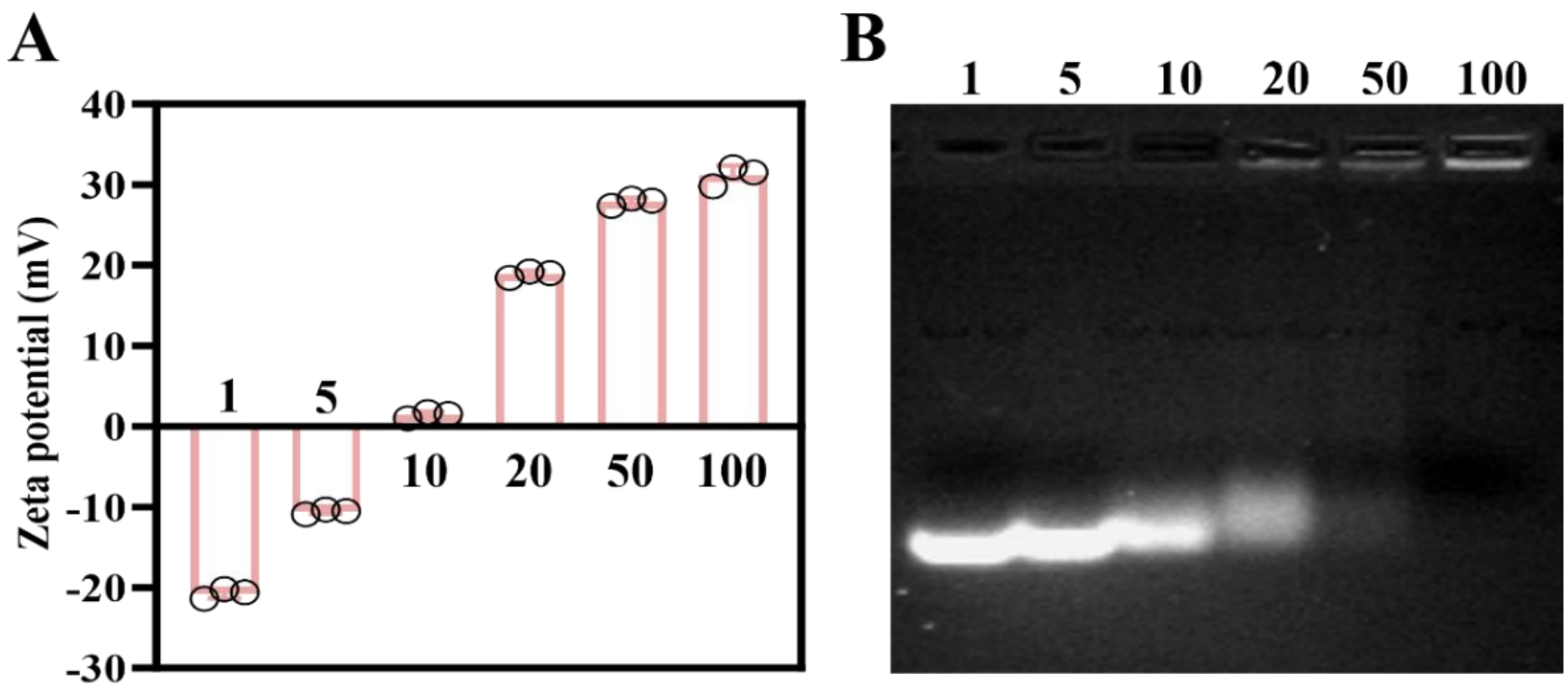

Supplementary Fig. 6. (A) Nanoparticle tracking analysis (NTA) of TCPL/HSFs showing the particle concentration. (B) Calibration curve of Cy5-siRNA concentration versus fluorescence intensity. (C) Quantitative analysis of siRNA loading efficiency in three independently prepared batches of TCPL/HSFs. (D) Agarose gel electrophoresis of siRNA complexed with three independently prepared batches of TCPL/HSFs.

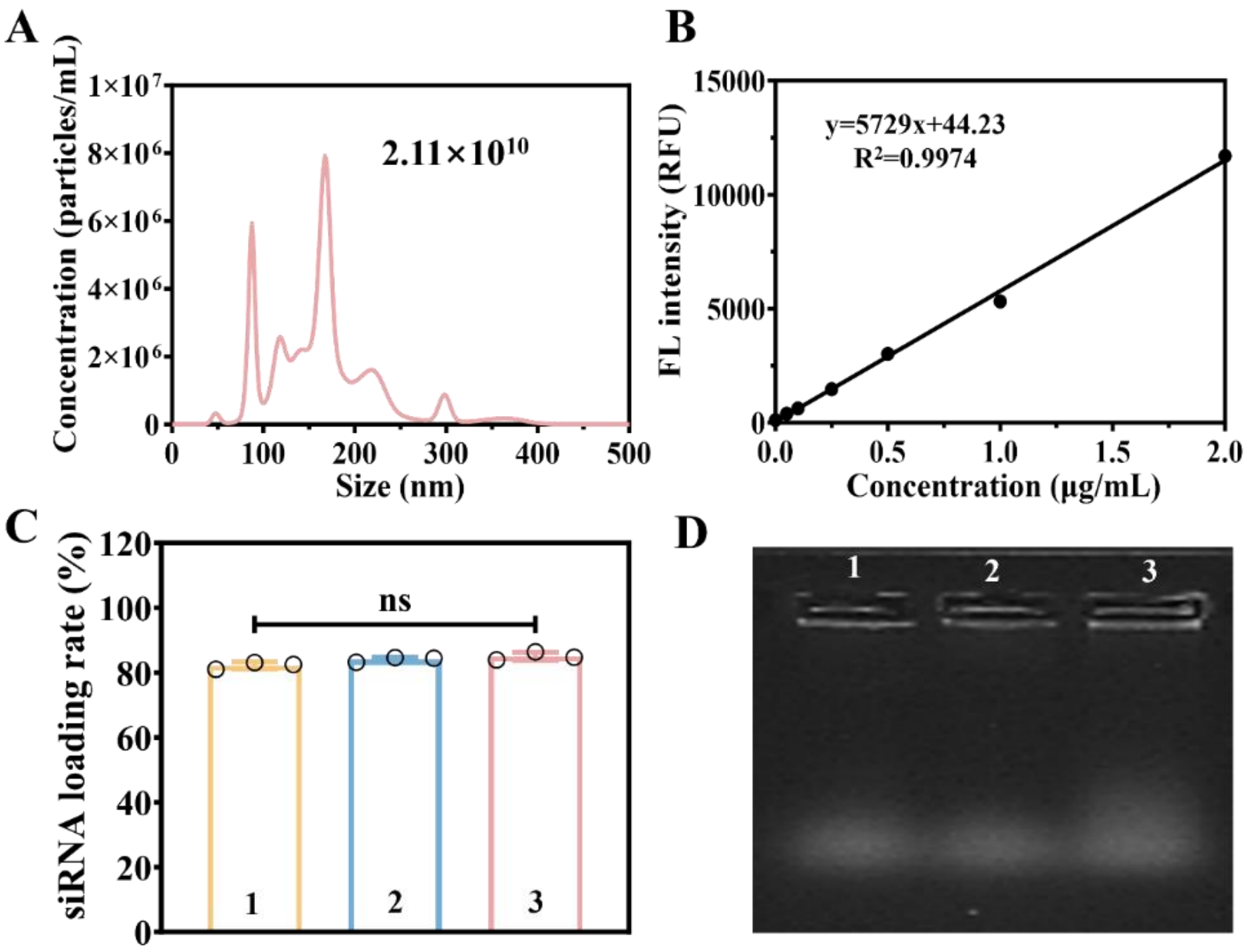

Supplementary Fig. 7. (A) Western blot analysis of TGF-β1 protein expression in HSFs following treatment with 2×dose TCPL/HSFs@siTGF-β1. (B) Densitometric quantification of TGF-β1 protein expression, (n=3, biological replicates). (C) Cell viability of HSFs treated with TCPL/HSFs@siTGF-β1 at the original dose (1×) and twice the original dose (2×), (n=3, biological replicates). ****$P < 0.0001$.

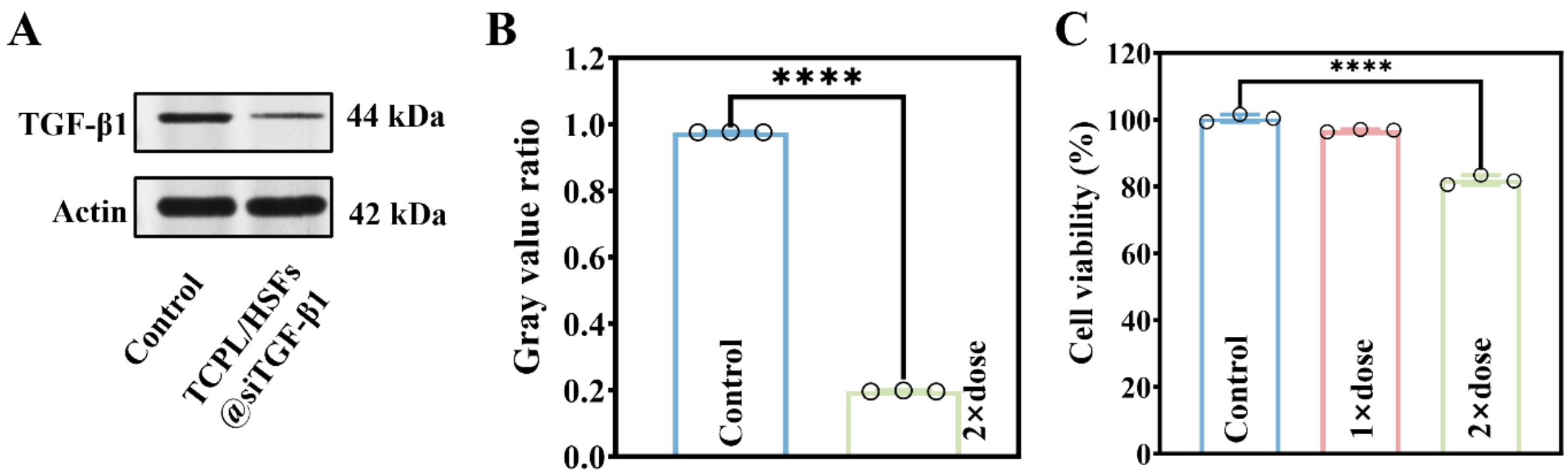

Supplementary Fig. 8. Representative HRTEM images of TCPL/HSFs recovered from the receptor compartment after 24 h of Franz diffusion cell, (scale bar=100 nm).

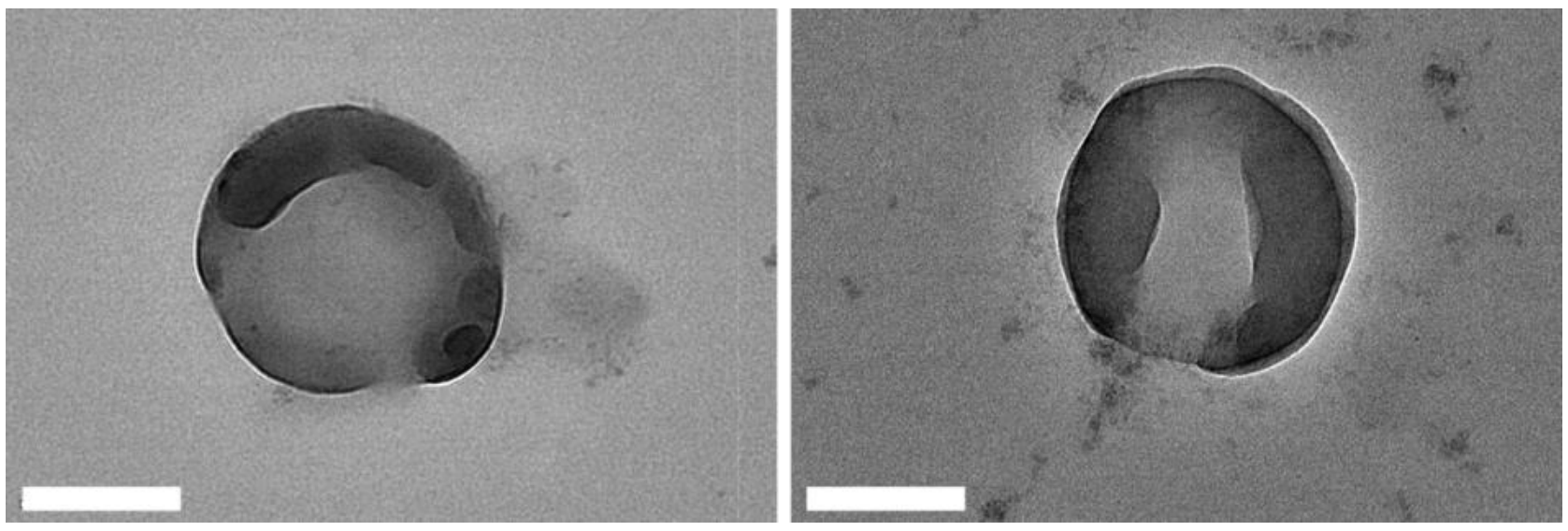

Supplementary Fig. 9. Quantitative analysis of TCPL/HSFs@Cy5-siRNA uptake by HSFs (n=3, biological replicates).

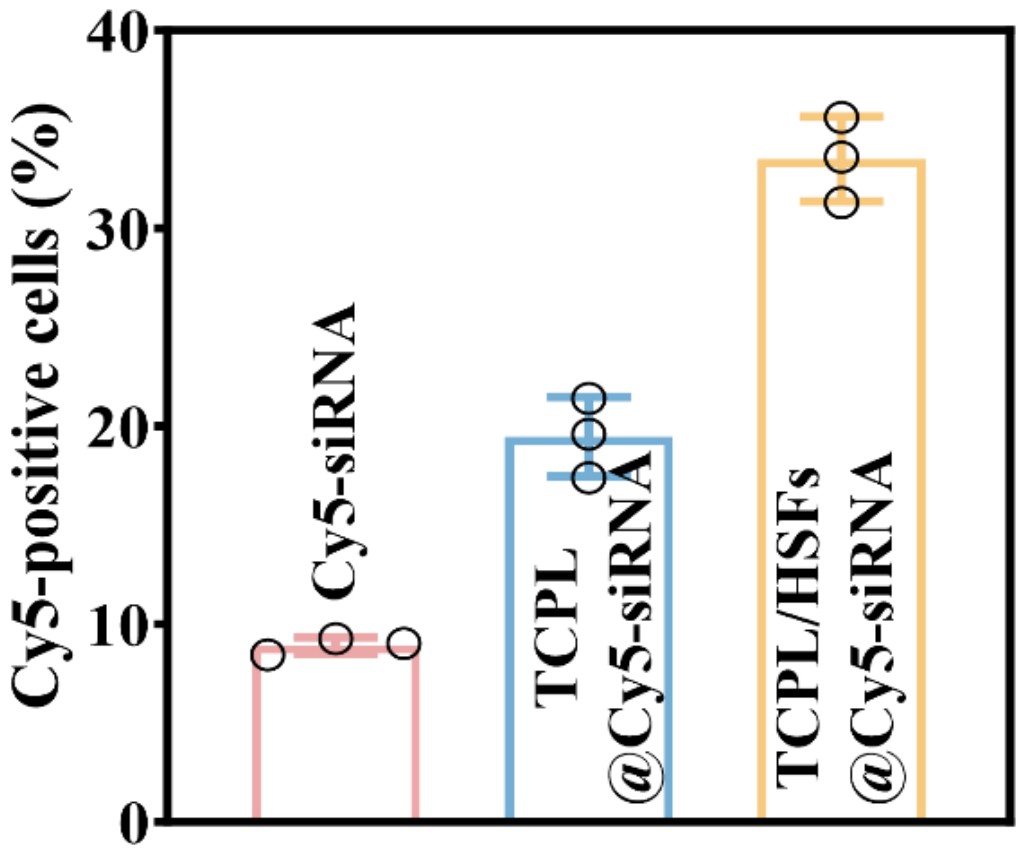

Supplementary Fig. 10. Quantitative uptake analysis of TCPL/HSFs@Cy5-siRNA in HSFs pretreated with HSFs membranes (n=3, biological replicates).

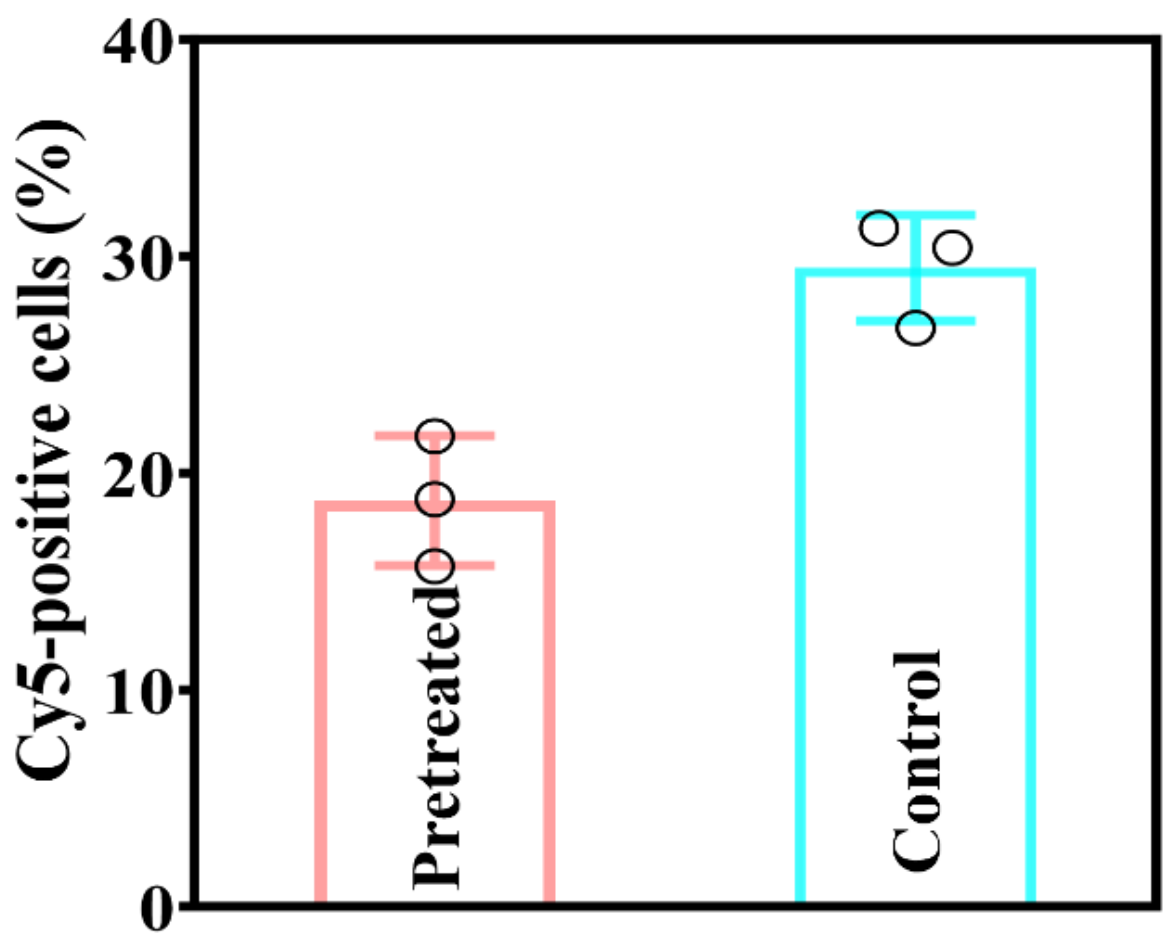

Supplementary Fig. 11. Flow cytometric analysis and quantitative comparison of the uptake of TCPL@Cy5-siRNA、TCPL/HSFs@Cy5-siRNA by THP-1 (n=3, biological replicates).

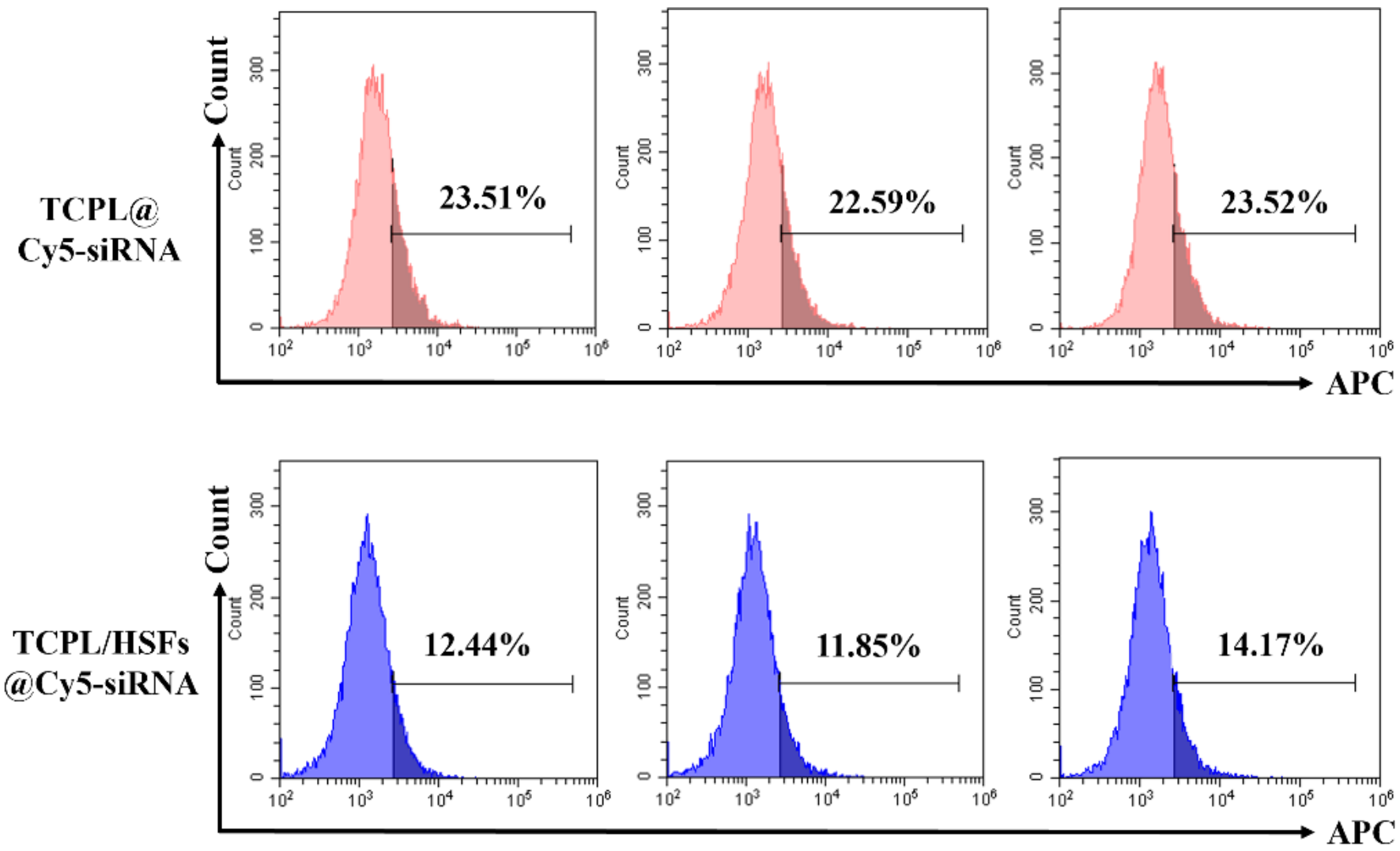

Supplementary Fig. 12. Quantitative analysis of fluorescence intensity along the z-axis of the Matrigel. ****$P < 0.0001$.

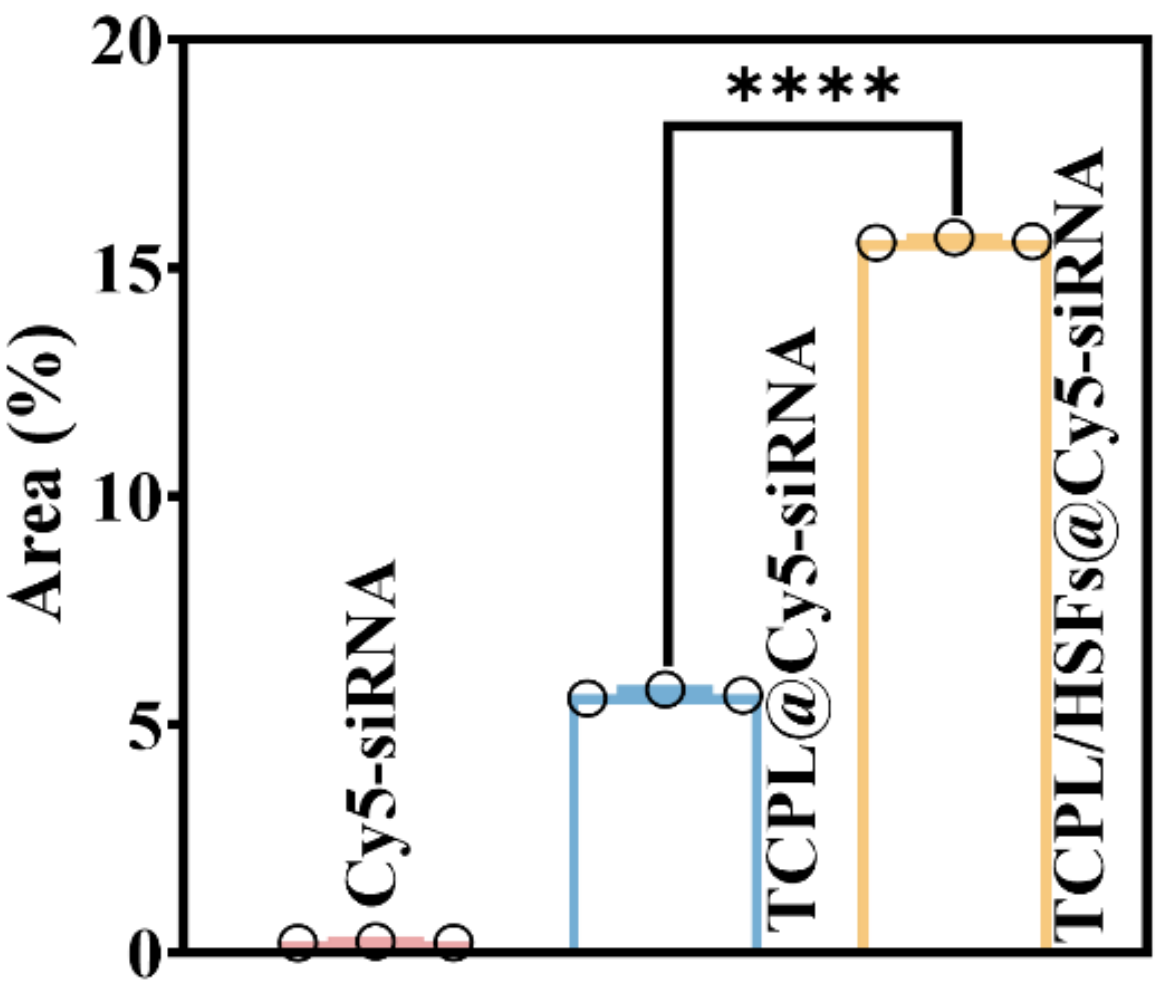

Supplementary Fig. 13. Quantitative analysis of TCPL/HSFs@Cy5-siRNA penetration in mouse dorsal skin. ****$P < 0.0001$.

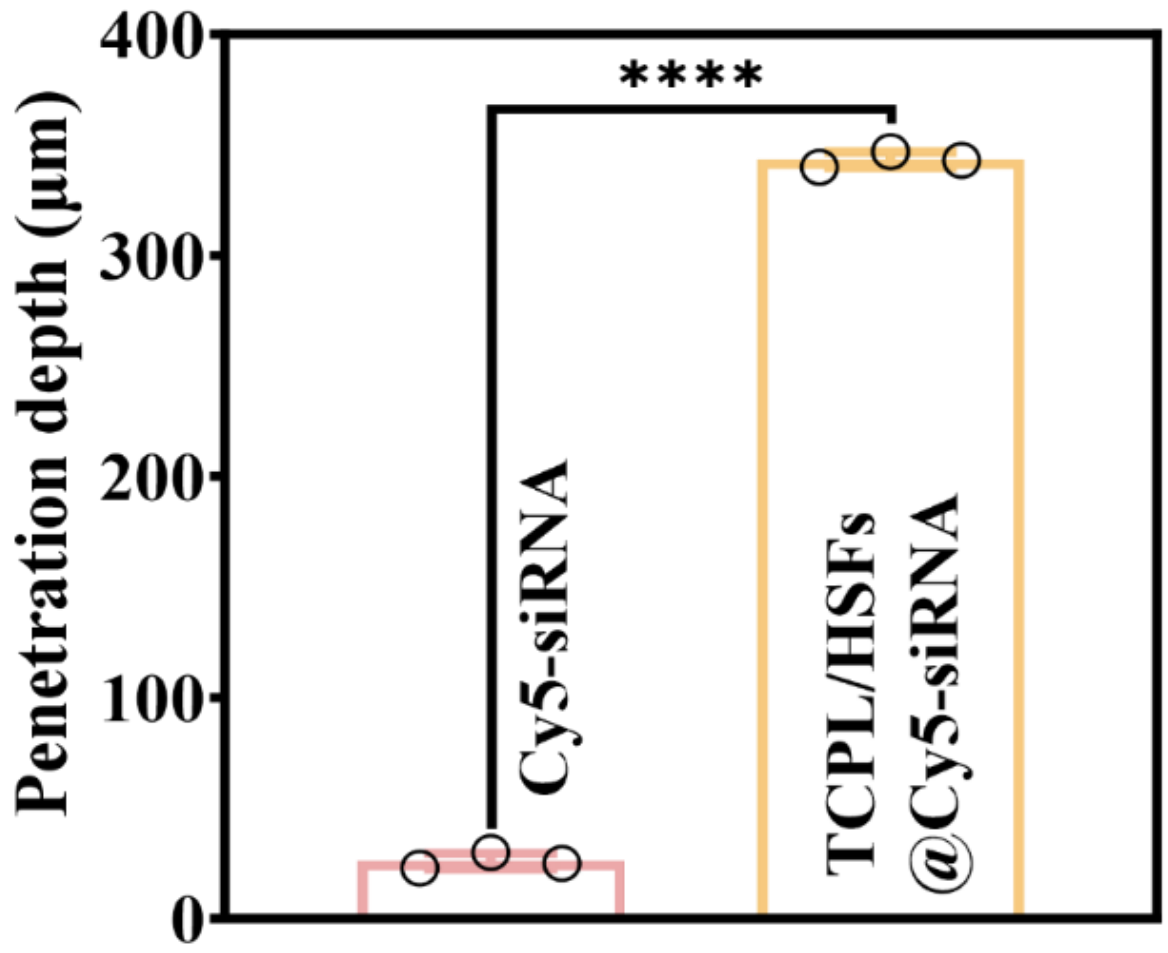

Supplementary Fig. 14. TEER values after removal of TCPL/HSFs@siTGF-β1 and replacement with fresh medium.

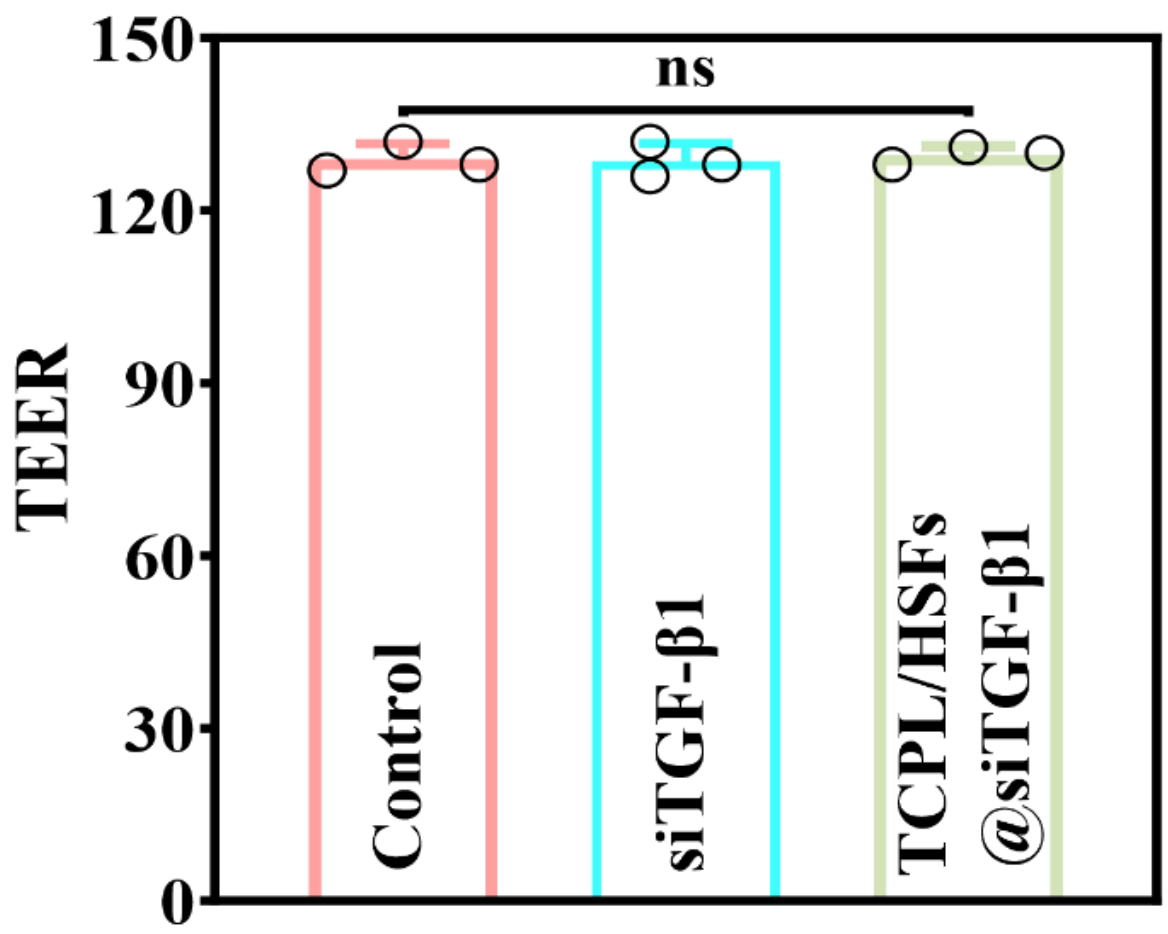

Supplementary Fig. 15. (A) Western blot analysis of ZO-1 protein expression in HaCaT following treatment with TCPL/HSFs and TCPL/HSFs@siTGF-β1. (B) Densitometric quantification of ZO-1 protein expression, (n=3, biological replicates). ****$P < 0.0001$.

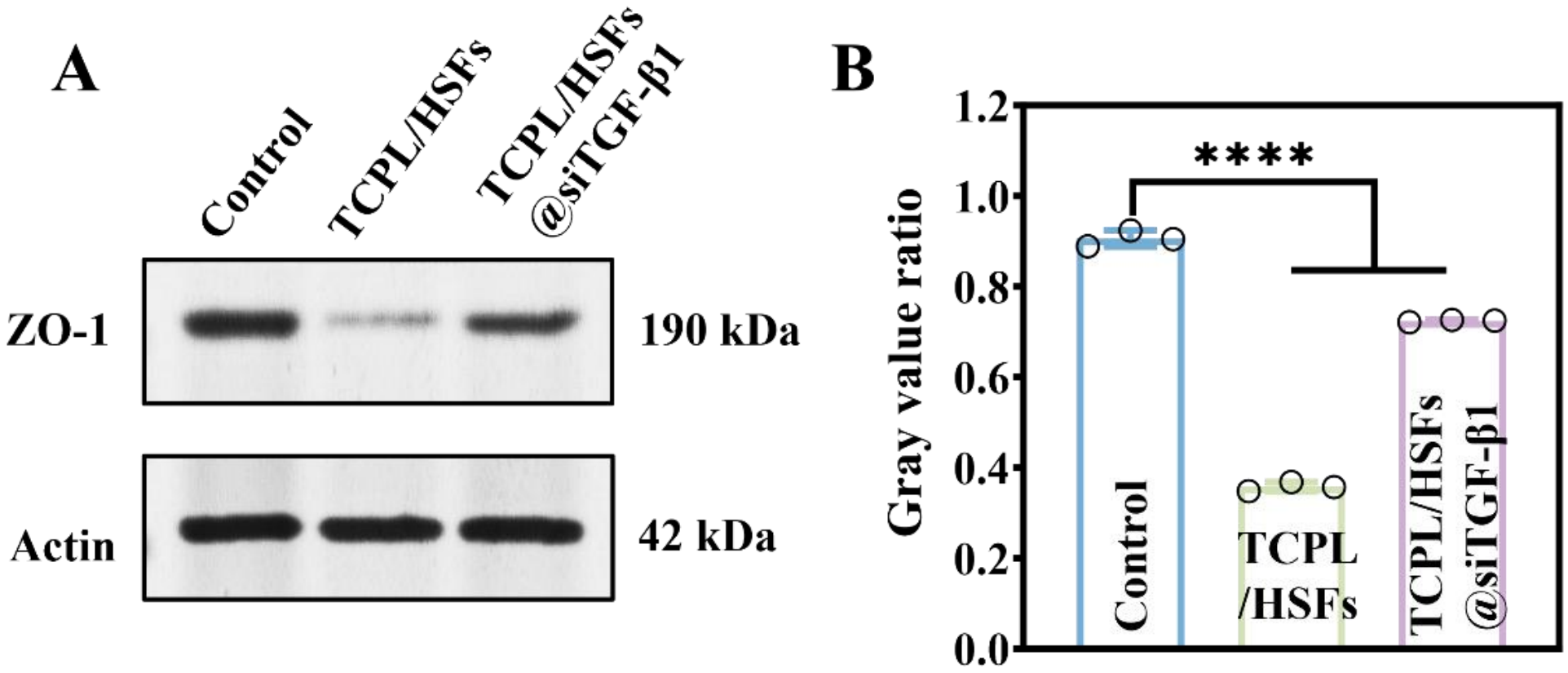

Supplementary Fig. 16. (A–C) Quantitative analysis of transfection efficiency of TCPL/HSFs@GFP in HaCaT, HSFs and HDF. Freq. of Parent represents the percentage of cells within the Cy5-positive gate relative to the total number of cells in its immediately preceding parent gate. (D) Untreated cells and cells treated with TCPL/HSFs without GFP plasmid (n = 3, biological replicates).

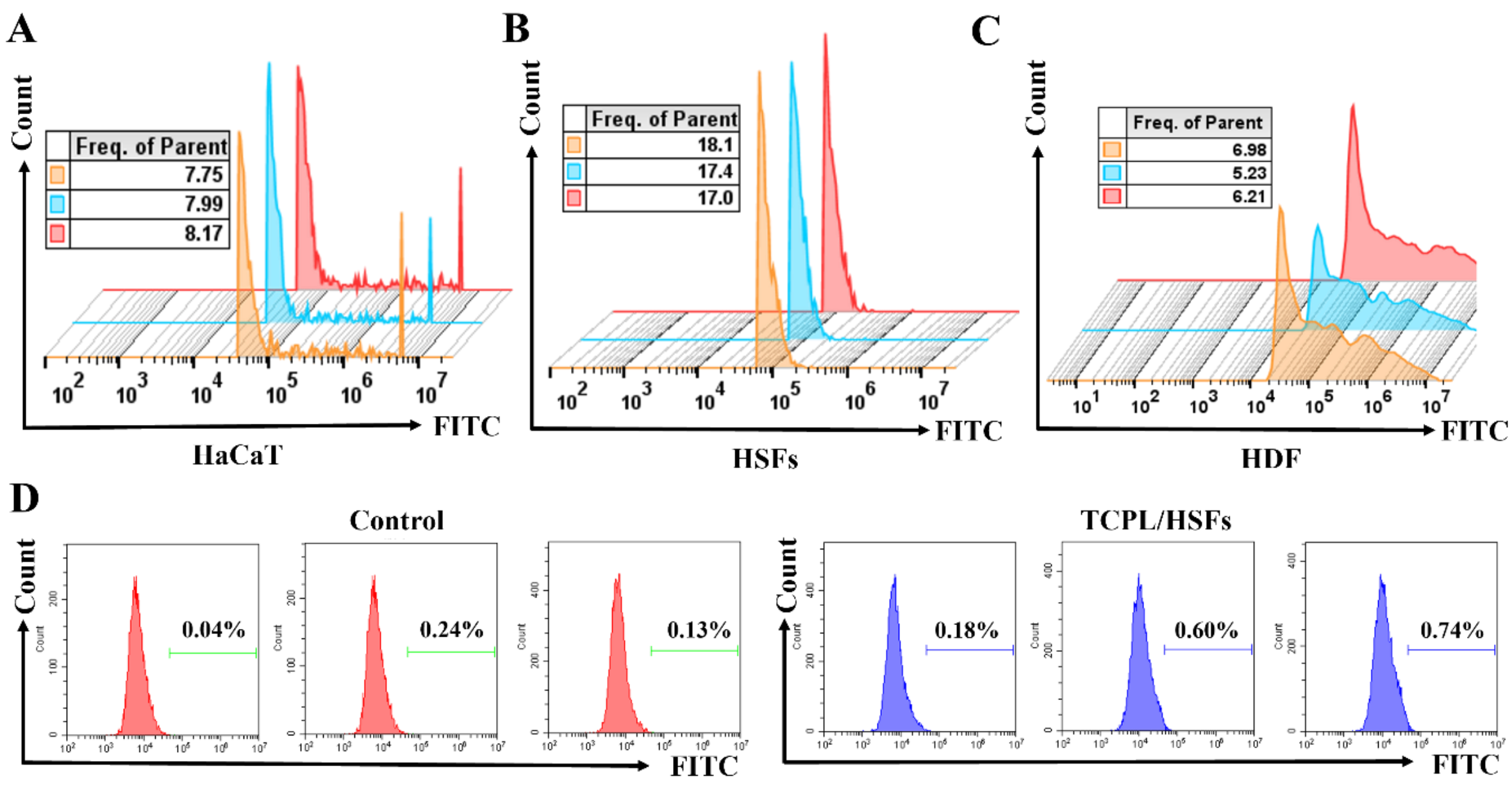

Supplementary Fig. 17. (A) Cell proliferation analysis of TCPL/HSFs in HSFs cells (n=3). (B) Cell viability analysis of TCPL/HSFs in HaCat cells (n=3, biological replicates).

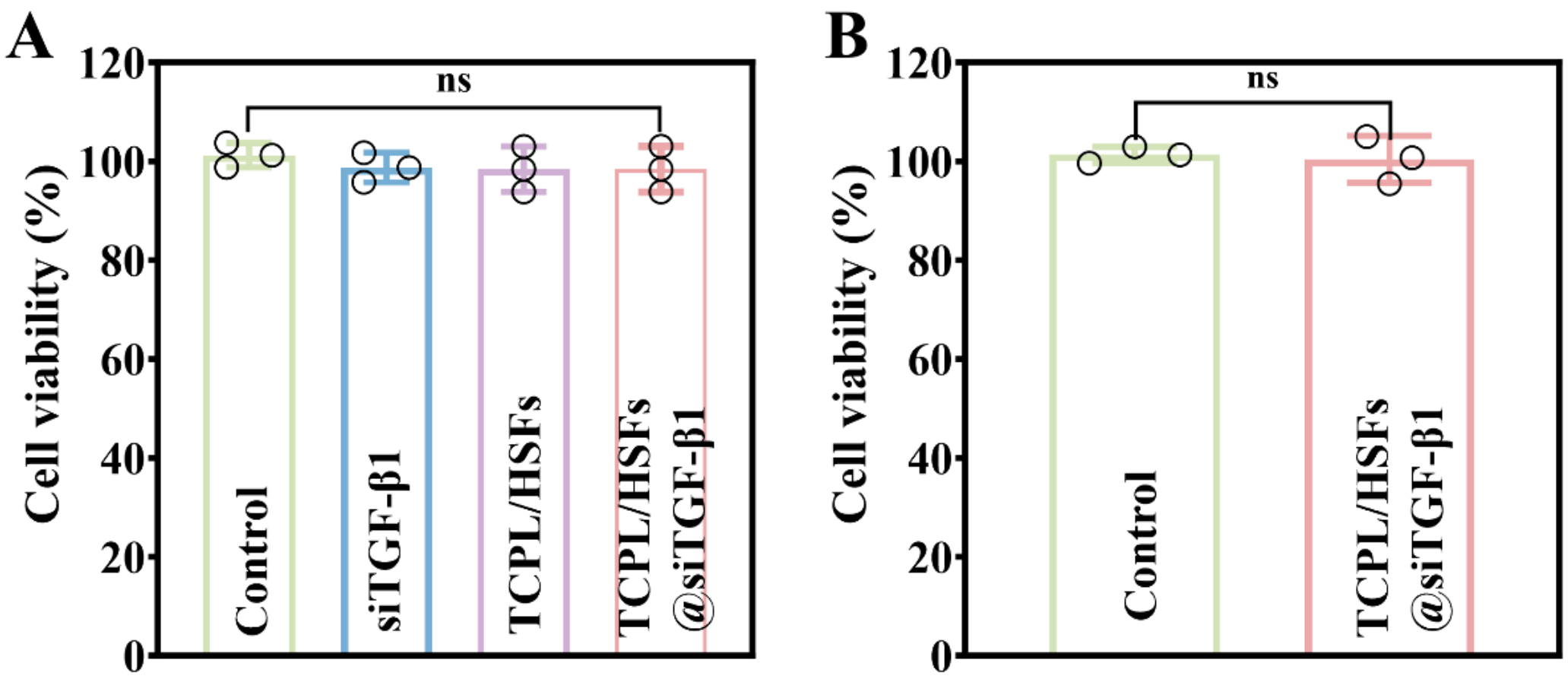

Supplementary Fig. 18. qRT-PCR analysis of fibrosis-related gene expression (COL1A1, ACTA2) in HSFs after different treatments. ****$P < 0.0001$.

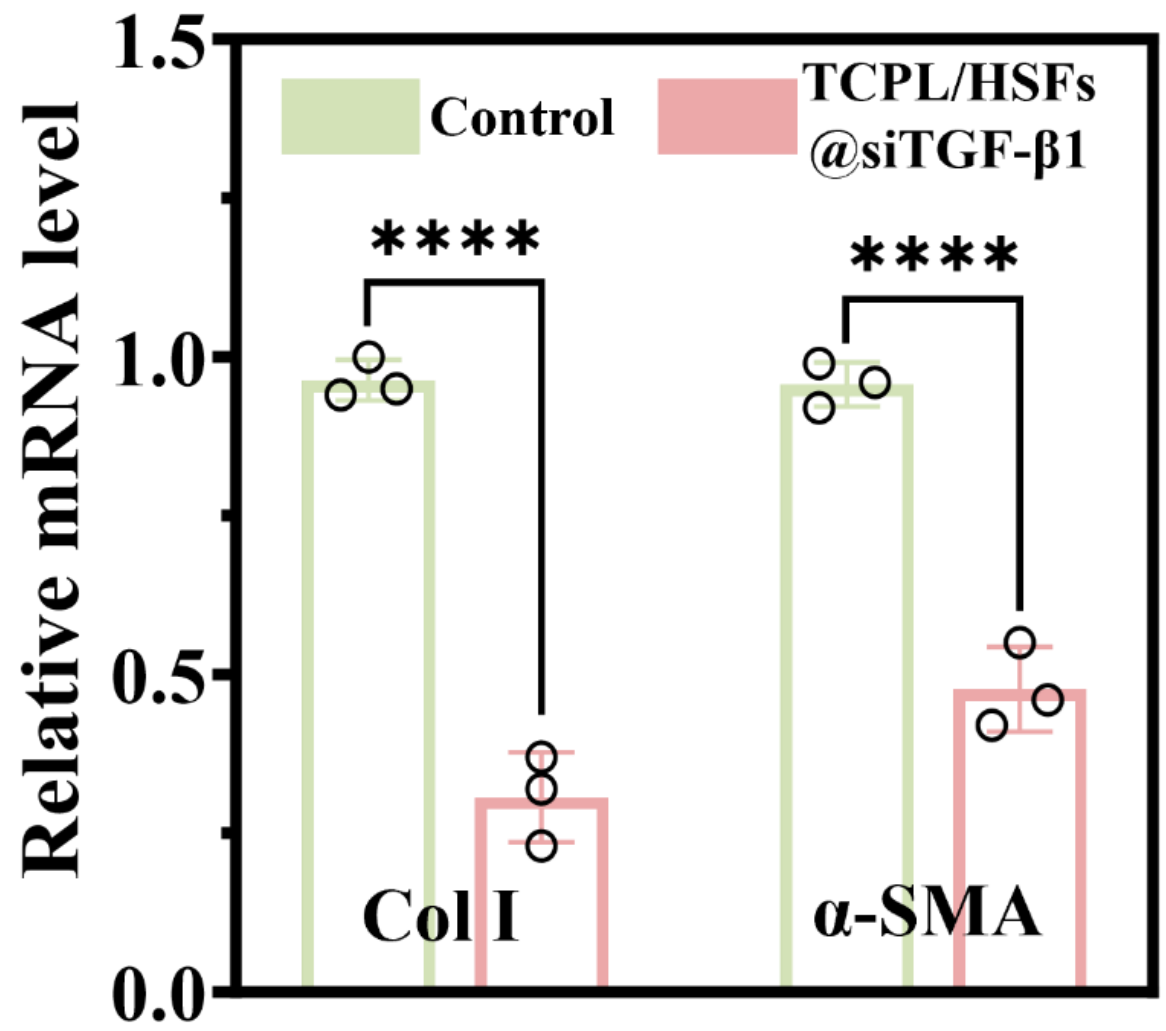

Supplementary Fig. 19. (A) Western blot analysis of TGF-β1 and α-SMA protein expression in HSFs following treatment with TCPL/HSFs and TCPL/HSFs@siTGF-β1. (B) Densitometric quantification of TGF-β1 and α-SMA protein expression, (n=3, biological replicates).

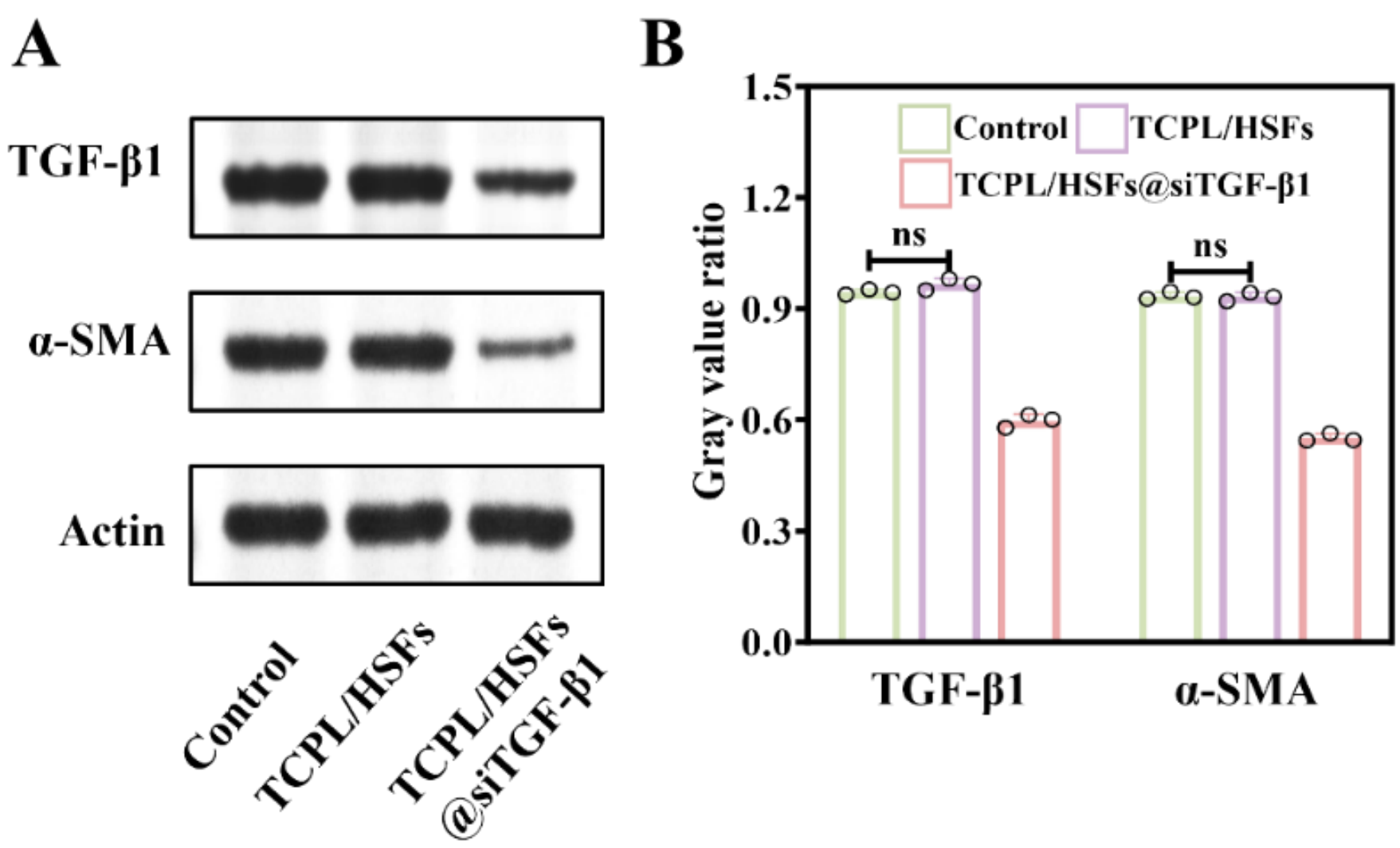

Supplementary Fig. 20. Penetration of TCPL/HSFs@Cy5-siRNA in rabbit ear scar tissue, scale bar=100 μm.

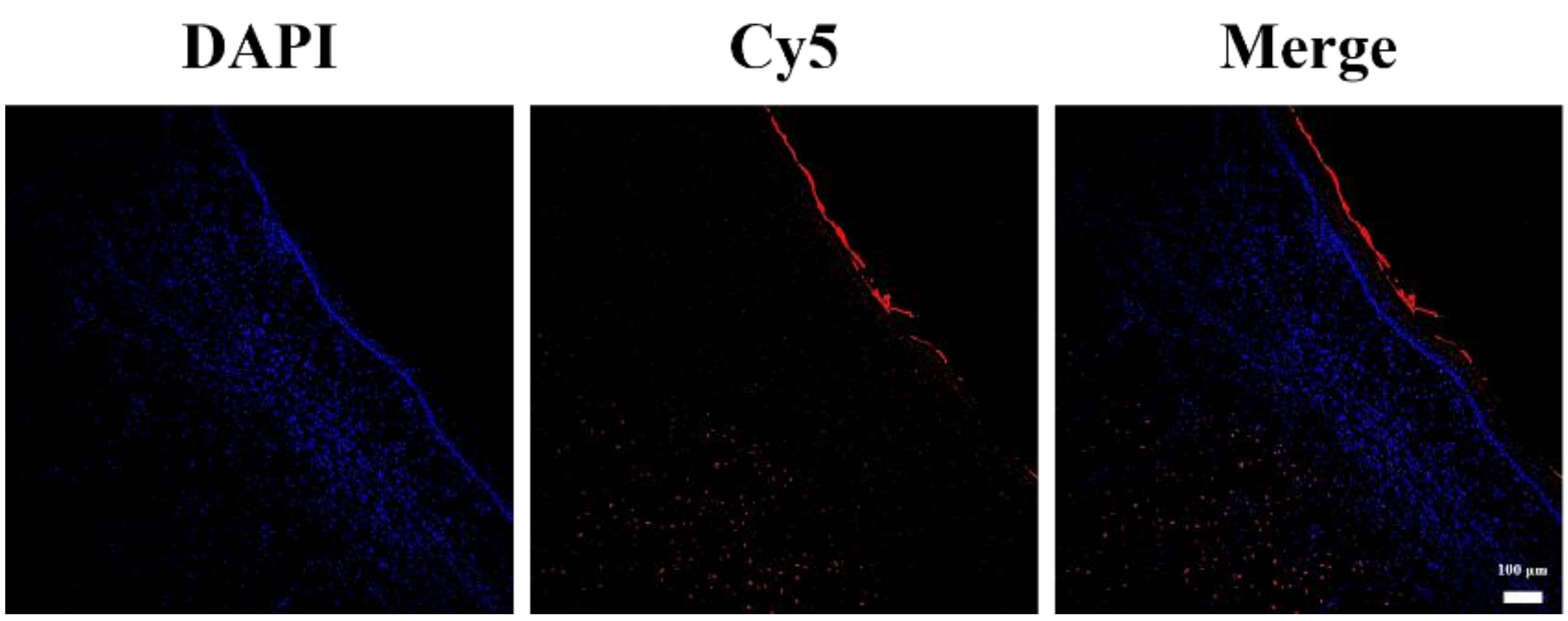

Supplementary Fig. 21. Quantitative analysis of collagen in Masson staining (n=4, biological replicates). ****$P < 0.0001$.

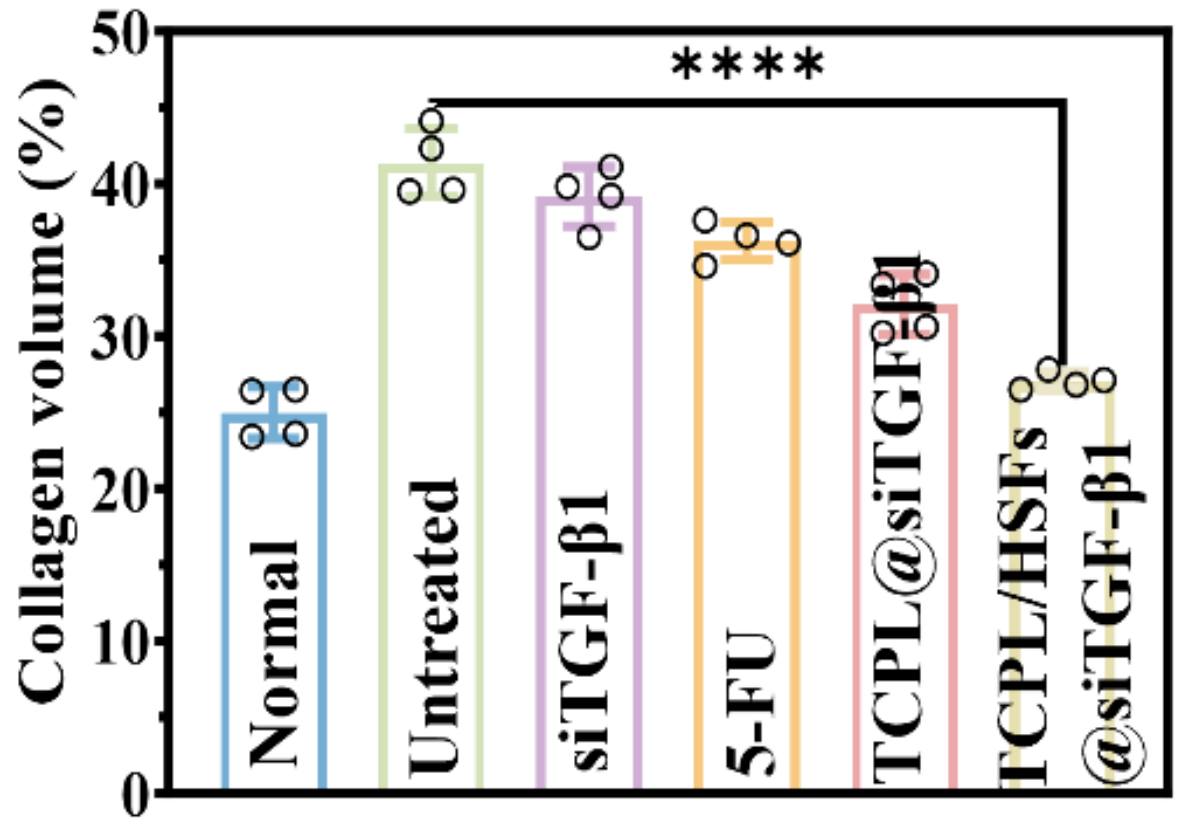

Supplementary Fig. 22. Immunofluorescence staining showing Tunel (red) expression in scar tissues of each group (scale bar=100 μm).

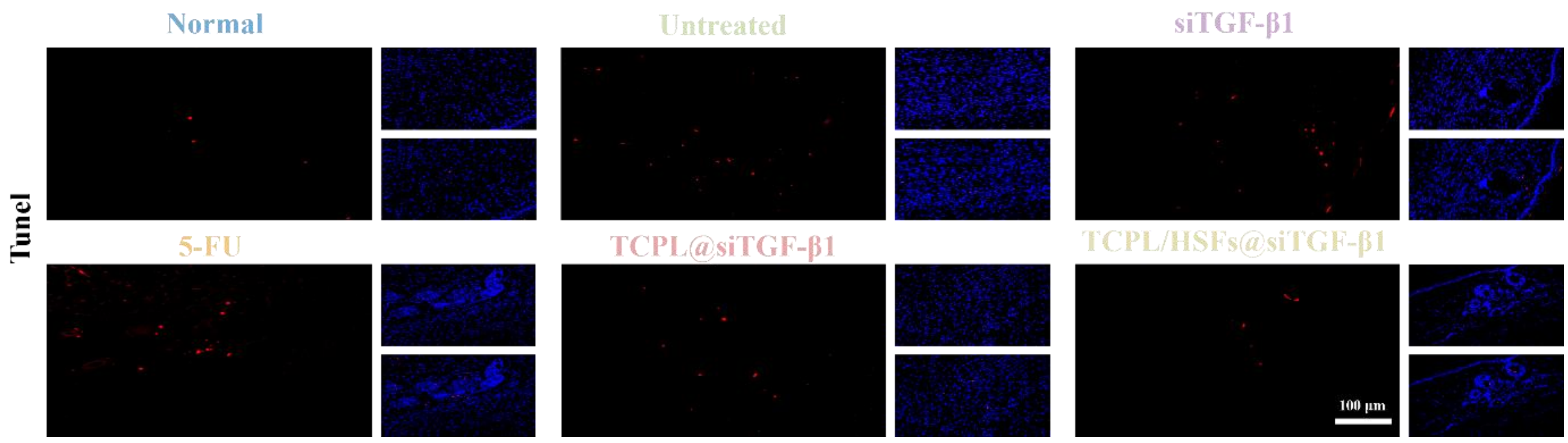

Supplementary Fig. 23. (A-D) Serum levels of ALT, AST, BUN, and creatinine in PBS and TCPL/HSFs groups.

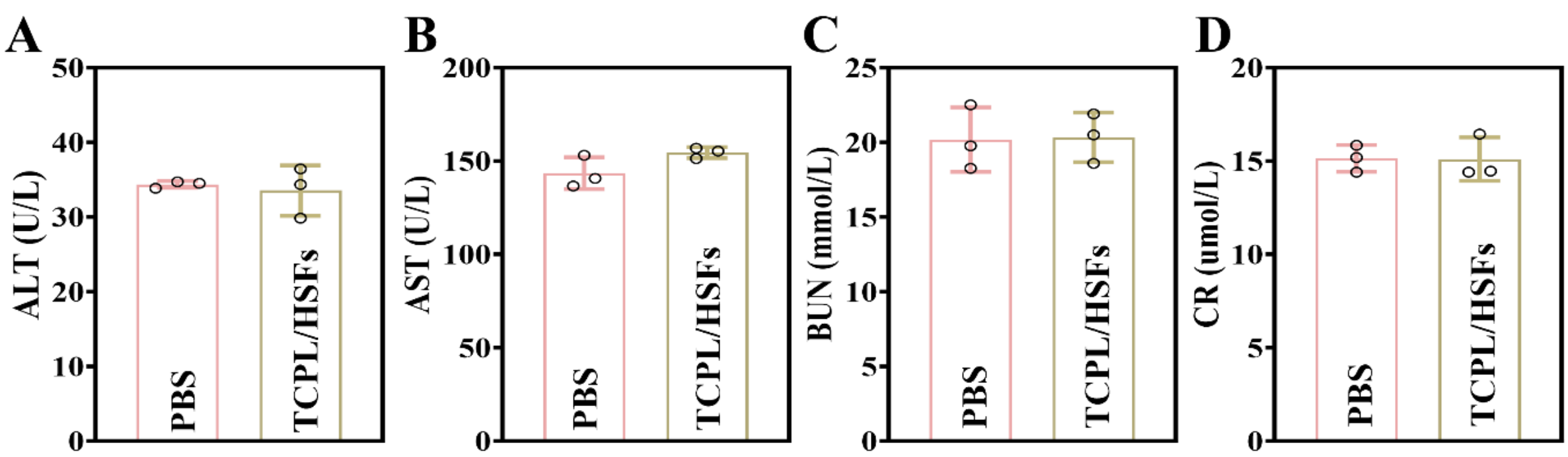

Table. S1. Particle size, PDI, and potential of three independent batches of TCPL/HSFs, respectively, (n=3 technical replicates).

Batch	Hydrodynamic diameter (nm)	PDI	Zeta potential (mV)
1	228.3±5.4	0.17±0.03	26.8±1.93
2	230.2±3.9	0.19±0.04	25.5±0.81
3	233.8±2.9	0.19±0.02	24.2±2.04

Table. S2. Summary of the physicochemical characterization of TCPL, TCPL/HSFs, and TCPL/HSFs@siTGF-β1, (n=3, technical replicates).

Parameter	TCPL	TCPL/HSFs	TCPL/HSFs@siTGF-β1
Hydrodynamic diameter (nm)	245.4 ± 5.0	242.2 ± 2.5	301.4 ± 4.7
Zeta potential (mV)	32.5 ± 0.6	27.8 ± 0.4	14.3 ± 0.5
Membrane protein profile	Not applicable	Positive (SDS-PAGE)	Positive (SDS-PAGE)
Fusion verification	Not applicable	Positive (co-localization, FRET)	EDS
Deformation index	0.43 ± 0.01	0.42± 0.02	inherited carrier property

Table. S3. Stability of TCPL/HSFs@siTGF-β1 during incubation at 37 °C for 5 days, (n=3 technical replicates).

**Time (day)**	**Hydrodynamic diameter (nm)**	**PDI**	**Zeta potential (mV)**
1	294.3±3.9	0.13±0.02	14.3±0.49
2	297.8±2.9	0.14±0.02	15.2±0.38
3	309.4±4.1	0.17±0.01	15.6±0.25
4	314.8±3.2	0.15±0.01	15.3±0.63
5	313.6±3.5	0.17±0.02	15.4±0.46

Table. S4. siRNA sequences.

siRNA	Forward/Reverse	Primer (5' to 3')
TGFβ1-Homo-1225	Forward	CCCACAACGAAAUCUAUGATT
	Reverse	UCAUAGAUUUCGUUGUGGGTT
TGFβ1-Homo-1713	Forward	GCCCUGGACACCAACUAUUTT
	Reverse	AAUAGUUGGUGUCCAGGGCTT
TGFβ1-Homo-2007	Forward	GAGCAGCUGUCCAACAUGATT
	Reverse	UCAUGUUGGACAGCUGCUCTT
TGFβ1-Homo-963	Forward	GGACUAUCCACCUGCAAGATT
	Reverse	UCUUGCAGGUGGAUAGUCCTT
TGFβ1-Rabbit-1676	Forward	CCACCAUUCACAGCAUGAATT
	Reverse	UUCAUGCUGUGAAUGGUGGTT

Table. S5. siRNA sequences.

siRNA	Forward/Reverse	Primer (5' to 3')
Thy1-Homo-221	Forward	CACCCAUCCAGUACGAGUUTT
	Reverse	AACUCGUACUGGAUGGGUGTT
Thy1-Homo-329	Forward	GCAAAUACAACAUGAAGGUTT
	Reverse	ACCUUCAUGUUGUAUUUGCTT
Thy1-Homo-451	Forward	CUCAGAGACAAACUGGUCATT
	Reverse	UGACCAGUUUGUCUCUGAGTT
Thy1-Homo-548	Forward	CCACGGAUUUCAUGUCCCUTT
	Reverse	AGGGACAUGAAAUCCGUGGTT
